# Lymphodepleting preconditioning impairs host antitumor immunity induced by adoptive T cell therapy in mouse models

**DOI:** 10.1038/s41467-026-71082-y

**Published:** 2026-03-31

**Authors:** Diego Figueroa, Juan Pablo Vega, Andrés Hernández-Oliveras, Felipe Ardiles, Sofía Hidalgo, Ximena López, Vicente Saavedra, Catalina Bustamante, Daniela Malavé, Felipe Flores, Felipe Gálvez-Cancino, Hugo Gonzalez, Manuel Varas-Godoy, Maria Rosa Bono, Fabiola Osorio, Vincenzo Borgna, Alvaro Lladser

**Affiliations:** 1 Centro Basal Ciencia & Vida, Fundación Ciencia & Vida, Santiago, Chile; 2 Centro de Investigación e Innovación en Cáncer, Fundación Arturo López Pérez OECI Cancer Center, Santiago, Chile; 3 Laboratory of Immunology and Cellular Stress, Facultad de Medicina, Universidad de Chile, Santiago, Chile; 4 Laboratory of Immune Regulation, NDM Centre for Immuno-Oncology, University of Oxford, Oxford, UK; 5 Department of Anatomy, University of California San Francisco, San Francisco, CA USA; 6 Centro de Biología Celular y Biomedicina (CEBICEM), Facultad de Ciencias, Universidad San Sebastián, Santiago, Chile; 7 Laboratory of Immunology, Facultad de Ciencias, Universidad de Chile, Santiago, Chile; 8 Servicio de Urología, Hospital Barros Luco Trudeau, Santiago, Chile; 9 Facultad de Medicina, Universidad San Sebastián, Santiago, Chile

**Keywords:** Immunotherapy, Tumour immunology

## Abstract

Adoptive T cell therapy (ACT) is effective against hematologic cancers, but the mechanisms underlying durable responses in solid tumors remain unclear. We show that adoptively transferred CD8^+^ T cells that eradicate established murine tumors promote expansion of host CD8^+^ T cells exhibiting tumor-reactive and tissue-resident phenotypes that contribute to tumor elimination. Mechanistically, tumor necrosis factor (TNF) from transferred cells induces dendritic cell (DC)-dependent expansion of host CD8^+^ T cells, conferring protection against ACT-resistant tumor cells lacking the targeted antigen. Lymphodepleting preconditioning promotes expansion of transferred cells and primary tumor eradication but impairs host antitumor immunity and abrogates protection against ACT-resistant tumors. In human tumors, increased TNF/DC/CD8^+^ T cell profiles correlate with favorable ACT responses and improved survival. These findings reveal a TNF-dependent interplay between transferred and host CD8^+^ T cells underlying durable antitumor immunity that is impaired by lymphodepleting preconditioning in mouse models, suggesting an underappreciated mechanism of ACT resistance.

## Abstract (web summary)

Adoptive cell therapy has shown success in the context of haematological cancer but is associated with limited effect with solid tumors. Here the authors show that lymphodepletion regimens enhance tumor eradication by adoptive cell therapy, yet impair host antitumor immune response required for durable protection in murine models of cancer.

## Introduction

Antitumor immunity is largely mediated by tumor-specific CD8^+^ T cells, which are activated in the lymph nodes by migratory conventional type 1 dendritic cells (cDC1) cross-presenting tumor antigen-derived peptides onto major histocompatibility complex (MHC) class I molecules^1–3^. CD8^+^ T cells that recognize tumor antigen/MHC-I complexes through their T cell receptor (TCR), proliferate, and differentiate into cytotoxic CD8^+^ T cells that then migrate to tumors, where they recognize target cancer cells and kill them by releasing cytotoxic molecules, such as perforin, granzymes, and granulysin^4^. In addition, they also secrete effector cytokines, such as interferon gamma (IFNγ) and tumor necrosis factor (TNF), which promote antigen presentation on target cells and activate myeloid cells, including macrophages and DCs^5,6^. In the tumor microenvironment, chronic TCR stimulation induces a dysfunctional differentiation program in tumor-specific CD8^+^ T cells, known as T cell exhaustion, which is characterized by the expression of the transcription factor TOX and multiple inhibitory receptors, such as PD-1^7,8^. Exhausted CD8^+^ T cells comprise at least two functionally distinct populations: progenitor-exhausted (Tpex) and terminally-exhausted (Tex) cells (9,10). The Tpex subset expresses the transcription factor TCF-1, has self-renewal potential, and upon antigen-mediated TCR activation, gives rise to Tex cells. Tex cells are terminally differentiated and characterized by the up-regulation of cytotoxic molecules and the inhibitory receptor TIM3, but lack TCF-1 and self-renewal potential^9^. In addition, both Tpex and Tex populations acquire a tissue-resident transcriptional program that allows them to adapt and establish in different microenvironments^10^. Accumulating evidence indicates that an effective antitumor immunity relies on the concerted action of both Tpex (PD-1^+^TOX^+^TCF-1^+^GzmB^-^TIM3^−^) and Tex (PD-1^high^TOX^+^TCF-1^−^GzmB^+^TIM3^+^) cells^11–13^. Interestingly, better clinical outcomes depend on the ability of Tpex cells to proliferate and differentiate into Tex cells, leading to their intratumor accumulation^14^. All these studies provide evidence supporting that the Tpex pool is important for long-term establishment in tumors, whereas Tex cells are critical for exerting acute cytotoxic antitumor activity.

Adoptive cell therapy (ACT), which consists of the infusion of autologous tumor-specific T cells expanded ex vivo, has emerged as a novel treatment for hematological cancers and has shown promise in the treatment of solid tumors^15^. ACT using tumor-infiltrating lymphocytes (TILs) has been shown to induce therapeutic activity with curative potential in patients with melanoma and other solid tumors^16,17^. However, the implementation of reproducible TIL therapies remains challenging due to the low frequency, dysfunctional state, and limited proliferation of tumor-reactive T cells^18^. On the other hand, ACT strategies using peripheral blood-derived T cells genetically engineered to express tumor-specific TCR (TCR-T) or chimeric antigen receptors (CAR-T) have overcome these limitations and have demonstrated potent antitumor responses^19,20^. CAR-T cell immunotherapies have shown remarkable success in patients with different hematological cancers, including subtypes of leukemia, lymphoma, and myeloma^21,22^. Currently, six different CAR-T cell immunotherapies are approved by the F.D.A. for hematologic cancers, but none for solid tumors^23^. TCR-T ACTs have been tested in clinical studies for almost two decades, showing safety and promising antitumor responses in patients with metastatic melanoma and other solid tumors^24^.

An important factor contributing to ACT efficacy is the persistence of transferred T cells in vivo^25^. Consequently, strategies aimed at improving T cell persistence have been extensively used. Non-myeloablative lymphodepletion as a preconditioning regimen, such as chemotherapy and/or radiation, is an integral part of currently used clinical protocols. Such regimens favor the engraftment of transferred T cells, probably by attenuating the competition between infused and endogenous T cells for cytokines and a niche^26^. Despite significant advances in the field, most cancer patients with solid tumors fail to respond to ACT or develop resistance through different mechanisms. The emergence of mutant tumor cells that lack the expression of targeted antigens is a major mechanism of resistance to ACT^27–29^. Antigen loss can occur due to several mechanisms, ranging from genetic to post-translational alterations^30–32^. In this scenario, ACT acts as a selective pressure that leads to the selection of tumor cell clones with decreased immunogenicity, e.g., lacking the target antigen^33^. Hence, strategies stimulating the host immune system can help to overcome these mechanisms of ACT resistance^34^. In fact, combining ACT with oncolytic viruses, Toll-like receptor agonists, and/or DC activators that expand endogenous tumor-specific CD8^+^ T cells can provide long-lasting tumor protection and overcome the appearance of antigen-loss escape variants^35,36^.

Emerging evidence indicates that adoptively transferred T cells can directly stimulate the host tumor microenvironment to promote effective tumor control in preclinical models via IFNγ, which supports the cytotoxic potential of both transferred and endogenous T cells and promotes tumor cell killing^37,38^. However, the mechanisms underlying the crosstalk between transferred and host T cells are incompletely understood. Furthermore, how lymphodepleting preconditioning impacts host antitumor immunity to control primary tumors and antigen-loss escape variants remains largely unexplored. Here, we show that ACT with TCR-transgenic T cells eradicates established tumors and expands host CD8^+^ T cells exhibiting tumor-reactive and tissue-resident phenotypes, which contribute to primary tumor elimination. Mechanistically, ACT induced TNF- and cross-presenting cDC1-dependent expansion of host CD8^+^ T cells that conferred protection against rechallenge with ACT-resistant tumor cells lacking the targeted antigen. Importantly, even if lymphodepleting preconditioning enhanced expansion of transferred cells and ACT-mediated primary tumor eradication, it impaired host antitumor immunity and abrogated protection against ACT-resistant tumor cells. Interestingly, enrichment of profiles associated with TNF signaling, cDC1, and tumor-reactive CD8^+^ T cells in human melanoma tumors correlated with favorable responses to ACT and increased survival. Our findings reveal a TNF-dependent interplay between transferred and host CD8^+^ T cells that determines effective tumor elimination and durable immunity capable of controlling antigen-loss variants. Notably, this immunity is undermined by lymphodepleting preconditioning, which may ultimately favor acquired resistance to ACT.

## Results

### Effective ACT promotes the expansion of host CD8^+^ T cells exhibiting tumor-reactive and tissue-resident phenotypes

**Fig. 1:**
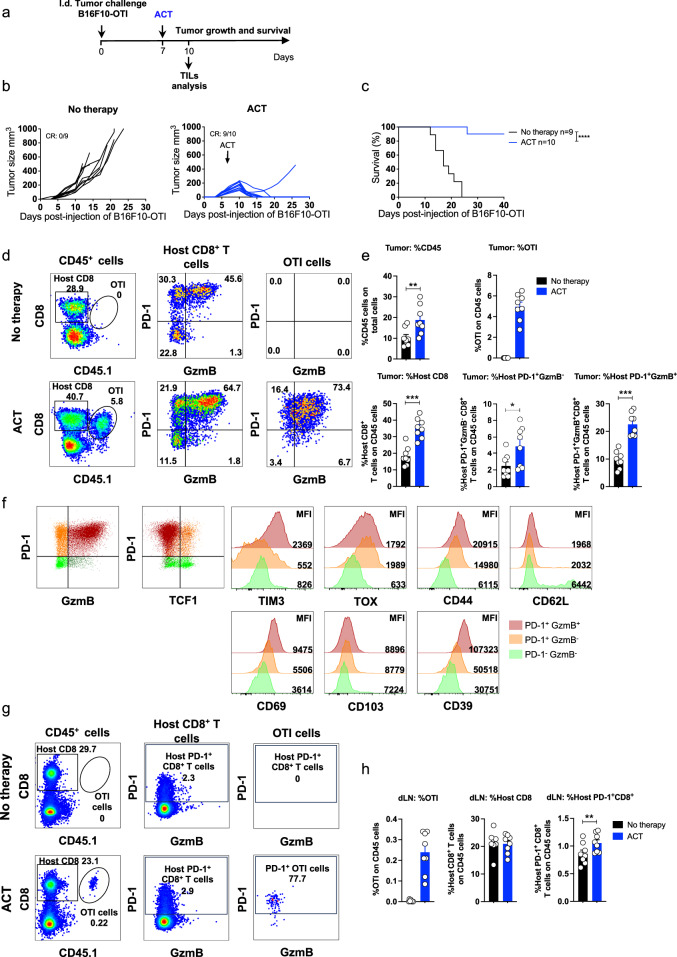
ACT eradicates established melanoma tumors and induces the expansion of host CD8^+^ T cells exhibiting tumor-reactive and tissue-resident phenotypes. C57BL/6 mice bearing B16F10-OTI tumors received i.v. transfer of 1 × 10^6^ in vitro-activated OTI CD8^+^ T cells (ACT). Untreated mice (No therapy) were used as controls. **a** Experimental scheme. Individual tumor growth (**b**) and Kaplan–Meier (**c**) curves for each group: No therapy (black curve), and ACT (blue curves). **d**–**f** Tumor and draining lymph node (dLN) infiltrates were analyzed by flow cytometry three days after ACT. **d** Left panels: Representative plots displaying the frequencies of host (CD45.1^−^) and transferred (OTI CD45.1^+^) CD8^+^ T cells in live CD45^+^ cells and their PD-1 and GzmB expression. Middle panels: PD-1 and GzmB expression in host CD8^+^ T cells and the frequencies of each subpopulation: PD-1^+^GzmB^−^, PD-1^+^GzmB^+^, PD-1^−^GzmB^−^, and PD-1^−^GzmB^+^. Right panels: PD-1 and GzmB expression in transferred OTI CD8^+^ T cells. **e** Quantifications of CD45^+^ cells (percentage of total live cells), transferred OTI CD8^+^ T cells, and PD-1^+^GzmB^-^ and PD-1^+^GzmB^+^ host CD8^+^ T cells as percentages of live CD45^+^ cells. **f** Representative plot displaying PD-1 and GzmB expression in host CD8^+^ T cells. Quadrants define PD-1^+^GzmB^-^ (orange), PD-1^+^GzmB^+^ (red), and PD-1^−^GzmB^−^ (light green). These subpopulations are further characterized by PD-1 and TCF-1 expression and histograms of TIM3, TOX, CD44, CD62L, CD69, CD103, and CD39. **g** Left panels: Representative plots displaying the frequencies of host (CD45.1^−^) and transferred (OTI CD45.1^+^) CD8^+^ T cells in live CD45^+^ cells. Middle panels: PD-1 and GzmB expression in host CD8^+^ T cells. Right panels: PD-1 and GzmB expression in transferred OTI CD8^+^ T cells. **h** Quantifications of OTI, host CD8^+^ T cells, and PD-1^+^CD8^+^ T cells as a percentage of CD45^+^ live cells. **c** Survival Kaplan–Meier curves from two independent experiments. Statistical significance was assessed using a two-sided log-rank Mantel–Cox test ****P < 0.0001. **e**, **h** Pooled data from two independent experiments, No therapy *n* = 8; ACT *n* = 8. Bars are the mean ± SEM. Statistical significance was assessed using two-sided unpaired Mann–Whitney test *P < 0.05, **P < 0.01, ***P < 0.001, ****P < 0.0001. CR complete response.

To investigate the mechanisms underlying durable antitumor immunity induced by ACT, we established a TCR-ACT model consisting of intravenous administration of in vitro-activated OTI TCR-transgenic CD8^+^ T cells to mice bearing B16F10-OTI melanoma tumors (~50–200 mm^3^) engineered to express the ovalbumin-derived epitope OTI (SIINFEKL)^39^ (Fig. 1a). In this setting, tumors were efficiently eliminated within approximately ten days, providing long-term survival (Fig. 1b, c). To evaluate the immune responses driving ACT-mediated tumor elimination, we performed flow cytometry analysis of tumor and draining lymph node (dLN) infiltrates three days after transfer, during the phase of active tumor rejection (Fig. 1d–h). Phenotypic analysis of tumors revealed the increase of immune infiltrates, in particular the expansion of host CD8⁺ T cells expressing PD-1: PD-1^+^GzmB^−^, and PD-1^+^GzmB^+^ (Fig. 1d, e), suggesting active priming of the host antitumor immune response. Transferred OTI cells exhibited a similar phenotype in terms of PD-1 and GzmB. Further phenotypic analysis shows that the PD-1^+^GzmB^-^ subset primarily, but not exclusively, corresponds to PD-1^+^TCF-1^+^TOX1^+^TIM3^+^ progenitor-exhausted (Tpex) cells (Fig. 1f). Conversely, the PD-1^+^GzmB^+^ subset corresponds to PD-1^+^TCF-1^−^TOX1^+^TIM3^+^ terminally differentiated/exhausted (Tex) CD8^+^ T cells (Fig. 1f). These two phenotypes define tumor-reactive CD8^+^ T cells that mediate antitumor immunity and predict favorable response to immunotherapy^12^. In addition, PD-1^+^GzmB^−^ and PD-1^+^GzmB^+^ host CD8⁺ T cells expressed CD44, CD69, and CD39, markers associated with tissue-resident memory T cells in mouse models^40^. In the draining lymph nodes, PD-1^+^GzmB^−^ CD8⁺ T cells were also expanded following ACT (Fig. 1g, h). In contrast, a suboptimal ACT protocol that initially reduces tumor growth but is unable to eliminate tumors did not expand host CD8^+^ T cells (Supplementary Fig. 1). Altogether, these findings suggest that ACT potently eliminates tumors and promotes the expansion and differentiation of host tumor-specific CD8⁺ T cells that may contribute to long-term tumor control.

### Host CD8^+^ T cells contribute to ACT-mediated eradication of primary tumors

**Fig. 2:**
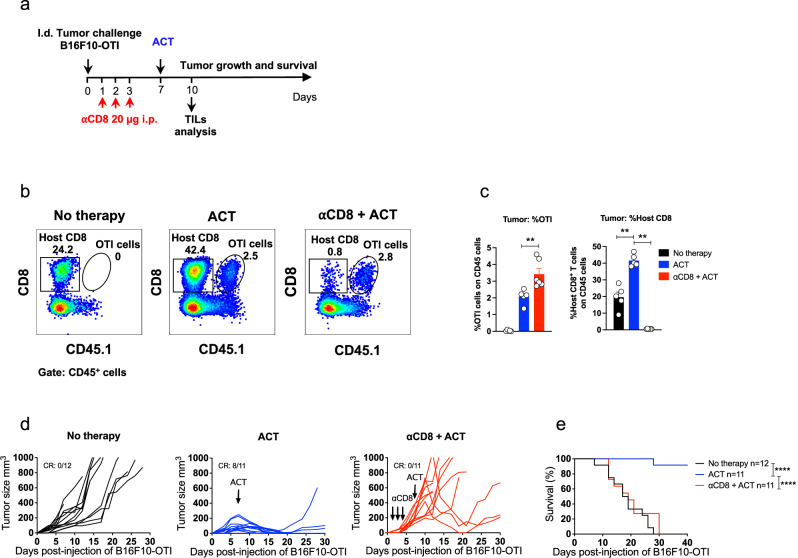
Host CD8^+^ T cells contribute to ACT-mediated eradication of primary tumors. C57BL/6 mice bearing B16F10-OTI tumors received i.v. transfer of 1 × 10^6^ in vitro activated OTI CD8^+^ T cells (ACT). Untreated mice (No therapy) were used as controls. A group of mice received three i.p. daily doses of αCD8 antibodies starting one day after tumor challenge. **a** Experimental timeline. **b**, **c** Tumor infiltrates were analyzed by flow cytometry three days after ACT. **b** Representative dot plots displaying the frequencies of host (square, CD45.1^−^) and transferred (ellipse, OTI CD45.1^+^) CD8^+^ T cells in live CD45^+^ cells for each group: No therapy, ACT, and αCD8 + ACT. **c** Quantification of transferred OTI (left panel) and host (right panel) CD8^+^ T cells as the percentage of live CD45^+^ cells for each group: No therapy (black bars), ACT (blue bars), αCD8 + ACT (red bars). Individual tumor growth (**d**) and Kaplan–Meier (**e**) curves for each group: No therapy (black curves), ACT (blue curves), and αCD8 + ACT (red curves). **c** One experiment, No therapy *n* = 5; ACT *n* = 5; αCD8 + ACT *n* = 6. Bars represent mean ± SEM. Statistical significance was assessed using a two-sided unpaired Mann–Whitney test, **P < 0.01. **e** Data from three independent experiments. Statistical significance was assessed using a two-sided log-rank Mantel–Cox test, ****P < 0.0001. CR complete response.

Next, we investigated the contribution of the host immune system to ACT-mediated tumor control using complementary approaches. First, we tested the ability of ACT to eliminate primary tumors in RAGKO mice, which lack mature T and B cells (Supplementary Fig. 2a). In these mice, tumors grew faster than in immunocompetent wild-type mice, and ACT failed to control tumor growth (Supplementary Fig 2b, c), arguing that host T and/or B cells are required for the antitumor effects of ACT. To evaluate the contribution of host T cells in immunocompetent mice, mice were treated with FTY720 (Supplementary Fig. 2d), which prevented tumor accumulation of host T cells without impairing tumor infiltration of transferred CD8^+^ T cells (Supplementary Fig. 2e, f)^41^. We observed that ACT was unable to control tumor growth in FTY720-treated mice (Supplementary Fig. 2g, h), indicating that the trafficking of host T cells from lymph nodes to tumors plays a key role in the ACT-mediated control of primary tumors. To dissect the involvement of host CD8^+^ T cells in supporting the antitumor effects of ACT, mice were treated with αCD8 depleting antibodies prior to the adoptive transfer (Fig. 2a), which efficiently eliminated host CD8^+^ T cells without affecting transferred OTI cells (Fig. 2b, c). Interestingly, despite initial tumor control in some mice, ACT failed to efficiently eliminate tumors in mice depleted of host CD8^+^ T cells, and the associated survival benefit was lost (Fig. 2d, e). We also studied the role of CD4^+^ T cells (Supplementary Fig. 3a) and found that antibody-mediated elimination of CD4^+^ T cells prior to ACT did not impair tumor rejection (Supplementary Fig. 3b, c), in fact, it resulted in reduced tumor size (Supplementary Fig. 3d), indicating that effector activity of CD4^+^ T cells is not required for effective tumor control. Taken together, these results highlight that ACT can engage the participation of host CD8^+^ T cells to provide effective control of primary tumors.

### ACT induces TNF- and cross-presenting dendritic cell-dependent expansion of host CD8^+^ T cells, resulting in effective eradication of primary tumors

**Fig. 3:**
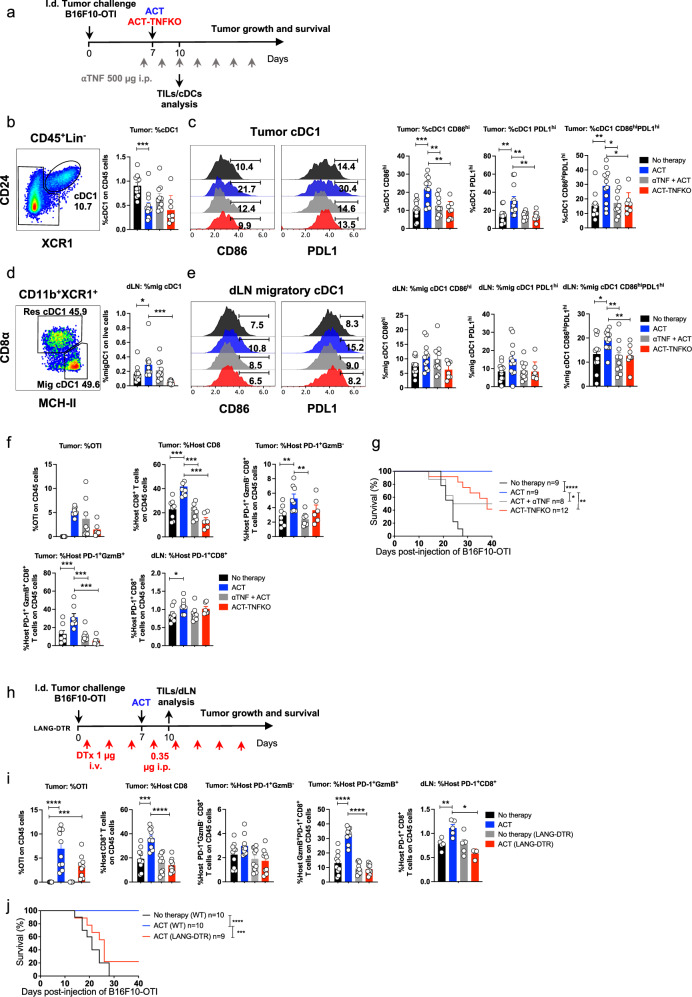
ACT induces TNF-dependent expansion of host CD8^+^ T cells and promotes cDC1 maturation and migration to draining lymph nodes. **a**–**g** C57BL/6 mice bearing B16F10-OTI tumors received i.v. transfer of 1 × 10^6^ in vitro activated OTI CD8^+^ T cells (ACT) or TNF-deficient OTI (ACT-TNFKO). One ACT group received αTNF i.p. every other day, starting one day before ACT. **a** Experimental design. Tumors (**b**, **c**) and dLN (**d**, **e**) were analyzed three days after ACT: No therapy (black), ACT (blue), ACT + αTNF (gray), and ACT-TNFKO (red). **b** Representative plots and relative quantification of cDC1 (CD24^+^XCR1^+^) within CD45^+^/Lin^−^/MHCII^+^CD11c^+^ cells. **c** CD86 and PDL1 expression and relative quantification of CD86^hi^PDL1^hi^ cDC1. **d** Representative plots and relative quantification of resident (CD8α^+^MHCII^+^) and migratory (CD8α^−^MHCII^hi^) cDC1 within CD45^+^/Lin^−^/MHCII^+^CD11c^+^/CD11b^−^XCR1^+^. **e** CD86 and PDL1 expression and relative quantification of CD86^hi^PDL1^hi^ cDC1. **f** Quantification of tumor-infiltrating transferred OTI, host CD8^+^, host PD-1^+^GzmB^−^, host PD-1^+^GzmB^+^ on CD45^+^ cells. **g** Kaplan–Meier curves. **h**–**j** C57BL/6 wild-type and Langerin-DTR tumor-bearing mice received ACT; Langerin-DTR mice received diphtheria toxin from day one post-challenge. Tumors and dLN were analyzed three days after ACT. No therapy (WT mice: black; Lang-DTR mice: gray) and ACT (WT mice: blue; Lang-DTR mice: red). **h** Experimental timeline. **i** Quantification of tumor-infiltrating transferred OTI, host CD8^+^, host PD-1^+^GzmB^−^, host PD-1^+^GzmB^+^ on CD45^+^ cells, and host PD-1^+^CD8^+^ on live cells in dLN. **j** Kaplan–Meier curves. **b**–**e** Three independent experiments; No therapy *n* = 12; ACT *n* = 12; ACT + αTNF *n* = 10; ACT-TNFKO *n* = 8. **f** Two independent experiments; No therapy *n* = 8; ACT *n* = 8; ACT + αTNF *n* = 8; ACT-TNFKO *n* = 6. Bars show mean ± SEM. *P < 0.05, **P < 0.01, ***P < 0.001 by two-sided unpaired Mann–Whitney test. (**g**) Kaplan–Meier curves from two independent experiments, analyzed by a two-sided log-rank Mantel–Cox test *P < 0.05, **P < 0.01, ****P < 0.0001. **i** Tumor: Two independent experiments, No therapy (WT *n* = 10; Lang-DTR *n* = 10) and ACT (WT *n* = 10; Lang-DTR *n* = 9). dLN: One experiment, No therapy (WT *n* = 5; Lang-DTR *n* = 4) and ACT (WT *n* = 5; Lang-DTR *n* = 4). Bars show mean ± SEM. *P < 0.05, **P < 0.01, ***P < 0.001, ****P < 0.0001 by a two-sided unpaired Mann–Whitney test. **j** Kaplan–Meier curves from two independent experiments, analyzed by a two-sided log-rank Mantel–Cox test ***P < 0.001, ****P < 0.0001.

To investigate the mechanism underlying the interplay between transferred and host CD8^+^ T cells, we examined the effects of ACT in the dendritic cell (DC) compartment and the involvement of TNF, an effector cytokine known to promote innate and adaptive antitumor immune responses^42^ (Fig. 3a). As anticipated, frequencies of tumor-infiltrating cDC1 upregulating PD-L1 and CD86 were increased following ACT, an effect that was abrogated in mice treated with a blocking αTNF antibody or that received TNF-deficient OTI CD8^+^ T cells (ACT-TNFKO) (Fig. 3b, c; Supplementary Fig. 4a–c). Consistently, ACT induced a TNF-dependent increase in the frequency of migratory cDC1 in draining lymph nodes (Fig. 3d; Supplementary Fig. 4d–f), particularly those exhibiting a CD86^high^PDL1^high^ phenotype (Fig. 3e). Together, these findings indicate ACT drives TNF-dependent activation and lymph node accumulation of cDC1, which are key events leading to effective host antitumor responses. Consequently, both TNF blockade and TNFKO ACT failed to expand host PD-1^+^ CD8^+^ T cells exhibiting tumor-reactive phenotypes (Fig. 3f) and significantly impaired primary tumor elimination and overall survival in mice (Fig. 3g and Supplementary Fig. 5). Interestingly, expansion of host PD-1^+^ CD8^+^ T cells was not significantly affected by blocking the effector cytokine IFNγ alone (Supplementary Fig. 6a, b). However, simultaneous blockade of TNF and IFNγ resulted in a synergistic reduction of host PD-1^+^GzmB^+^ CD8^+^ T cells infiltrating tumors (Supplementary Fig. 6b), suggesting that these cytokines likely engage complementary mechanisms that support the expansion and cytotoxic differentiation of CD8^+^ T cells, respectively^43,44^.

To address the participation of cDC1 in the expansion of host CD8^+^ T cells, we used the Langerin-DTR mice that express the human diphtheria toxin receptor (DTR) and the enhanced green fluorescent protein (EGFP) genes under the control of langerin (CD207) promoter^45^. In this transgenic mouse model, DTR and EGFP are expressed at different levels among different DC subsets (Supplementary Fig. 7a), allowing the depletion of tissue-migratory and lymph node-resident cDC1s, as well as Langerhans cells upon diphtheria toxin (DTx) administration (Supplementary Fig. 7b, c). In these mice, depletion of tumor cDC1 was not efficient, which likely reflects their relatively low DTR expression (Supplementary Fig. 7a) and ensures the trafficking of transferred effector T cells to tumors^46^. In Lang-DTR mice that began receiving DTx administrations one day after tumor challenge (Fig. 3h), we observed that adoptively transferred CD8^+^ T cells efficiently infiltrated tumors (Fig. 3i). However, ACT failed to promote the expansion of host CD8^+^ T cells displaying tumor-reactive phenotypes, as observed in wild-type mice (Fig. 3i). In consequence, ACT induced only a transient antitumor effect in Lang-DTR mice, with most tumors progressing and leading to decreased survival, unlike the complete protection observed in wild-type controls (Fig. 3j, Supplementary Fig. 7d). Taken together, these results demonstrate that effective ACT induces, via TNF, a cDC1-dependent expansion of host CD8^+^ T cells to achieve effective control of primary tumors.

### ACT-induced host CD8^+^ T cell immunity confers protection against ACT-resistant tumor cells lacking the target antigen

**Fig. 4:**
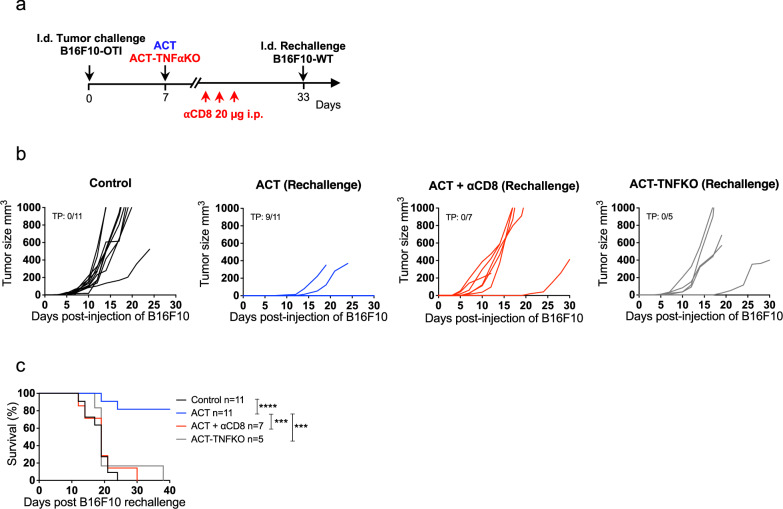
ACT induces TNF-dependent host CD8⁺ T cell immunity that rejects ACT-resistant tumor cells lacking the target antigen. C57BL/6 mice bearing B16F10-OTI tumors received i.v. transfer of 1 × 10^6^ in vitro activated wild-type (ACT) or TNFKO (ACT-TNFKO) OTI CD8^+^ T cells, then mice that rejected B16F10-OTI tumors following ACT received or did not receive three i.p. daily doses of αCD8 and five days later were i.d. rechallenged with 1 × 10^6^ of wild-type B16F10 cells in the opposite flank. **a** Experimental timeline. Individual tumor growth (**b**) and Kaplan–Meier curves (**c**) for rechallenged groups: Control (black curves), ACT (blue curves), ACT + αCD8 (red curves), and ACT-TNFKO (gray curve). **c** Kaplan–Meier curves show survival from two independent experiments. Statistical significance was assessed using a two-sided log-rank Mantel–Cox test ***P < 0.001, ****P < 0.0001. TP total protection.

Since the emergence of tumor cells that lose the expression of the targeted antigen, so-called antigen-loss variants, is a well-documented mechanism of ACT resistance that contributes to disease progression^47^, we assessed the ability of host tumor-specific CD8⁺ T cells primed following ACT to confer long-term antitumor immunity against tumors lacking the target antigen. To this end, mice that had eliminated B16F10-OTI melanoma tumors were rechallenged in the opposite flank with wild-type B16F10 cells (Fig. 4a). Notably, most of these mice that had received ACT were protected to rechallenge (Fig. 4b, c), and this protection was abolished when CD8^+^ T cells were depleted prior to rechallenge or when mice received ACT-TNFKO (Fig. 4b, c). These results indicate that effective ACT promotes TNF-dependent CD8^+^ T cell-mediated host durable immunity against other melanoma antigens, a phenomenon known as antigen spreading, which is critical to control ACT-resistant tumor cells lacking the targeted antigen. We also studied the role of CD4^+^ T cells in this context and observed that the administration of an αCD4 depleting antibody prior to ACT-mediated elimination of primary tumors failed to reject the B16F10 rechallenge (Supplementary Fig. 8a–c), underscoring the critical helper role of CD4^+^ T cells in promoting durable memory CD8^+^ T cell immunity^48^. Taken together, these results highlight that ACT engages host CD8^+^ T cells to provide durable antitumor immunity in a TNF- and CD4^+^ T cell-dependent manner.

### Lymphodepleting preconditioning enhances ACT-mediated tumor eradication but abrogates durable host antitumor immunity

**Fig. 5:**
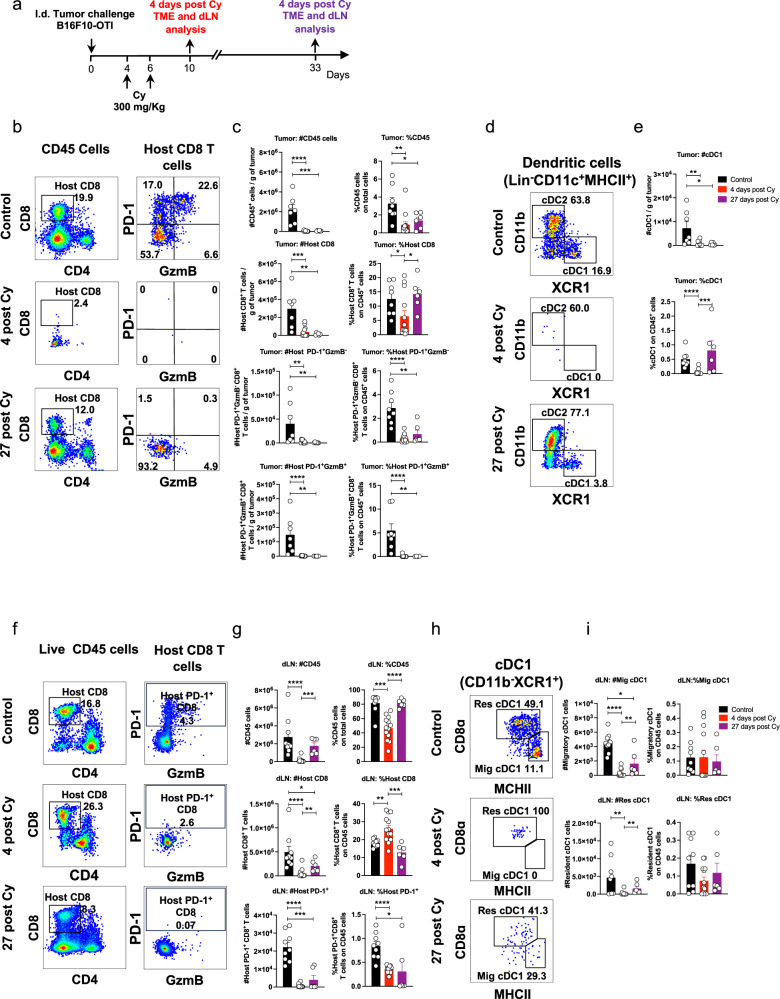
Cyclophosphamide-induced lymphodepletion results in sustained reductions in cDC1s and CD8⁺ T cell numbers in tumors and draining lymph nodes. C57BL/6 mice bearing B16F10-OTI tumors received 300 mg/kg of cyclophosphamide (Cy) at days 4 and 6 after tumor challenge. Tumors and draining lymph nodes were analyzed by flow cytometry 4 and 27 days after the last Cy dose. **a** Experimental scheme. **b**, **c** Flow cytometry analysis of tumor-infiltrating lymphocytes. **b** Representative dot plots showing CD8⁺ T cells within CD45^+^ cells (left panels) and PD-1 and GzmB expression within CD8⁺ T cells (right panels). **c** Quantifications of absolute numbers and frequencies of CD45⁺ cells, total, PD-1⁺GzmB⁻, and PD-1⁺GzmB⁺ CD8⁺ T cells. **d**, **e** Flow cytometry analysis of tumor-infiltrating dendritic cells. **d** Representative dot plots of cDC1 and cDC2 within live dendritic cells (CD45⁺Lin⁻CD11c⁺MHCII⁺). **e** Quantifications of absolute numbers and frequencies of cDC1. **f**, **g** Analysis of host CD8⁺ T cells in draining lymph nodes (dLN). **f** Representative dot plots showing host CD8⁺ T cells and their PD-1 and GzmB expression. **g** Absolute and relative quantification of CD45⁺ cells, host CD8⁺ T cells, and host PD-1⁺ CD8⁺ T cell subsets. **h**, **i** Flow cytometry analysis of dendritic cell subsets. **h** Representative dot plots of migratory (Mig) and resident (Res) cDC1 (CD11c⁺MHCII⁺XCR1⁺) populations. **i** Quantifications of absolute numbers and frequencies of migratory and resident cDC1 populations. **c**, **e**, **g**, **i** Pooled data from two independent experiments, Control *n* = 8; 4 days post Cy *n* = 13; 27 days post Cy *n* = 6. Bars represent mean ± SEM. Statistical significance was assessed using two-sided unpaired Mann–Whitney test *P < 0.05, **P < 0.01, ***P < 0.001, ****P < 0.0001.

**Fig. 6:**
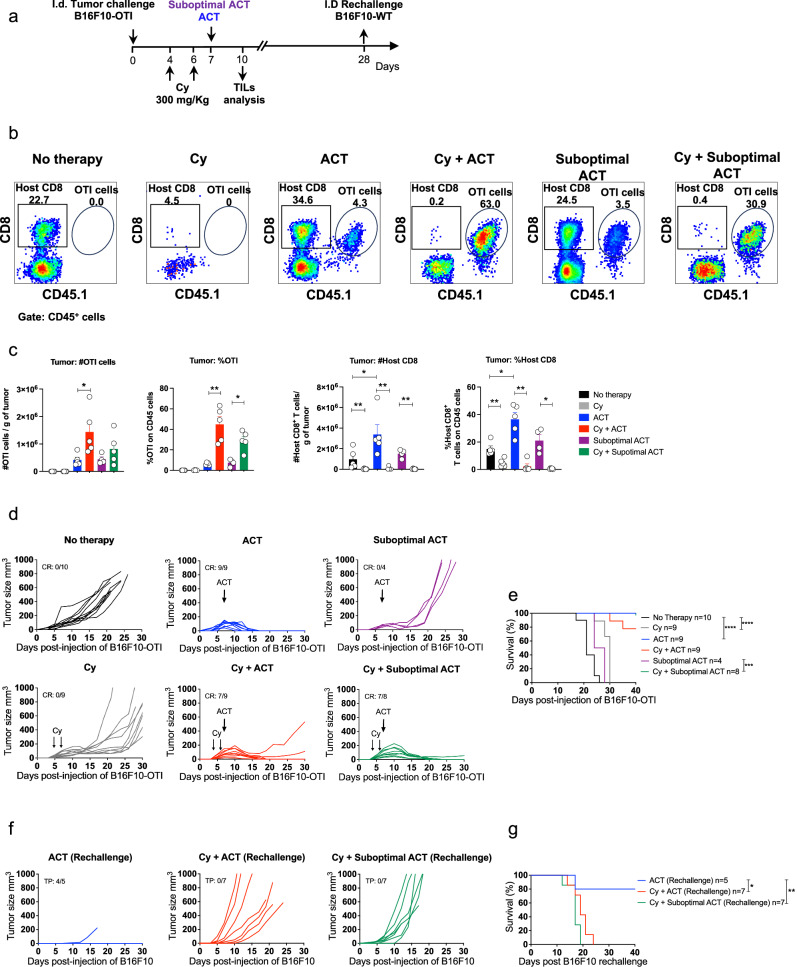
Lymphodepleting preconditioning enhances ACT-mediated tumor elimination but abrogates protection against ACT-resistant tumor cells. C57BL/6 mice bearing B16F10-OTI tumors received 300 mg/kg cyclophosphamide (Cy) i.p. on days 4 and 6 after tumor challenge as a lymphodepleting preconditioning regimen. One day later, mice received i.v. transfer of 1 × 10^6^ or 0.5 × 10^6^ in vitro activated OTI CD8^+^ T cells as optimal and suboptimal ACT models, respectively. **a** Experimental timeline. **b**, **c** Flow cytometry analysis of tumor-infiltrating lymphocytes three days after ACT. **b** Representative dot plots displaying the frequencies of host (CD45.1-) and transferred (OTI CD45.1^+^) CD8^+^ T cells in live CD45^+^ cells for each group: No therapy, Cy, ACT, Cy + ACT, Suboptimal ACT, and Cy + Suboptimal ACT. **c** Absolute (cells/g tumor) and relative (on CD45^+^ cells) quantifications of transferred OTI and host CD8^+^ T cells in the tumor for each group shown in (**b**). Individual tumor growth (**d**) and Kaplan–Meier (**e**) curves for each group: No therapy (black curves), ACT (blue curves), and Suboptimal ACT (purple curves), Cy (gray curves), Cy + ACT (red curves), and Cy + Suboptimal ACT (green curves). **f**, **g** Mice that rejected tumors were rechallenged with 1 × 10^6^ wild-type B16F10 in the opposite flank. Individual tumor growth (**f**) and Kaplan–Meier (**g**) curves of rechallenged mice for each group: ACT (blue curves), Cy + ACT (red curves), and Cy + suboptimal ACT (green curves). **c** One experiment, No therapy *n* = 5; Cy *n* = 5; Cy + ACT *n* = 5; Suboptimal ACT *n* = 5; Cy + Suboptimal ACT *n* = 5. Bars represent mean ± SEM. Statistical significance was assessed using a two-sided unpaired Mann–Whitney test *P < 0.05, **P < 0.01. Kaplan–Meier curves showing the survival from two independent experiments (**e**), and from one experiment (**g**). Statistical significance was assessed using two-sided log-rank Mantel–Cox test *P ˂ 0.05, **P ˂ 0.01, ***P < 0.001, and ****P < 0.0001. CR complete response, TP total protection.

Next, we studied how lymphodepleting preconditioning, which is a component part of most ACT protocols in the clinic, would impact long-term host antitumor immunity in our model. To this end, we analyzed the changes in different immune cells, focusing on CD8^+^ T cell and DC populations, in tumors and draining lymph nodes at early (day 4) and late (day 27) timepoints after cyclophosphamide treatment^49^ (Fig. 5a). As expected, flow cytometry analysis revealed a profound reduction across the different CD45⁺ immune cell populations in tumors analyzed at day 4 (Fig. 5b–e and Supplementary Fig. 9a, b). At 27 days post-cyclophosphamide treatment, tumors exhibited a sustained reduction of CD45^+^ immune cell populations (Fig. 5b–e and Supplementary Fig. 9a, b), including CD8^+^ T cell (Fig. 5b, c) and cDC1 (Fig. 5d, e) populations, features characteristic of “cold” tumors. Notably, cyclophosphamide treatment drastically reduced the frequencies of PD-1^+^CD8⁺ T cell subsets (Fig. 5b, c), suggesting long-lasting impairment of T cell priming. In lymph nodes, cyclophosphamide treatment led to a reduction in all immune populations by day 4, most of which returned to baseline levels by day 27 (Fig. 5f–i and Supplementary Fig. 9c, d), except for CD8⁺ T cells (Fig. 5f, g) and cDC1s (Fig. 5h, i), which were only partially restored. Despite partial recovery of host CD8⁺ T cells, frequencies of PD-1^+^CD8⁺ T cells were nearly undetectable in most mice (Fig. 5g), further supporting the notion of sustained impairment in T cell priming. These results indicate that lymphodepleting preconditioning induces a prolonged impairment of host antitumor immunity that may compromise long-term protection of ACT. To address this, we assessed the impact of cyclophosphamide preconditioning on host and transferred cells and their ability to eliminate primary tumors and reject ACT-resistant melanoma cells (Fig. 6a). Despite depleting host CD8⁺ T cells, cyclophosphamide promoted the expansion of transferred OTI CD8⁺ T cells in both optimal and suboptimal ACT models (Fig. 6b, c), leading to complete tumor control in the suboptimal setting (Fig. 6d, e). These results confirm that lymphodepleting preconditioning promotes the expansion of transferred cells and effective control of primary tumors in our model, consistent with clinical studies^49,50^.

To evaluate the impact of lymphodepleting preconditioning on protection against ACT-resistant melanoma cells, mice that had eliminated primary tumors were rechallenged with B16F10 cells. Notably, all mice treated with cyclophosphamide failed to reject the B16F10 rechallenge (Fig. 6f, g), whereas their ability to reject B16F10-OTI cells was preserved (Supplementary Fig. 10a–e), indicating lymphodepleting preconditioning has a detrimental effect specifically on host antitumor immunity. Furthermore, we validated these results using cyclophosphamide at low dose, aiming to better preserve the host immune system, and observed that long-term protection against ACT-resistant tumor cells was also abrogated (Supplementary Fig. 11a–e), reinforcing that even mild lymphodepleting preconditioning compromises host long-term antitumor immunity. To validate these findings in an independent model, we engineered the MC38 colorectal tumor cells to express the immunodominant epitope of the antigen GP100 (GP100_25-33_), also known as PMEL, which can be recognized by CD8^+^ T cells from the TCR-transgenic mouse model PMEL-1^51,52^. This cell line enabled us to simultaneously test a different and widely used tumor model and a clinically relevant antigen/TCR^53,54^. Similar to the melanoma model, ACT alone or in combination with cyclophosphamide effectively eliminated established MC38-PMEL tumors, with complete responses observed in all treated mice (Supplementary Fig. 12a–c). However, upon rechallenge with ACT-resistant wild-type MC38 tumor cells, only mice that received ACT in the absence of cyclophosphamide were able to mediate tumor rejection, whereas preconditioned mice failed to sustain long-term protective immunity (Supplementary Fig. 12d, e). Consistently, CD8^+^ T cell depletion prior to rechallenge also abrogated tumor rejection in ACT responders, confirming the essential role of host CD8⁺ T cells in mediating long-term tumor control (Supplementary Fig 12d, e). Altogether, these findings, confirmed in two independent tumor models, reveal an underappreciated role of lymphodepleting preconditioning beyond its ability to promote the expansion of transferred cells; it undermines ACT-induced durable antitumor immunity capable of controlling antigen-loss variants, eventually promoting resistance to ACT in mouse models.

### cDC1, TNF signaling, Tpex, and Tex profiles positively associate with favorable clinical responses to ACT and overall survival in human melanoma

**Fig. 7:**
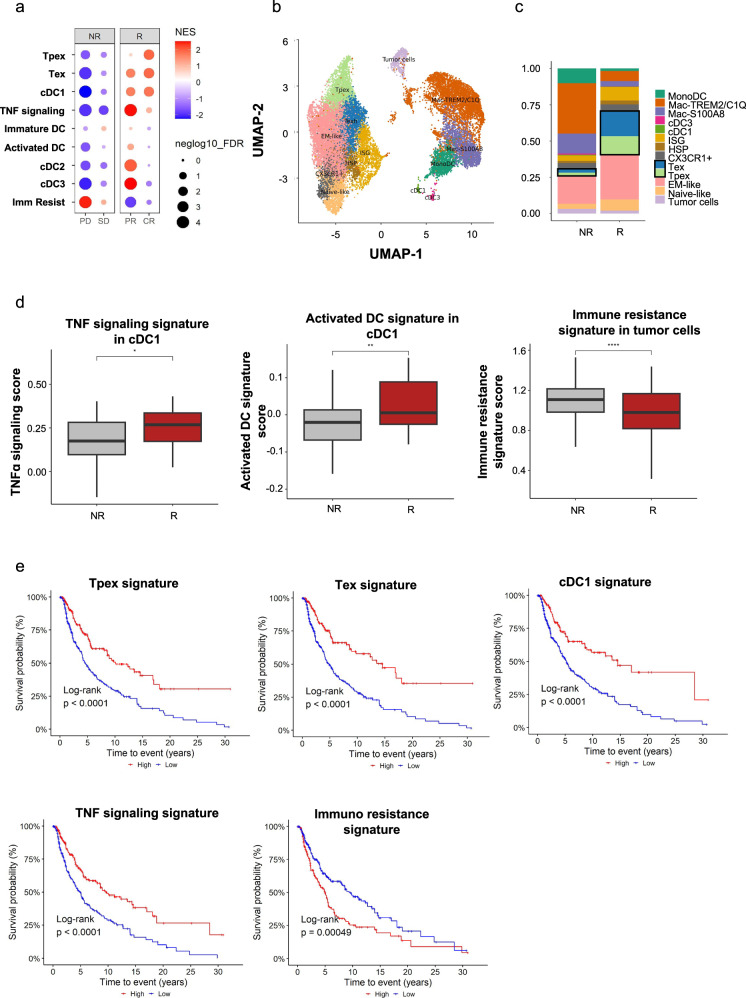
cDC1, TNF signaling, and Tpex and Tex transcriptomic profiles are positively associated with clinical responses to ACT and overall survival in melanoma patients. **a** Gene Set Enrichment Analysis (GSEA) of bulk RNA-seq from melanoma patients before ACT, stratified by the Response Evaluation Criteria in Solid Tumors (RECIST) score: progressive disease (PD, *n* = 5), stable disease (SD, *n* = 10), partial response (PR, *n* = 5), and complete response (CR, *n* = 5). Normalized enrichment scores (NES) and adjusted *p*-values (FDR, −log10 scale) for each signature were calculated by comparing each RECIST group against the others. The weighted method was used for enrichment statistic, and the Signal2Noise method for ranking genes. **b**, **c** Single-cell RNA-seq analysis of melanoma tumors from patients before TIL therapy. **b** UMAP plot showing major immune and tumor cell clusters. **c** Stacked bar graph showing the proportional distribution of annotated clusters between non-responders (NR) and responders (R) patients, with highlighted Tpex and Tex populations. **d** Gene signature scores derived from scRNA-seq data for TNF signaling and activation on cDC1 and immune resistance in tumor cells, stratified by response to ACT. **e** Kaplan–Meier survival analysis of TCGA-SKCM melanoma patients (*n* = 460) stratified by high (red) versus low (blue) expression of levels of selected gene signatures (Tpex, Tex, cDC1, TNF signaling, and immune resistance). **d** Boxplots depict the median (center line), interquartile range (25th–75th percentiles), and whiskers extending to values within 1.5× the interquartile range. Statistical significance was assessed using a two-sided *t*-test: *P < 0.05, **P < 0.01, and ****P < 0.0001. **e** Signature levels were binarized using the maximization method. Statistical significance was assessed using a two-sided log-rank test. Mantel–Cox *P* < 0.05 was considered significant.

To investigate whether active host antitumor T cell immunity is associated with clinical responses to ACT in clinical settings, we analyzed publicly available datasets of melanoma patients receiving TILs for which tumor bulk RNA-seq and clinical response (RECIST) data were available^55^. Consistent with our findings in mouse models, gene signatures associated with Tpex, Tex, cDC1 and TNF signaling (Supplementary Data 1) were enriched in tumors of patients showing favorable clinical responses to ACT (responders, R), including complete responses (CR) and partial responses (PR) (Fig. 7a). In contrast, these signatures were downregulated in patients with progressive disease (PD) and stable disease (SD) (non-responders, NR). Notably, Tpex, Tex, and cDC1 signatures were specifically associated with complete responses, underscoring their critical role in supporting ACT efficacy. The TNF-signaling signature was strongly associated with partial responses, but only weakly associated with complete responses, probably reflecting a more complex signaling network involved in antitumor protection. Signatures of other DC subsets, cDC2 and cDC3, were enriched exclusively in PR patients and not in those achieving CR, while generic signatures of activated (acDC) or immature dendritic cells (immDC) showed no significant enrichment in responders (Fig. 7a). Additionally, an immune gene signature previously associated with immunotherapy resistance in melanoma (ImmuneRes)^56^ was enriched in NR patients and downregulated in R patients, further supporting its potential role in predicting treatment outcomes.

To enable in-depth analysis of the most relevant populations, we integrated data exclusively from CD8^+^ T cells, myeloid cells, and tumor cells derived from responding and non-responding patients. Differential expression analysis revealed the presence of heterogeneous myeloid and lymphoid populations (Fig. 7b and Supplementary Fig. 13). Among CD8^+^ T cells, we found seven distinct subsets, including Tpex and Tex populations, along with other subsets expressing PD-1 and/or GzmB (effector memory-like (EM-like), CX3CR1⁺, naïve-like, heat shock protein (HSP)-expressing, and interferon-stimulated gene (ISG)-enriched). As expected, both Tpex and Tex populations were expanded in responder patients (Fig. 7c), confirming results observed in bulk RNA-seq analyses. In addition, five myeloid clusters were identified: cDC1, cDC3, MonoDC, S100A8⁺ macrophages, and TREM2⁺C1Q⁺ macrophages. We observed that enrichment of both TNF-signaling and activation gene signatures in cDC1 was also associated with positive responses to ACT (Fig. 7d). As a control, the immune resistance gene signature in tumor cells was enriched in non-responding patients. In summary, these findings support a model in which ongoing antitumor immunity characterized by CD8⁺ T cell expansion and TNF-driven activation of cDC1 contributes to effective responses to ACT. Finally, we investigated whether Tpex, Tex, cDC1, and TNF-signaling gene signatures were associated with better survival in a larger cohort of patients with cutaneous skin melanoma (SKCM) from The Cancer Genome Atlas (TCGA) database. As we expected, higher expression levels of all these signatures were associated with longer survival. In contrast, high expression of the immune resistance signature showed a negative association with patient survival (Fig. 7e). Although further evidence needs to be obtained from clinical studies, these results support our findings in mouse models and suggest that TNF signaling, cDC1 activation and expansion of tumor-reactive CD8^+^ T cells are critical for the efficacy of ACT and for the survival of patients with melanoma.

## Discussion

Despite significant advances in the clinical implementation of ACT as a frontier medicine for cancer patients, the mechanisms underlying durable antitumor immunity, as well as the factors contributing to acquired resistance in solid tumors, are not fully understood^57^. In this work, we demonstrate that durable ACT efficacy against solid tumors relies not only on the antitumor activity of transferred T cells, but also on their ability to engage host CD8^+^ T cells, via TNF and a cDC1-dependent mechanism, that contribute to primary tumor elimination and protect against ACT-resistant antigen-negative tumor cells. Our findings extend prior observations demonstrating the importance of the host immune system in ACT responses. Previous studies have shown that transferred T cells can stimulate host immune cells, primarily through IFNγ, supporting the cytotoxic potential of both transferred and host T cells and promoting effective tumor control in preclinical models^37,38^. Our work provides mechanistic insights, revealing that TNF produced by transferred CD8^+^ T cells plays a critical role in mediating the crosstalk with host CD8^+^ T cells. We show that TNF from transferred CD8⁺ T cells promotes maturation and migration to lymph nodes of cDC1, which induce the expansion of host CD8^+^ T cells and durable tumor protection. This mechanism mirrors the role described for CD8⁺ T cell–derived TNF in driving maturation and lymph node migration of DC in the context of viral infections^5^. Supporting its clinical relevance, a recent study showed that both the ability of infused CD8^+^ T cells to produce TNF and elevated host serum levels of Flt3-L, a factor known to drive cDC1 differentiation from hematopoietic progenitors, are associated with better clinical responses to TCR-based ACT in metastatic melanoma patients^3,58,59^. We also observed that melanoma patients treated with TIL therapy whose tumors showed enrichment for Tpex, Tex, TNF signaling, and cDC1 gene signatures exhibited a positive correlation with therapeutic response to ACT and overall survival. The beneficial role of tumor-reactive CD8^+^ T cell populations and cDC1-restricted TNF signaling in clinical responses to ACT was confirmed in an independent cohort of patients with single-cell resolution data. Consistent with our findings, a recent study showed that, in recurrent large B-cell lymphoma patients treated with CAR-T cells that did not persist long-term, durable remissions are associated with the expansion of host T cells exhibiting cytotoxic, proliferative, and proinflammatory profiles, as compared to patients who relapsed to ACT^60^. Together, these observations uncover a mechanism that highlights TNF as a key effector cytokine orchestrating a cDC1-dependent interplay between transferred CD8^+^ T cells and host antitumor immunity, driving durable ACT clinical outcomes^35,36^.

Importantly, our work also reveals an underappreciated trade-off of lymphodepleting preconditioning: on one hand, it enhances acute ACT efficacy by promoting transferred T cell expansion; on the other hand, it impairs host antitumor immunity capable of controlling antigen-loss variants in mouse models. It will be important to evaluate whether lymphodepleting preconditioning promotes resistance to ACT in clinical settings. Cyclophosphamide preconditioning induced a long-lasting reduction in immune infiltration of tumors, along with the selective decrease of host CD8⁺ T cells and cDC1s in lymph nodes, resulting in impaired CD8⁺ T cell priming capacity. These results suggest that lymphodepleting preconditioning may convert tumors immunologically ‘hot’ to ‘cold’, potentially rendering them unresponsive to subsequent ACT reinfusions or other immunotherapies. In addition, preconditioning regimens induce substantial toxicities, including pan-cytopenia, immune suppression, and reactive myelopoiesis, which are not necessarily associated with better clinical responses^61^. These findings challenge the universal benefit of lymphodepletion and advocate for the development of selective conditioning strategies that spare host CD8^+^ T cells and cDC1, while eliminating other suppressive populations (e.g., Treg cells and myeloid-derived suppressor cells). Alternatively, cell-autonomous strategies that ensure effective engraftment of transferred cells in the absence of lymphodepleting preconditioning need to be developed^62^. In line with this, a phase 1 study of neoantigen-specific CD8⁺ T cell therapy in advanced solid tumors showed better clinical responses in patients who did not receive lymphodepletion, suggesting ACT without preconditioning may be a viable and effective option^63^. These findings provide a mechanistic rationale for designing next-generation ACT protocols aiming not only to enhance initial tumor regression but also to maintain long-term tumor control and prevent relapse, particularly against heterogeneous tumors bearing antigen-loss variants^27–29^

## Methods

### Study design

The overall objective of the study was to test the hypothesis that host CD8^+^ T cells play a key role in ACT-mediated tumor eradication. Using different mouse models, we evaluated the requirement of host T cells for tumor elimination induced by the adoptive transfer of in vitro-activated OTI CD8^+^ T cells. The frequencies and phenotype of host and adoptively transferred tumor-infiltrating T cell populations were analyzed by flow cytometry. Sample size was determined via a priori power analysis based on means and SDs estimated from pilot experiments and previous experience. All mice were randomized before treatment initiation.

### Animals

C57BL/6/J wild-type (CD45.2), B6.129S7-*Rag1*^*tm1Mom*^/J (RAG1KO), C57BL/6-Tg(TcraTcrb)1100Mjb/J (OTI), CBy.SJL(B6)-*Ptprc*^*a*^*/J* (CD45.1), B6.129S2-Cd207^tm3(DTR/GFP)Mal^/J (Langerin-DTR), B6.Cg-Thy1a/Cy Tg(TcraTcrb)8Rest/J (PMEL-1), B6.129S-Tnftm1Gkl/J (TNFKO) mice were purchased from Jackson Laboratories. Mice were kept at the animal facility of Fundación Ciencia & Vida and were housed under specific pathogen-free conditions on a 12-h light/12-h dark cycle at 20–24 °C with 40–60% humidity, according to the “Guide to Care and Use of Experimental Animals, Canadian Council on Animal Care”. This study was carried out in accordance with the recommendations of the “Guidelines for the Welfare and Use of Animals in Cancer Research, Committee of the National Cancer Research Institute”. All procedures complied with all relevant ethical regulations for animal research and were approved by the “Comité de Bioética y Bioseguridad” of Fundación Ciencia & Vida. No animals were excluded from the analysis, and male and female mice were used indistinctly. Mice were allocated randomly to the different experimental procedures, and the evaluation of tumor volume was blinded when possible.

### Cell lines

Mouse melanoma cell line B16F10 (ATCC CLR-6475) was obtained from the American Type Culture Collection. MC38 tumor cells were kindly provided by Dr. Sergio A. Quezada (University College London Cancer Institute, UK). B16F10-OTIx5-ZsGreen (B16F10-OTI) and MC38-OTI.hPMELx5-ZsGreen (MC38-PMEL) cells were generated by lentiviral transduction with the pLVX-OTIx5-ZsGreen vector encoding the OTI and PMEL epitopes minigene fused to ZsGreen^39^. B16F10 and MC38 cell lines were cultured in complete RPMI 1640 (Thermo Fisher Scientific, ref 61870-036) and DMEM (HyClone, ref SH30081.02) media, respectively, supplemented with penicillin, streptomycin (Thermo Fisher Scientific, ref 15140122), non-essential amino acids (Thermo Fisher Scientific, ref 11140050), sodium pyruvate (Thermo Fisher Scientific, ref 11360070), and 10% of heat-inactivated fetal bovine serum (Thermo Fisher Scientific, ref 10437010) in a humidified incubator at 37 °C with 5% CO2. All cell lines were routinely tested for mycoplasma contamination.

### Tumor challenge and rechallenge

Mice were injected intradermally in the lower flank with 50 μL of PBS containing 1 × 10^6^ of tumor cells. Tumor rechallenge was injected in the opposite flank. Tumor growth was monitored by measuring perpendicular tumor diameters with calipers. Tumor volume was calculated using the following formula: *V* = (*D* × *d*2)/2, where *V* is the volume (mm^3^), *D* is the larger diameter (mm), and *d* is the smaller diameter (mm). Mice were sacrificed when moribund or when the mean tumor diameter was ≥15 mm, according to the approved ethical protocol.

### Adoptive cell therapy

To generate OTI or PMEL-1 TCR-transgenic CD8^+^ T cells for ACT, splenocytes from OTI or PMEL-1 mice were cultured in RPMI 1640 supplemented media containing 2 μg/mL OTI (SIINFEKL) or PMEL (KVPRNQDWL) peptides, respectively, 100 UI/mL of recombinant human IL-2 (rhIL-2; Biolegend, ref 589108), and 50 μM of 2-mercaptoethanol (Merck, ref M3148) and expanded daily for 96 h. Seven days after tumor challenge, when tumors reached a size of ~50–200 mm^3^, mice were intravenously injected with 1 × 10^6^ (optimal) or 0.5 × 10^6^ (suboptimal) activated OTI CD8^+^ T cells in 100 μL of sterile PBS (Thermo Fisher Scientific, ref 10010023).

### FTY720 treatment, antibody administration, lymphodepleting preconditioning regimen with cyclophosphamide, and DC depletion

To block recirculation of T cells, 25 μg of FTY720 (Sigma-Aldrich, ref SML0700) was injected intraperitoneally every three days starting one day after the tumor challenge. To deplete CD8⁺ or CD4⁺ T cells one day after tumor challenge, mice were intraperitoneally injected with three consecutive daily doses of 20 µg anti-CD8α monoclonal antibody (rat IgG2b, clone YTS169.4; Bio X Cell, BE0117) or 100 µg anti-CD4 monoclonal antibody (rat IgG2b, clone GK1.5; Bio X Cell, BE0003-1), respectively. Lymphodepleting preconditioning regimen prior to ACT was performed by two intraperitoneal doses of 300 or 60 (low dose) mg/kg cyclophosphamide (Sigma-Aldrich, ref. PHR1404) on days 4 and 6 after tumor challenge. Blockade of TNF and IFNγ was performed by intraperitoneal injection of 500 μg of αTNF (Bio X Cell, clone XT3.11, Ref BE0058) or αIFNγ (Bio X Cell, clone XMG1.2, Ref BE0055), administered every other day starting one day prior to ACT. To deplete cDC1s, Langerin-DTR mice received 1 μg of diphtheria toxin (Sigma-Aldrich, ref D0564 1MG) by intravenous injection in the tail vein three days after the tumor challenge and continuously maintained doses of 0.35 μg intraperitoneally every 3 days.

### Preparation of tissue cell suspensions

Tumors, inguinal lymph nodes, and skin samples were excised, cut into small fragments, and mechanically disaggregated. Samples were resuspended in 1 mL RPMI 1640 medium (Thermo Fisher Scientific, ref 61870-036) containing 5 mg/mL of collagenase type IV (Gibco, ref 17104019) and 5 μg/mL of DNAse I (AppliChem, ref A3778,0010) and incubated for 60 (tumors) or 30 (lymph nodes and skin) min at 37 °C with shaking. Samples were then resuspended in 1 mL of supplemented RPMI 1640 medium (Thermo Fisher Scientific, ref 61870-036) containing 5 μg/mL of DNase I (AppliChem, ref A3778,0010) and incubated for 5 min at 4 °C. Skin pieces were mechanically disaggregated using microscope slides with ground edges (Sail Brand, ref 7105). Single-cell suspensions were obtained using a 70 μm cell strainer (BD Falcon, ref 352350). For the analysis of tumor DCs, CD45 magnetic positive selection (MACS, Miltenyi ref 130-052-301) was used to enrich hematopoietic cells.

### Surface, intracellular, and intranuclear staining for flow cytometry

Single-cell suspensions were incubated for 10 min with the TruStain fcX (Biolegend, clone 93, ref 101320). For surface staining, cell suspensions were incubated with the antibodies for 20 min at 4 °C, followed by two washes with PBS 1×. Cells were then fixed and permeabilized for intracellular and intranuclear staining using the eBioscience FOXP3/transcription factor staining kit (Invitrogen, ref 00-5523-00), followed by intranuclear staining. Monoclonal antibodies specific for mouse molecules were purchased from Biolegend: CD45-FITC (clone 30-F11), TNF-α-FITC (MP6-XT22), CD279/PD-1-PE (clone 29f.1a12), IFNγ-PE (XMG1.2), CD64-PEDazzle (X54-5/7.1), CD8α-PerCP (clone 53-7.6), PerCP-CD3 (17A2), MHCII-PerCP (m5/114.15.2), B220-PerCP (RA3-6B2), B220-PECy5. CD45.1-PE/Cy7 (clone A20), CD366/TIM3-PE/Cy7 (clone RMT3-23), PDL1-PE/Cy7 (10F.9G2), CD45.2-APC/Cy7 (clone 104), Ly6G-APC/Fire810 (1A8), MHCII-APC/Cy7 (m5/114.15.2), CD3-Brillant Violet 421 (17A2), CD45.1-Brillant Violet 421 (clone A20), CD279/PD-1-Brillant Violet 421 (29f.1a12), CD24-Brillant Violet 421 (M1/69), CD366/TIM3-Brillant Violet 605 (RMT3-23), Ly6C-Brillant Violet 605 (HK1.4), CD8-Brillant Violet 650 (53-6.7), XCR1-Brillant Violet 650 (ZET), CD11c-Brillant Violet 711 (N418), CD11b-Brillant Violet 785 (M1/70). BD Biosciences: CD86-PE (GL1). Cell signaling technology: TCF-1/TCF7-AF488 (C63D9). Miltenyi Biotec: TOX-APC (REA473) and Invitrogen: CD45-BUV395 (30-F11), F4/80-BUV563 (BM8), CD4-BUV661 (GK1.5), CD3-BUV737 (17A2), CD11c-BUV805 (N418), Granzyme B-APC (clone GB11), Granzyme B-PE-TexasRed (clone GB11). Viability dye was made with ZombieAqua (Biolegend ref 423101). Samples were acquired in a FACSymphony A1 cytometer, FACSAria III (BD Biosciences), or Aurora (Cytek Biosciences), and data were analyzed using FlowJo version 10.8.1 (Tree Star, Inc.).

### Gene Set Enrichment Analysis

To explore whether gene signatures correlate with clinical response in patients under ACT, we performed a Gene Set Enrichment Analysis (GSEA). Normalized gene expression matrix and clinical data were downloaded from the Gene Expression Omnibus (accession number GSE100797)^55^. Patients were divided according to RECIST criteria: complete response (CR), partial response (PR), stable disease (SD), and progressive disease (PD). Signatures were obtained and manually curated from previously reported single-cell RNA-seq data and signature databases^11,56,58,59,64,65^. For enrichment analysis, we used the GSEA software (Broad Institute, v4.2.3), with 1000 permutations, using gene set as the permutation type^66^. Plot was generated with ggplot2 (v3.4).

### Single-cell RNA-sequencing (scRNA-seq) data analysis of TIL-ACT-treated melanoma patients

Single-cell RNA-seq data from TIL-ACT-treated melanoma patients were obtained from the Gene Expression Omnibus (GEO; accession number GSE222448)^67^. The dataset includes tumor samples collected before and after tumor-infiltrating lymphocyte adoptive cell therapy (TIL-ACT). For our analysis, only pre-treatment samples were used. Raw count matrices were downloaded and processed in R (v4.4.3) using Seurat (v5.2.1). The data were normalized, and the 2000 most variable genes were identified. Data were integrated and batch-corrected using canonical correlation analysis (CCA), followed by dimensionality reduction using the first 75 principal components (PCs). Major cell types were identified, and CD4⁺ T cells and B cells were excluded. CD8⁺ T cells, tumor cells, and myeloid cells were subsetted and independently evaluated to remove contaminating populations. Then, a second round of integration was performed using only CD8⁺ T cells, myeloid cells, and tumor cells. Clustering was based on the first 75 PCs using the Leiden algorithm, and resolution parameters were explored using the clustree package (v0.5.1). Clusters were annotated based on marker genes and gene signatures reported in the original study. Module scores for gene signatures were computed using the AddModuleScore function. For statistical comparisons between responders and non-responders, an unpaired two-sided *t*-test was used.

### TCGA survival analysis

To determine whether gene signatures correlate with patients’ survival, we analyzed the Cancer Genome Atlas – Skin Cutaneous Melanoma cohort. Expression and clinical data were downloaded from the Xena Browser (UCSC)^68^. For each signature, we calculated the mean log_2_(FPKM-uq + 1). Patients with inconsistent expression and clinical data were filtered. Survival analysis was performed with the “survival” package (v3.4). Patients were divided into high and low according to the gene signature optimal cut point determined with the maximally selected rank statistics method. Kaplan–Meier survival curves were drawn with the “survminer” package (v3.2). All analyses were carried out in the R environment (v4.1.1). A *P* < 0.05 was considered statistically significant.

### Statistical analysis

Statistical analyses were performed using GraphPad Prism software (version 10.1.9). The unit of study was an individual mouse independently assigned to each experimental condition. Each data point represents one biological replicate (one mouse). No technical replicates were used for statistical analysis. Mice were randomly assigned to experimental groups prior to treatment initiation; no stratification was applied. Investigators were not blinded during allocation or analysis due to the objective nature of flow cytometry-based quantifications. Unless otherwise indicated, data shown represent pooled biological replicates from independent experiments, as specified in each figure legend. For selected experiments, including depletion studies with robust and reproducible phenotypes, data are derived from a single independent experiment with the indicated number of mice per group. Comparison between relevant groups was performed using a two-sided unpaired Mann–Whitney test. Survival analysis was performed using a Kaplan–Meier curve with a two-sided log-rank Mantel–Cox test. No adjustments for multiple comparisons were applied unless otherwise indicated. Overall, *P* < 0.05 was considered statistically significant.

### Reporting summary

Further information on research design is available in the Nature Portfolio Reporting Summary linked to this article.

## Supplementary information

Supplementary Information

Description of Additional Supplementary Files

Supp Data 1

Reporting Summary

Transparent Peer Review file

## Source data

Source Data

## Data Availability

The data that support the findings of this study are available in Zenodo at 10.5281/zenodo.18661317. RNA-sequencing data from TIL-ACT-treated melanoma patients are available in the Gene Expression Omnibus (GEO) under accession numbers GSE222448 (single-cell RNA-seq; https://www.ncbi.nlm.nih.gov/geo/query/acc.cgi?acc=GSE222448) and GSE100797 (bulk RNA-seq; https://www.ncbi.nlm.nih.gov/geo/query/acc.cgi?acc=GSE100797). All data are publicly available without restriction. Source data are provided with this paper.

## Code availability

Custom R scripts are publicly available in a GitHub repository. https://github.com/ahernandezoliv/Figueroa_etal_2026.
