# Supplementary material for: Lymphodepleting preconditioning impairs host antitumor immunity induced by adoptive T cell therapy in mouse models
Source: Nat Commun. 2026 Mar 31;17:4337. doi: 10.1038/s41467-026-71082-y (PMC13172322; doi:10.1038/s41467-026-71082-y)
Supplement: Supplementary file 1 — Supplementary Information [file 41467_2026_71082_MOESM1_ESM.pdf]

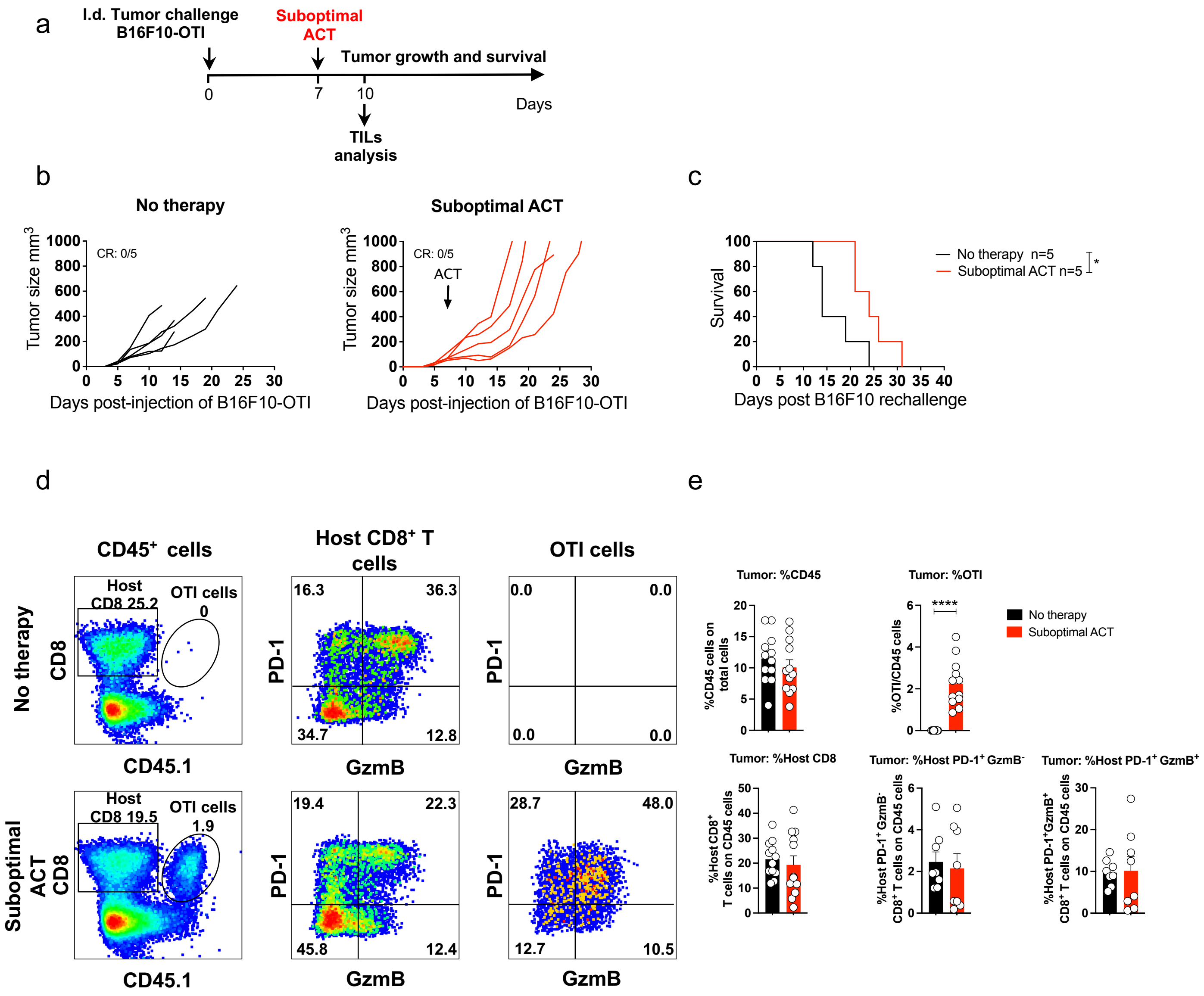

Supplementary Figure 1. Suboptimal ACT does not promote tumor accumulation of host CD8$^+$ T cells, leading to tumor progression. C57BL/6 mice bearing B16F10-OTI tumors received i.v. transfer of 0.5x10^6 *in vitro* activated OTI CD8$^+$ T cells as a Suboptimal ACT. Untreated mice (No therapy) were used as controls. **a** Experimental scheme. **b-c** Individual tumor growth (**b**) and Kaplan-Meier (**c**) curves for each group: No therapy (black curves) and Suboptimal ACT (red curves). **d-e** Tumor infiltrates were analyzed by flow cytometry three days after ACT. **d** Left panels: Representative dot plots displaying the frequencies of host (square, CD45.1$^-$) and transferred (ellipse, OTI CD45.1$^+$) CD8$^+$ T cells in live CD45$^+$ cells. Middle and right panels: Representative dot plots displaying the expression of PD-1 and granzyme B in host (middle) and transferred (right) CD8$^+$ T cells and the frequencies of the different subpopulations defined in each quadrant: PD-1$^+$GzmB$^-$, PD-1$^+$GzmB$^+$, PD-1$^-$GzmB$^-$ and PD-1$^-$GzmB$^+$. **e** Quantifications of CD45$^+$ cells as a percentage of total live cells, transferred OTI, total host CD8$^+$ T cells and their PD-1$^+$ GzmB$^-$ and PD-1$^+$ GzmB$^+$ subsets as a percentage of CD45$^+$ cells. (**c**) Kaplan–Meier curves show survival from two independent experiments. Statistical significance was assessed using two-sided log-rank Mantel-Cox test *P<0.05. (**e**) Pooled data from two independent experiments, No therapy n=12; Suboptimal ACT n=12. Bars represent mean ± SEM. Statistical significance was assessed using two-sided unpaired Mann-Whitney test ****P<0.0001. CR = Complete Response.

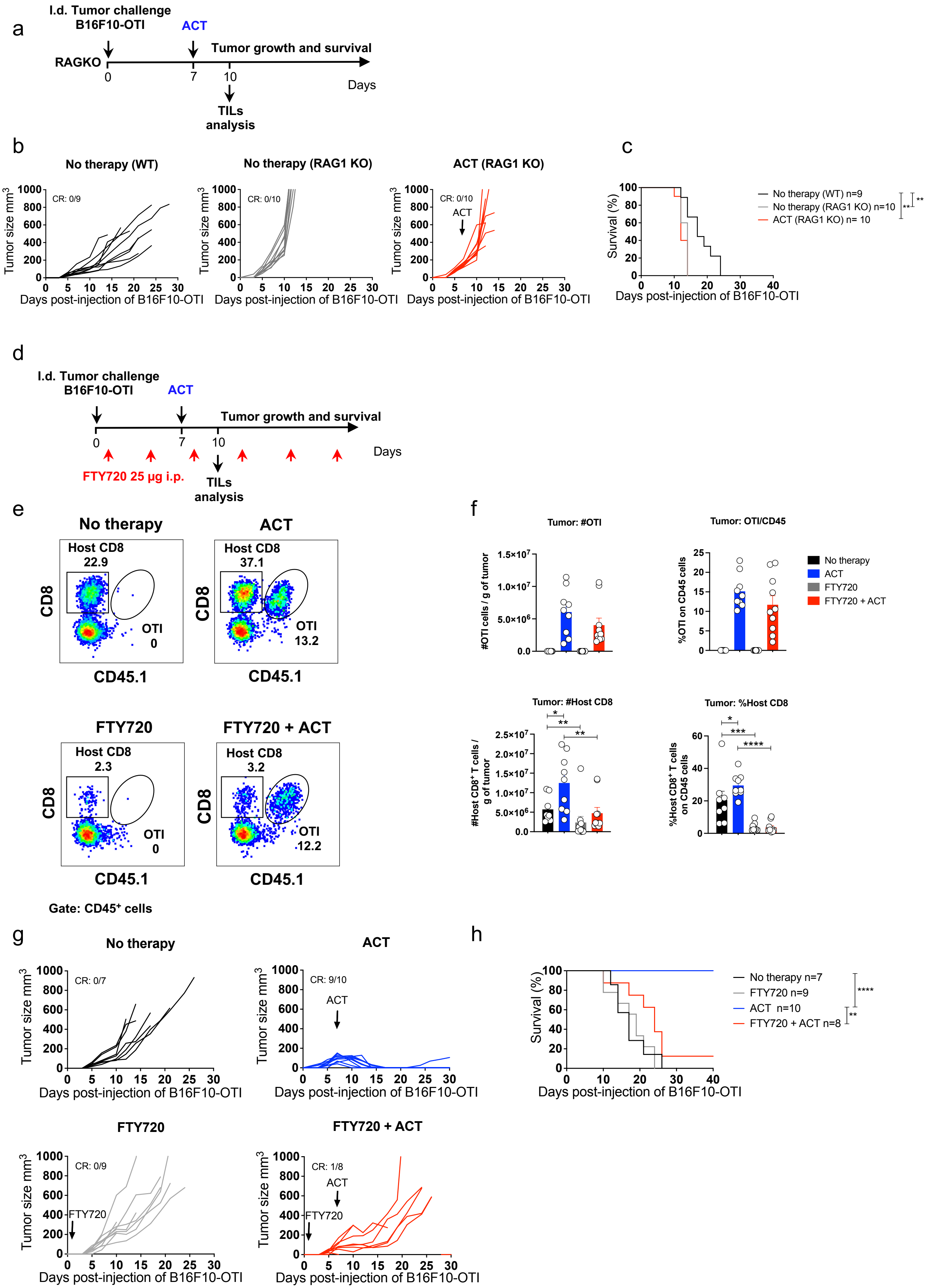

a
I.d. Tumor challenge
B16F10-OTI
ACT
RAGKO
Tumor growth and survival
0
7
10
Days
TILs analysis
b
No therapy (WT)
No therapy (RAG1 KO)
ACT (RAG1 KO)
CR: 0/9
CR: 0/10
CR: 0/10
Tumor size mm3
Days post-injection of B16F10-OTI
c
Survival (%)
No therapy (WT) n=9
No therapy (RAG1 KO) n=10
ACT (RAG1 KO) n= 10
Days post-injection of B16F10-OTI
d
FTY720 25 μg i.p.
e
No therapy
ACT
FTY720
FTY720 + ACT
Host CD8
22.9
37.1
2.3
3.2
OTI
0
13.2
0
12.2
CD8
CD45.1
Gate: CD45+ cells
f
Tumor: #OTI
Tumor: OTI/CD45
Tumor: #Host CD8
Tumor: %Host CD8
#OTI cells / g of tumor
%OTI on CD45 cells
#Host CD8+ T cells / g of tumor
%Host CD8+ T cells on CD45 cells
No therapy
ACT
FTY720
FTY720 + ACT
g
No therapy
ACT
FTY720
FTY720 + ACT
CR: 0/7
CR: 9/10
CR: 0/9
CR: 1/8
h
No therapy n=7
FTY720 n=9
ACT n=10
FTY720 + ACT n=8
Survival (%)
Days post-injection of B16F10-OTI

Supplementary Figure 2. Host T cells are required for ACT-induced tumor eradication. a-c RAG1 KO mice bearing B16F10-OTI tumors received i.v. transfer of $1x10^6$ in vitro activated OTI $CD8^+$ T cells (ACT). WT C57BL/6 untreated mice (No therapy) were used as controls. **a** Experimental timeline. **b-c** Individual tumor growth (**b**) and Kaplan-Meier (**c**) curves for each group: No therapy WT mice (black curves), No therapy RAG1 KO mice (grey curves), and ACT RAG1 KO mice (red curves). **d-h** C57BL/6 mice bearing B16F10-OTI tumors received i.p. injections of FTY720 every three days starting one day after the tumor challenge. Mice were i.v. transferred with $1x10^6$ in vitro activated OTI $CD8^+$ T cells (ACT). **d** Experimental scheme. **e-f** Tumor infiltrating lymphocytes were analyzed by flow cytometry three days after ACT. **e** Representative dot plots displaying the frequencies of host (square, $CD45.1^-$) and transferred (ellipse, OTI $CD45.1^+$) $CD8^+$ T cells within live $CD45^+$ cells for each group: No therapy, ACT, No therapy + FTY720, and ACT + FTY720. **f** Absolute quantifications (cells/g tumor) and relative (on $CD45^+$ cells) of transferred OTI (upper panel) and host (lower panel) $CD8^+$ T cells for each group: No therapy (black bars), ACT (blue bars), No therapy + FTY720 (grey bars), ACT + FTY720 (red bars). **g-h** Individual tumor growth (**g**) and Kaplan-Meier (**h**) curves for each group: No therapy (black curves), ACT (blue curves), No therapy + FTY720 (gray curves), ACT + FTY720 (red curves). (**c**) Results from two independent experiments. Statistical significance was assessed using two-sided log-rank Mantel-Cox test $^{**}P<0.01$, $^{***}P< 0.001$. (**f**) Pooled data from two independent experiments, No therapy n=8, ACT n=9, No therapy + FTY720 n=10, and ACT + FTY720 n=10. Bars are the mean ± SEM. Statistical significance was assessed using two-sided unpaired Mann-Whitney test $^{*}P<0.05$, $^{**}P<0.01$, $^{***}P<0.001$, $^{****}P<0.0001$. (**h**) Results from two independent experiments. Statistical significance was assessed using two-sided log-rank Mantel-Cox test. $^{*}P<0.05$, $^{****}P<0.0001$**.** CR = Complete Response; TP = Total Protection.

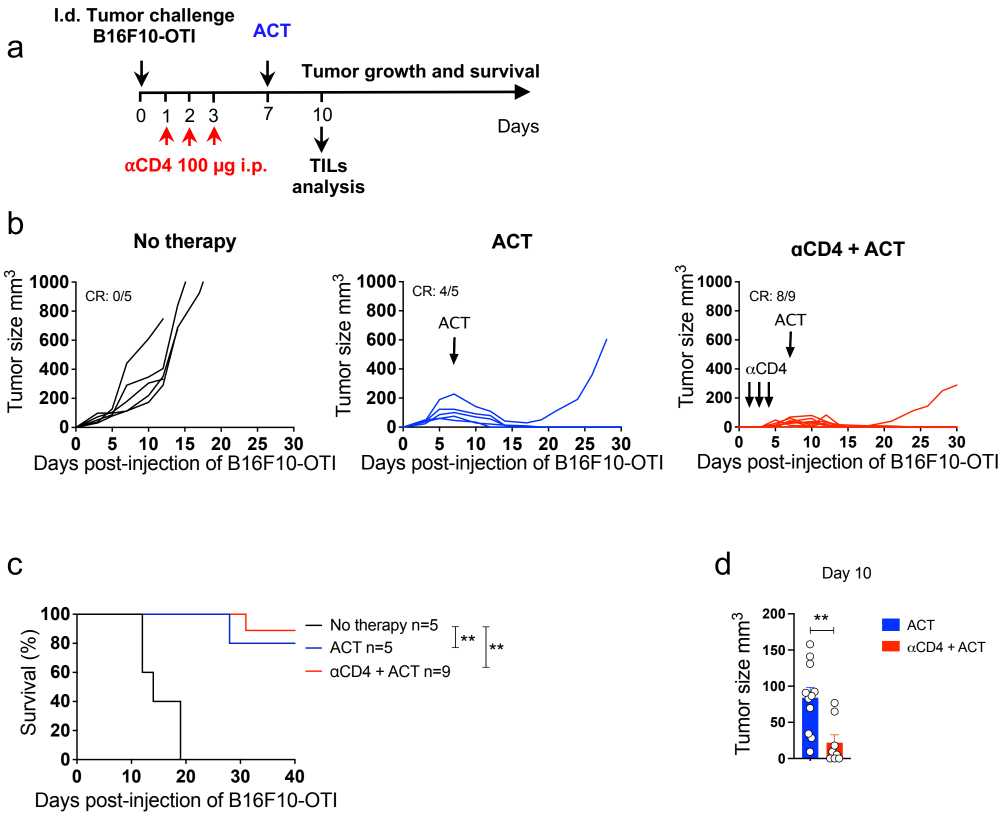

Supplementary Figure 3. CD4⁺ T cell depletion does not affect ACT-mediated tumor eradication. C57BL/6 mice bearing B16F10-OTI tumors received i.v. transfer of $1x10^6$ in vitro activated OTI $CD8^+$ T cells (ACT). A group of mice received three i.p. daily doses of αCD4 antibody starting one day after tumor challenge. Untreated mice (No therapy) were used as controls. **a** Experimental timeline. **b-c** Individual tumor growth (**b**) and Kaplan-Meier (**c**) curves for each group: No therapy (black curves), ACT (blue curves), αCD4 + ACT (red curves). **d** day 10 tumor size quantification (**c**) Results from two independent experiments. Statistical significance was assessed using two-sided log-rank Mantel-Cox test **P<0.01. (**d**) Pooled data from two independent experiments, ACT n=11; αCD4 + ACT n=8. Bars are the mean ± SEM. Statistical significance was assessed using two-sided unpaired Mann-Whitney test **P<0.01. CR = Complete Response.

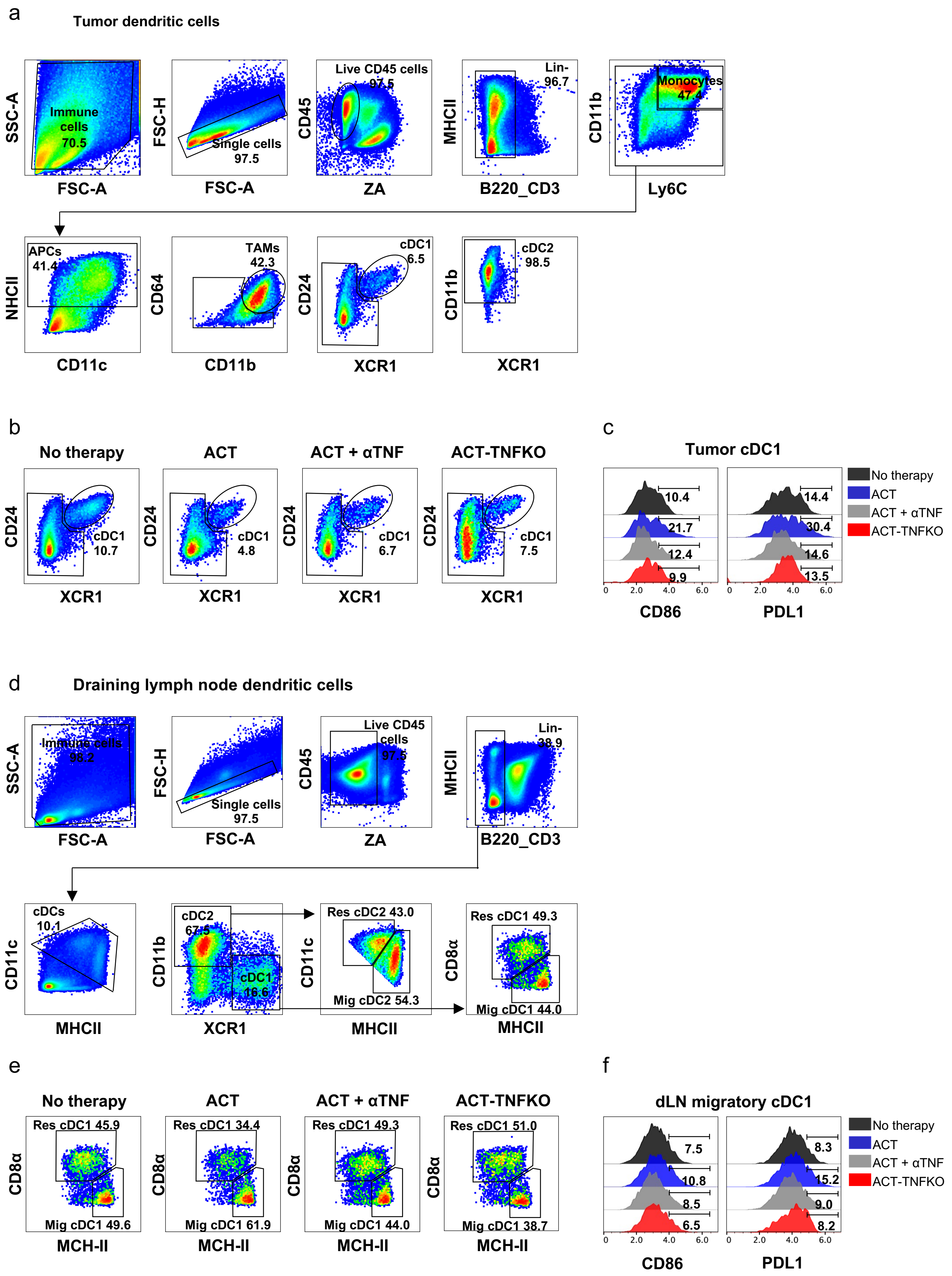
a
Tumor dendritic cells
Immune cells 70.5
SSC-A
FSC-A
Single cells 97.5
FSC-H
FSC-A
Live CD45 cells 97.5
CD45
ZA
Lin- 96.7
MHCII
B220_CD3
Monocytes 47.4
CD11b
Ly6C
APCs 41.4
NHCII
CD11c
TAMs 42.3
CD64
CD11b
cDC1 6.5
CD24
XCR1
cDC2 98.5
CD11b
XCR1
b
No therapy
ACT
ACT + αTNF
ACT-TNFKO
cDC1 10.7
cDC1 4.8
cDC1 6.7
cDC1 7.5
CD24
XCR1
c
Tumor cDC1
10.4
21.7
12.4
9.9
14.4
30.4
14.6
13.5
0
2.0
4.0
6.0
CD86
PDL1
No therapy
ACT
ACT + αTNF
ACT-TNFKO
d
Draining lymph node dendritic cells
Immune cells 98.2
SSC-A
FSC-A
Single cells 97.5
FSC-H
FSC-A
Live CD45 cells 97.5
CD45
ZA
Lin- 38.9
MHCII
B220_CD3
cDCs 10.1
CD11c
MHCII
cDC2 67.5
cDC1 16.6
CD11b
XCR1
Res cDC2 43.0
Mig cDC2 54.3
CD11c
MHCII
Res cDC1 49.3
Mig cDC1 44.0
CD8α
MHCII
e
No therapy
ACT
ACT + αTNF
ACT-TNFKO
Res cDC1 45.9
Mig cDC1 49.6
Res cDC1 34.4
Mig cDC1 61.9
Res cDC1 49.3
Mig cDC1 44.0
Res cDC1 51.0
Mig cDC1 38.7
CD8α
MCH-II
f
dLN migratory cDC1
7.5
10.8
8.5
6.5
8.3
15.2
9.0
8.2
0
2.0
4.0
6.0
CD86
PDL1
No therapy
ACT
ACT + αTNF
ACT-TNFKO

Supplementary Figure 4. Dendritic cell analysis by flow cytometry in tumors and draining lymph nodes. CD45^{+} cells from tumor and draining lymph node of B16F10-OTI melanoma tumors bearing mice were isolated and stained for flow cytometry analysis. **a-b** Analysis of tumor-infiltrating DCs. Gating strategy to define cDC1 and cDC2. **b** Representative dot-plots showing the expression of CD24 and XCR1 within DC population (Lin^{-}CD11c^{+}MHCII^{+}) of each group: No therapy, ACT, ACT + TNF and ACT-TNFKO **c** Representative histograms showing normalized expression of CD86 and PDL1 in cDC1. **d-e** Analysis of DCs in draining lymph nodes. **d** Gating strategy to define lymph node-resident and migratory cDC1 and cDC2. **e** Representative dot-plots of each group: No therapy, ACT, ACT + TNF and ACT-TNFKO. **f** Representative histograms showing the normalized expression of CD86 and PDL1 in migratory cDC1.

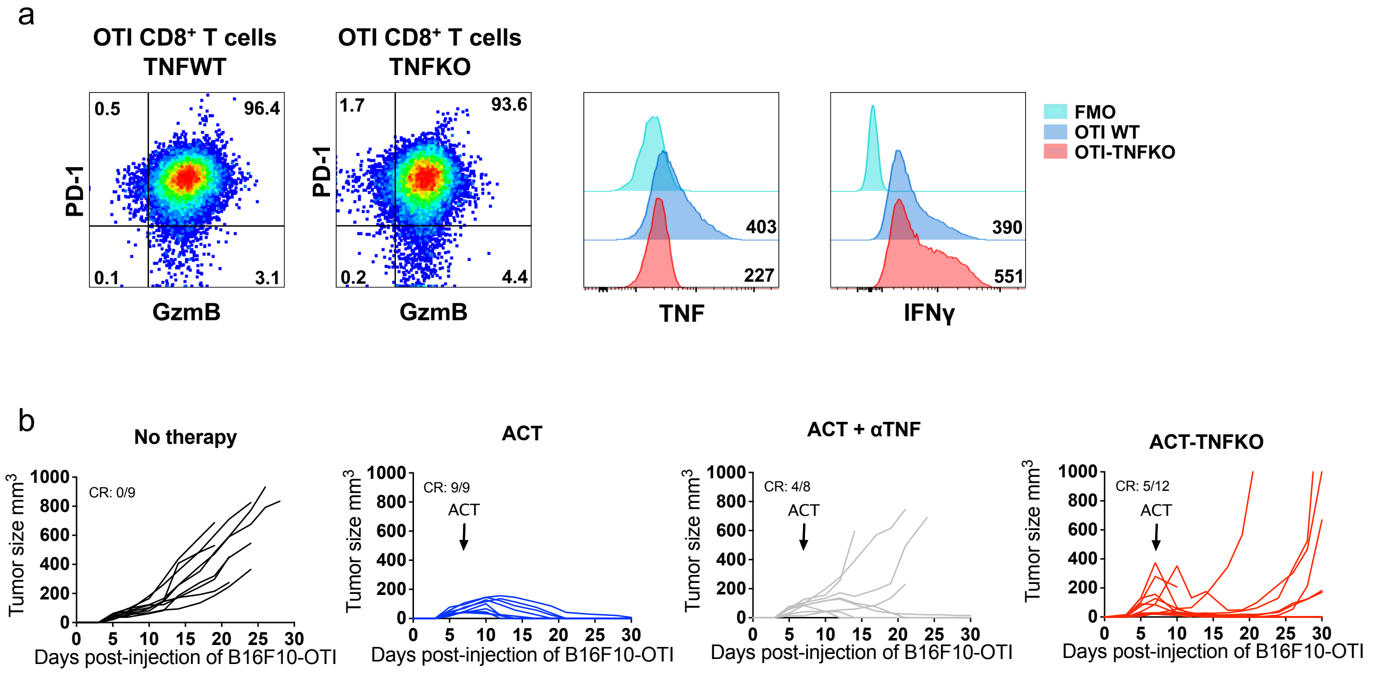

Supplementary Figure 5. Effector phenotype and *in vivo* antitumor activity of WT and TNF-KO OTI CD8$^+$ T cells. a In vitro activated WT and TNFKO OTI CD8$^+$ T cells were reactivated with OTI peptide in the presence of brefeldin A to analyze intracellular TNF and IFNγ production. Representative dot plots showing the expression of PD-1 and GzmB (Left panels). Representative histograms showing the expression of TNF and IFNγ by flow cytometry of WT and TNFKO CD8$^+$ T cells. b-c C57BL/6 mice bearing B16F10-OTI tumors received i.v. transfer of 1x10^6 in vitro activated WT (ACT) or TNFKO (ACT-TNFKO) OTI CD8$^+$ T cells. **b** Individual tumor growth curves of each group: No therapy (black curves), ACT (blue curves), ACT + αTNF (gray curves) and ACT-TNFKO (red curves). **c** Mice that eliminated primary tumors following ACT or ACT-TNFKO were rechallenged with wild-type B16F10 cells in the opposite flank. Individual tumor growth curves of each group: Control (black curves), ACT (blue curves), and ACT-TNFKO (red curves). CR = Complete Response.

a

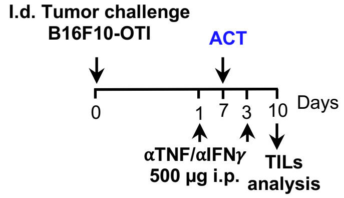

b

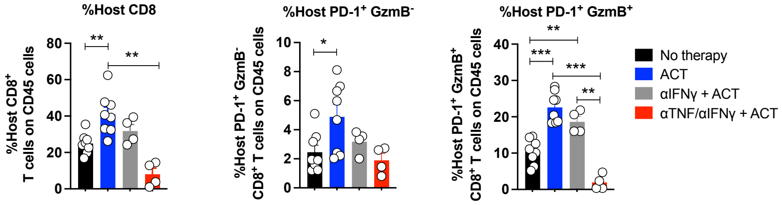

Supplementary Figure 6. IFNγ blockade does not affect ACT-induced tumor accumulation of host CD8⁺ T cells. a-b C57BL/6 mice bearing B16F10-OTI tumors received i.v. transfer of 1x106 in vitro activated OTI $CD8^+$ T cells (ACT). Mice also received α IFNγ antibody i.p. alone or in combination with αTNF every other day starting one day before ACT. Tumor infiltrating lymphocytes were analyzed by flow cytometry three days after ACT. **a** Experimental design. **b** Relative quantification of host $CD8^+$ T cells and their PD-1^+ $GzmB^-$ and PD-1^+ $GzmB^+$ subpopulation on $CD45^+$ cells. (**b**) Data from two independent experiments, No therapy n=8; ACT n=8; αIFNγ + ACT n=4; αTNF/αIFNγ + ACT n=4. Bars are the mean ± SEM. Statistical significance was assessed using two-sided unpaired Mann-Whitney test *P<0.05, **P<0.01, ***P<0.001, ****P<0.0001.

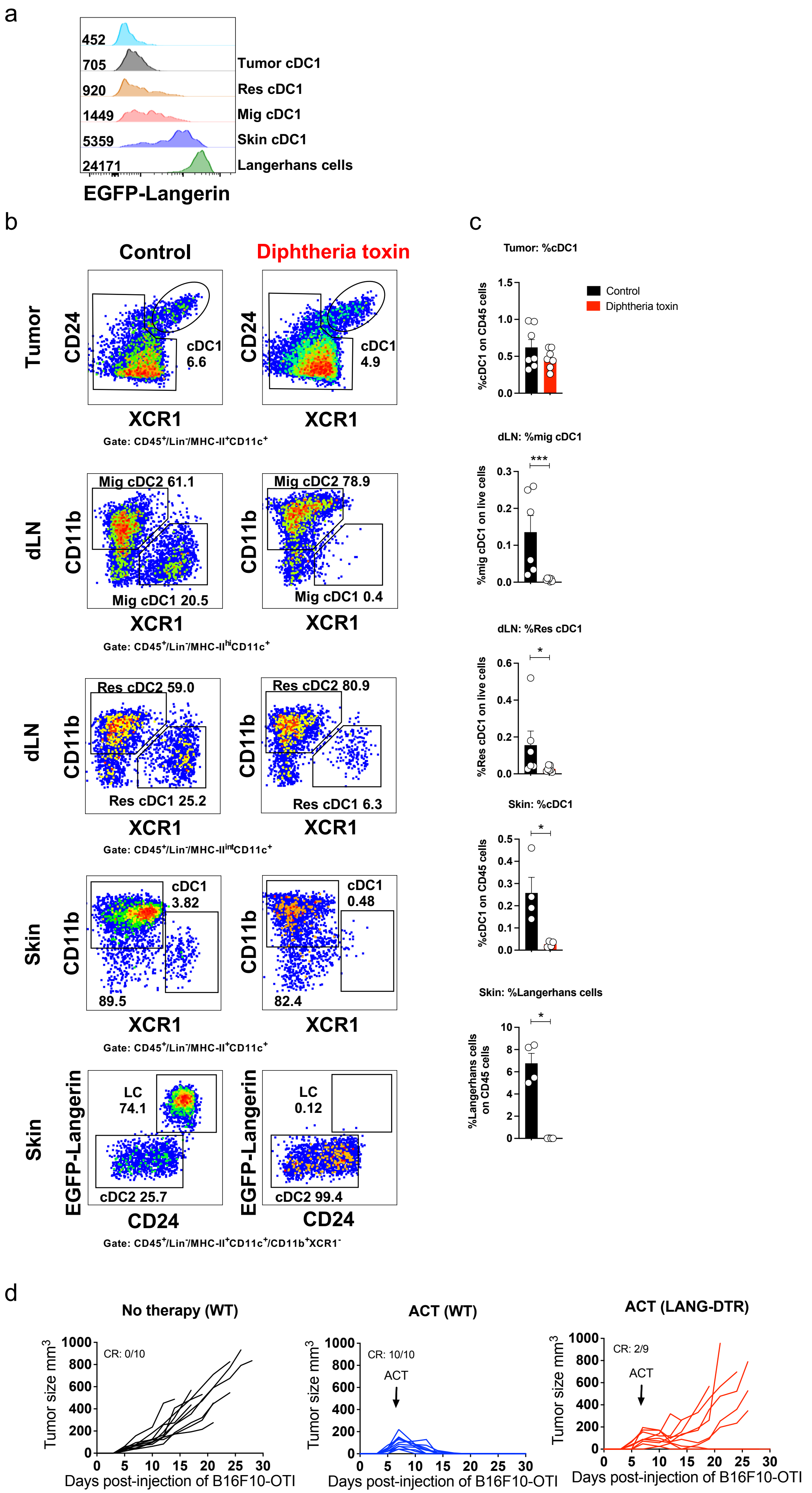
a
452
705
920
1449
5359
24171
Tumor cDC1
Res cDC1
Mig cDC1
Skin cDC1
Langerhans cells
EGFP-Langerin
b
Control
Diphtheria toxin
Tumor
CD24
XCR1
cDC1 6.6
cDC1 4.9
Gate: CD45+/Lin-/MHC-II+CD11c+
dLN
CD11b
Mig cDC2 61.1
Mig cDC1 20.5
Mig cDC2 78.9
Mig cDC1 0.4
Gate: CD45+/Lin-/MHC-IIhiCD11c+
Res cDC2 59.0
Res cDC1 25.2
Res cDC2 80.9
Res cDC1 6.3
Gate: CD45+/Lin-/MHC-IIintCD11c+
Skin
cDC1 3.82
89.5
cDC1 0.48
82.4
Gate: CD45+/Lin-/MHC-II+CD11c+
EGFP-Langerin
LC 74.1
cDC2 25.7
LC 0.12
cDC2 99.4
Gate: CD45+/Lin-/MHC-II+CD11c+/CD11b+XCR1-
c
Tumor: %cDC1
Control
Diphtheria toxin
%cDC1 on CD45 cells
dLN: %mig cDC1
%mig cDC1 on live cells
***
dLN: %Res cDC1
%Res cDC1 on live cells
*
Skin: %cDC1
%cDC1 on CD45 cells
*
Skin: %Langerhans cells
%Langerhans cells on CD45 cells
*
d
No therapy (WT)
CR: 0/10
ACT (WT)
CR: 10/10
ACT
ACT (LANG-DTR)
CR: 2/9
ACT
Tumor size mm3
Days post-injection of B16F10-OTI

Supplementary Figure 7. Dendritic cell analysis and ACT-induced antitumor efficacy in Langerin-DTR mice. Lang-DTR mice bearing B16F10-OTI tumors were processed to analyze EGFP-langerin expressing DCs from tumor, skin and draining lymph node as well as Langerhans cells from skin. **a** Representative histograms showing the expression of EGFP-Langerin in cDC1 across different tissues and Langerhans cells from skin. **b** Mice receiving i.v. 1 µg of diphtheria toxin or control vehicle were analyzed three days later. Representative dot-plots showing frequencies of cDC1 and Langerhans cells in the different tissues. **c** Quantifications of: cDC1 in tumors; migratory and resident cDC1 in draining lymph nodes; cDC1 and Langerhans cells in skin. **d** Individual tumor growth curves of WT and LANG-DTR mice treated with ACT: No therapy in WT (black curves), ACT in WT (blue curves), and ACT in LANG-DTR (red curves). (**c**) Tumor: Pool of two independent experiments, Control n=7; Diphtheria toxin n=7. dLN: Pool of two independent experiments, Control n=6; Diphtheria toxin n=6. Skin: One experiment, Control n=4; Diphtheria toxin n=4. Bars are the mean ± SEM. Statistical significance was assessed using two-sided unpaired Mann–Whitney test $^{*}P<0.05$, $^{***}P<0.001$.

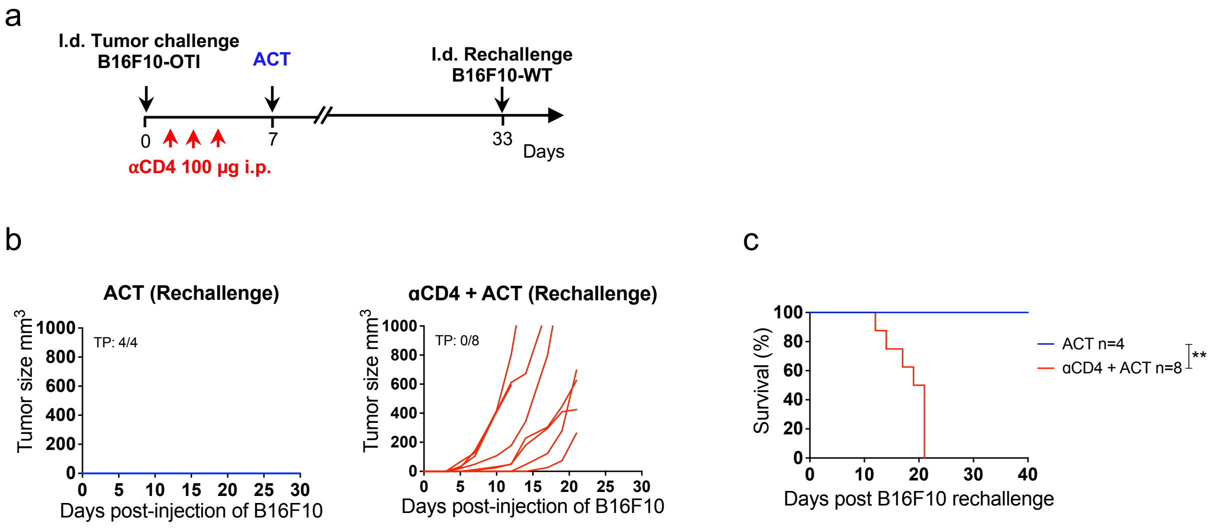

Supplementary Figure 8. CD4$^+$ T cell depletion prior to ACT-mediated primary tumor elimination abolishes protection against rechallenge with ACT-resistant tumor cells. C57BL/6 mice bearing B16F10-OTI tumors received three i.p. daily doses of αCD4 antibody starting one day after tumor challenge, then 1x10^6 in vitro activated OTI CD8$^+$ T cells (ACT) were administrated by i.v. injection. Mice that eliminated primary tumors following ACT were rechallenged with 1x10^6 wild-type B16F10 cells. **a** Experimental timeline. **b,c** Individual tumor growth (**b**) and Kaplan-Meier (**c**) curves of rechallenged groups: ACT (blue curves), αCD4 + ACT. (**c**) Results from two independent experiments. Statistical significance was assessed using two-sided log-rank Mantel-Cox test. **P<0.01. TP = Total Protection.

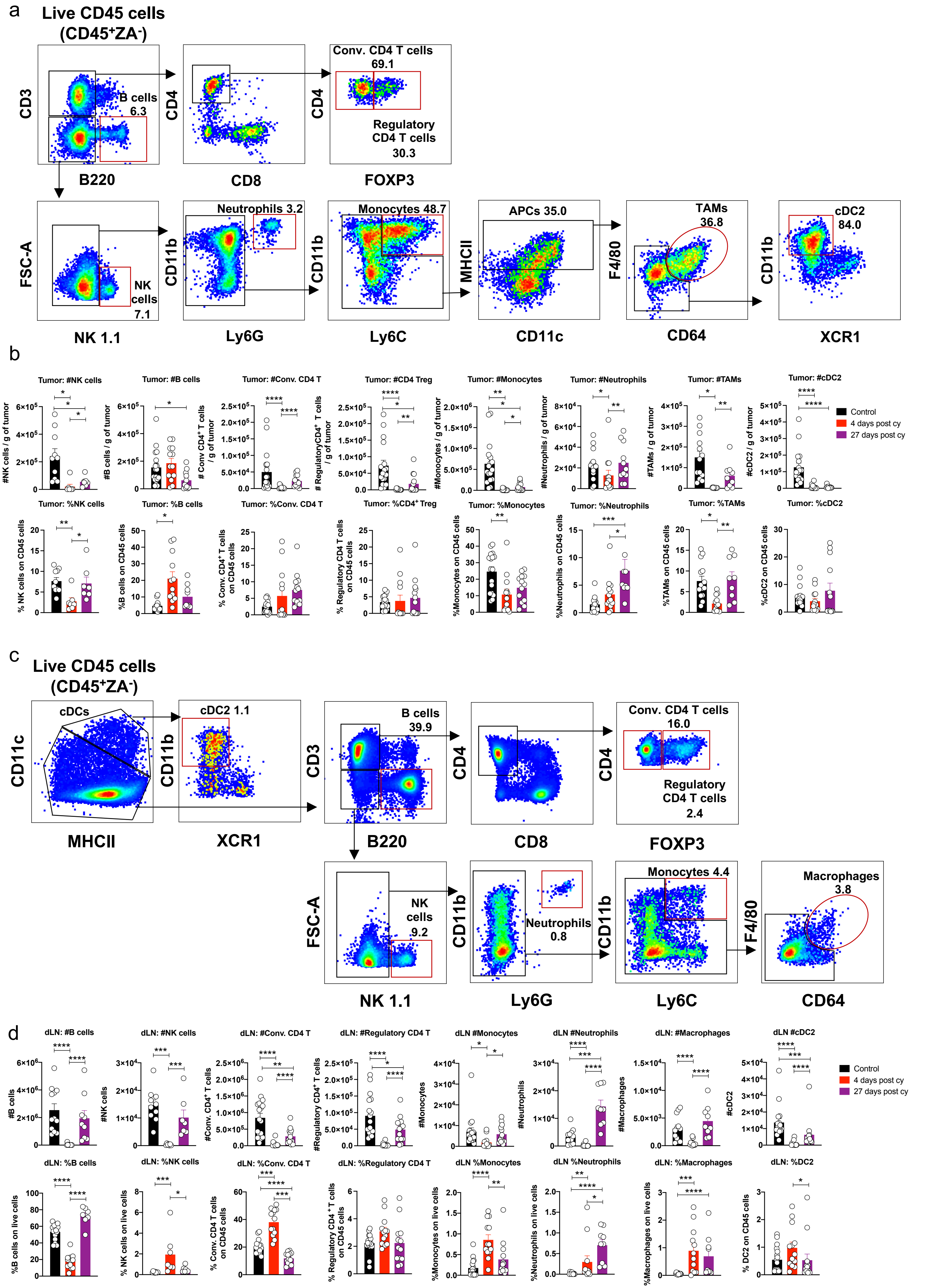
a
Live CD45 cells
(CD45+ZA-)
CD3
B220
B cells 6.3
CD4
CD8
Conv. CD4 T cells 69.1
Regulatory CD4 T cells 30.3
FOXP3
FSC-A
NK 1.1
NK cells 7.1
CD11b
Ly6G
Neutrophils 3.2
Ly6C
Monocytes 48.7
MHCII
CD11c
APCs 35.0
F4/80
CD64
TAMs 36.8
XCR1
cDC2 84.0
b
Tumor: #NK cells
Tumor: #B cells
Tumor: #Conv. CD4 T
Tumor: #CD4 Treg
Tumor: #Monocytes
Tumor: #Neutrophils
Tumor: #TAMs
Tumor: #cDC2
Control
4 days post cy
27 days post cy
Tumor: %NK cells
Tumor: %B cells
Tumor: %Conv. CD4 T
Tumor: %CD4+ Treg
Tumor: %Monocytes
Tumor: %Neutrophils
Tumor: %TAMs
Tumor: %cDC2
c
Live CD45 cells
(CD45+ZA-)
CD11c
MHCII
cDCs
CD11b
XCR1
cDC2 1.1
CD3
B220
B cells 39.9
CD4
CD8
Conv. CD4 T cells 16.0
Regulatory CD4 T cells 2.4
FOXP3
FSC-A
NK 1.1
NK cells 9.2
CD11b
Ly6G
Neutrophils 0.8
Ly6C
Monocytes 4.4
F4/80
CD64
Macrophages 3.8
d
dLN: #B cells
dLN: #NK cells
dLN: #Conv. CD4 T
dLN: #Regulatory CD4 T
dLN #Monocytes
dLN #Neutrophils
dLN: #Macrophages
dLN #cDC2
Control
4 days post cy
27 days post cy
dLN: %B cells
dLN: %NK cells
dLN: %Conv. CD4 T
dLN: %Regulatory CD4 T
dLN %Monocytes
dLN %Neutrophils
dLN: %Macrophages
dLN: %DC2

Supplementary Figure 9. Cyclophosphamide preconditioning effects on NK cells, B cells, myeloid cells and CD4$^+$ T cell subsets in tumors and draining lymph nodes. C57BL/6 mice bearing B16F10-OTI tumors received i.p. administration of cyclophosphamide (Cy, 300 mg/kg) on days 4 and 6 post-tumor challenge. Tumors and draining lymph nodes were analyzed at 4 and 27 days post-treatment. **a-b** Flow cytometry analysis of tumors infiltrating immune cells a Representative dot plots showing gating strategies on live CD45$^+$ cells for NK cells, B cells, neutrophils, monocytes, tumor-associated macrophages (TAMs), cDC2 and conventional CD4$^+$ and regulatory CD4$^+$ T cell subsets highlighted in red squares. **b** Absolute (cells/g) and relative (frequency among CD45$^+$ cells) quantifications of NK cells, B cells, neutrophils, monocytes, TAMs, cDC2 and conventional CD4$^+$ and regulatory CD4$^+$ T cell subsets. **c-d** Flow cytometry analysis of draining lymph nodes. **c** Representative dot plots showing gating strategies for NK cells, B cells, neutrophils, monocytes, macrophages, cDC2 and conventional CD4$^+$ and regulatory CD4$^+$ T cell subsets. **d** Absolute (cells/g) and relative (frequency among CD45$^+$ cells) quantifications of NK cells, B cells, neutrophils, monocytes, macrophages, cDC2 and conventional CD4$^+$ and regulatory CD4$^+$ T cell subsets. Data in (b, d) are pooled from five independent experiments, Control n =17; 4 days post cy n=13; 27 days post cy n=13. Bars represent mean ± SEM. Statistical significance was assessed using two-sided unpaired Mann-Whitney test *P<0.05, **P<0.01, ***P<0.001, ****P<0.0001.

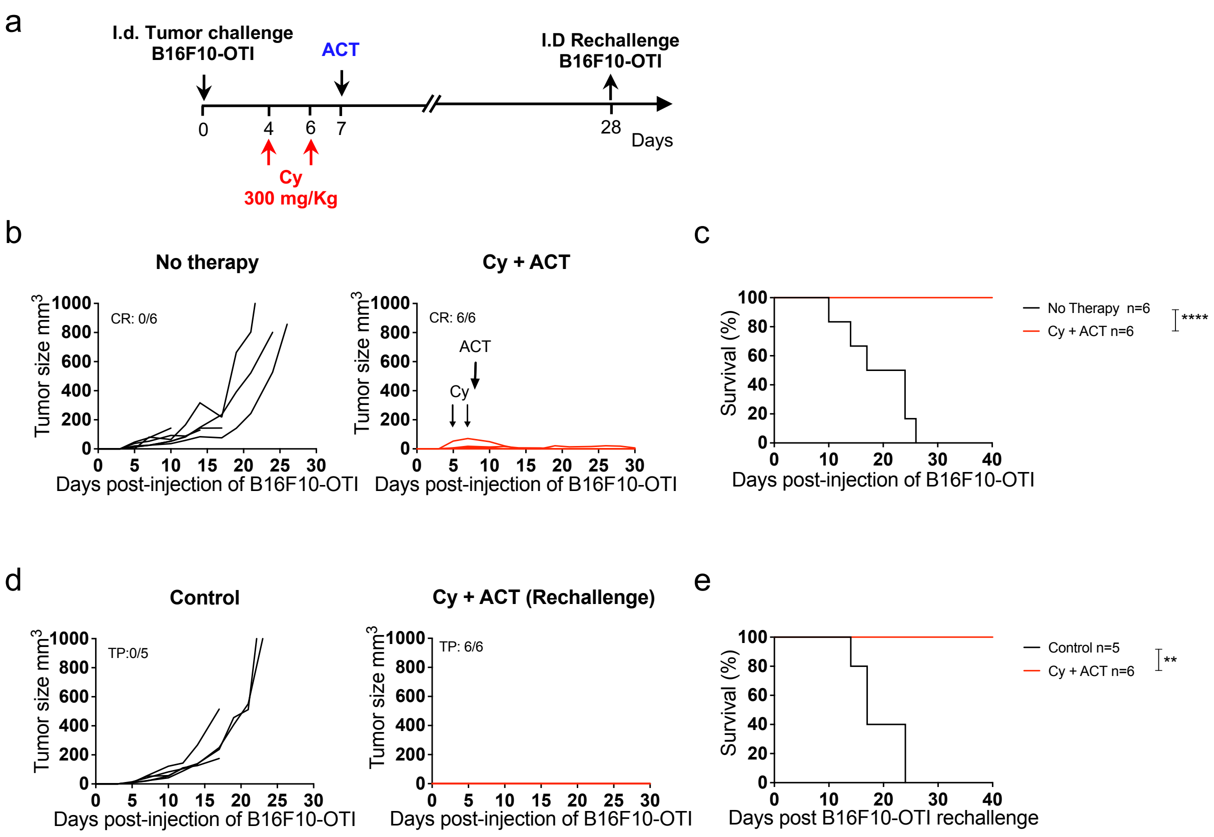

Supplementary Figure 10. Long-term protection against ACT-targeted antigen is maintained in lymphodepleted mice. a-e C57BL/6 mice bearing B16F10-OTI tumors received 300 mg/kg cyclophosphamide (Cy) i.p. on days 4 and 6 after tumor challenge as a preconditioning lymphodepleting regimen. One day later, mice received i.v. transfer of $1x10^6$ *in vitro* activated OTI $CD8^+$ T cells. Mice that rejected primary tumors following Cy + ACT were rechallenged with B16F10-OTI cells in the opposite flank. **a** Experimental scheme **b-c** Individual tumor growth (**b**) and Kaplan-Meier (**c**) curves for each condition: No therapy (black curves) and Cy + ACT (red curves). **d-e** Individual tumor growth (**d**) and Kaplan-Meier (**e**) curves of rechallenged mice: Control (black curves) and Cy + ACT (red curves) (**c, e**) Results from one experiment. Statistical significance was assessed using two-sided log-rank Mantel-Cox test **P<0.01, ****P< 0.0001. CR = Complete Response; TP = Total Protection.

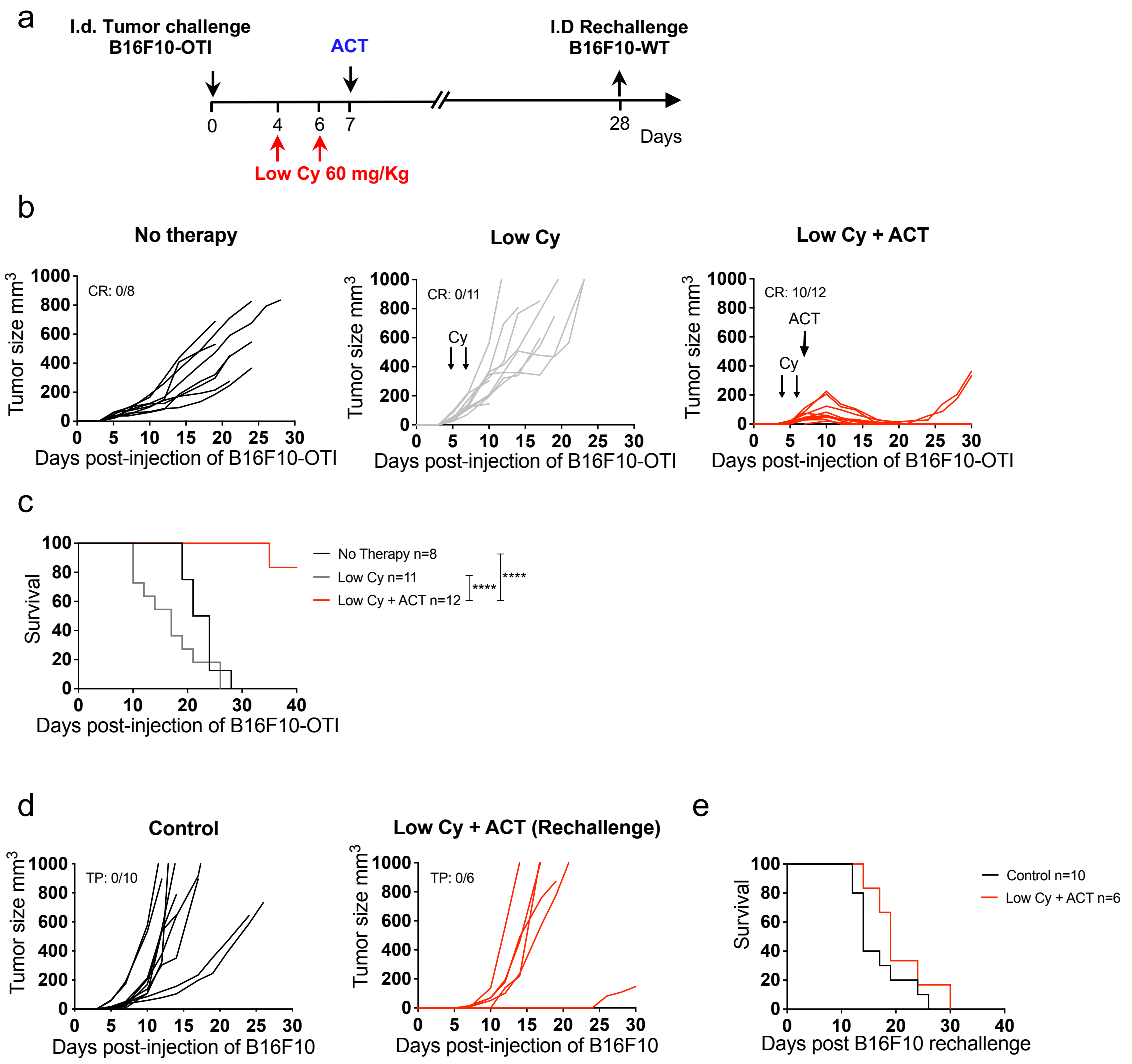

Supplementary Figure 11. Lymphodepleting preconditioning with low dose cyclophosphamide abrogates protection against ACT-resistant tumor cells. C57BL/6 mice bearing B16F10-OTI tumors received 60 mg/kg cyclophosphamide (Cy) i.p. on days 4 and 6 after tumor challenge as a preconditioning lymphodepleting regimen. One day later mice received i.v. transfer of 1×10^6 *in vitro* activated OTI $CD8^+$ T cells. **a** Experimental scheme **b-c** Individual tumor growth (**b**) and Kaplan-Meier (**c**) curves for each group: No therapy (black curves), Low Cy (gray curves) and Low Cy + ACT (red curves). **d-e** Mice that eliminated primary tumors following Low Cy ACT were rechallenged with wild-type B16F10 cells in the opposite flank. Individual tumor growth (**d**) and Kaplan-Meier (**e**) curves of Control (black curves) and Low Cy + ACT mice (red curves). (**c, e**) Results from two independent experiments. Statistical significance was assessed using two-sided log-rank Mantel-Cox test ****P<0.0001. CR = Complete Response; TP = Total Protection.

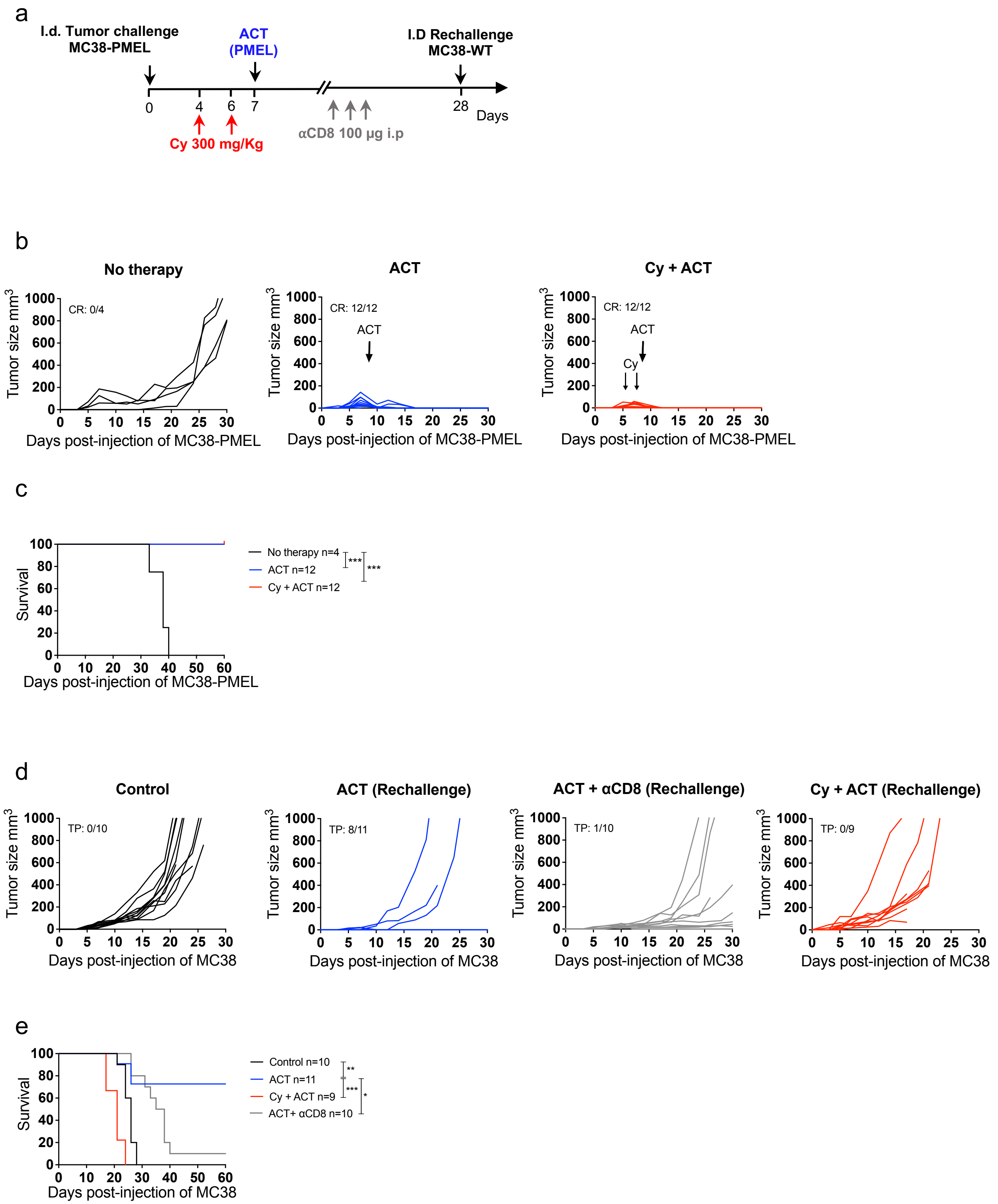

a
I.d. Tumor challenge MC38-PMEL
ACT (PMEL)
I.D Rechallenge MC38-WT
0
4
6
7
28
Days
Cy 300 mg/Kg
αCD8 100 µg i.p
b
No therapy
ACT
Cy + ACT
CR: 0/4
CR: 12/12
CR: 12/12
ACT
Cy
Tumor size mm3
Days post-injection of MC38-PMEL
c
Survival
No therapy n=4
ACT n=12
Cy + ACT n=12
***
***
Days post-injection of MC38-PMEL
d
Control
ACT (Rechallenge)
ACT + αCD8 (Rechallenge)
Cy + ACT (Rechallenge)
TP: 0/10
TP: 8/11
TP: 1/10
TP: 0/9
Tumor size mm3
Days post-injection of MC38
e
Survival
Control n=10
ACT n=11
Cy + ACT n=9
ACT+ αCD8 n=10
**
***
*
Days post-injection of MC38

Supplementary Figure 12. PMEL-1 ACT eliminates MC38-PMEL tumors and confers host $CD8^+$ T cell-mediated protection against ACT-resistant tumor cells that is abrogated by lymphodepleting preconditioning. C57BL/6 mice bearing MC38-PMEL tumors received 300 mg/kg cyclophosphamide (Cy) i.p. on days 4 and 6 after tumor challenge as lymphodepleting preconditioning regimen. One day later, mice received i.v. transfer of $1x10^6$ *in vitro* activated PMEL $CD8^+$ T cells (ACT). Untreated mice (No therapy) were used as controls. Some ACT-treated mice that rejected tumors received three doses of αCD8 before rechallenge. **a** Experimental scheme. **b-c** Individual tumor growth (**b**) and Kaplan-Meier (**c**) curves for each group: No therapy (black curves), and ACT (blue curves) and Cy + ACT (red curves). **d-e** Mice that rejected primary tumors following ACT or Cy + ACT were challenged with MC38 cells in the opposite flank. A group of mice treated with ACT received three i.p. daily doses of αCD8 antibodies prior to rechallenge. Individual tumor growth (**d**) and Kaplan-Meier (**e**) curves for rechallenged group: Control (black curves), ACT (blue curves), ACT + αCD8 (gray curves) and Cy + ACT (red curves). (**c**) Results from two independent experiments. Statistical significance was assessed using two-sided log-rank Mantel-Cox test ***P<0.001. (**e**) Results from two independent experiments. Statistical significance was assessed using two-sided log-rank Mantel-Cox test. *P<0.05, **P<0.01, ***P<0.001. CR = Complete Response; TP = Total Protection.

a

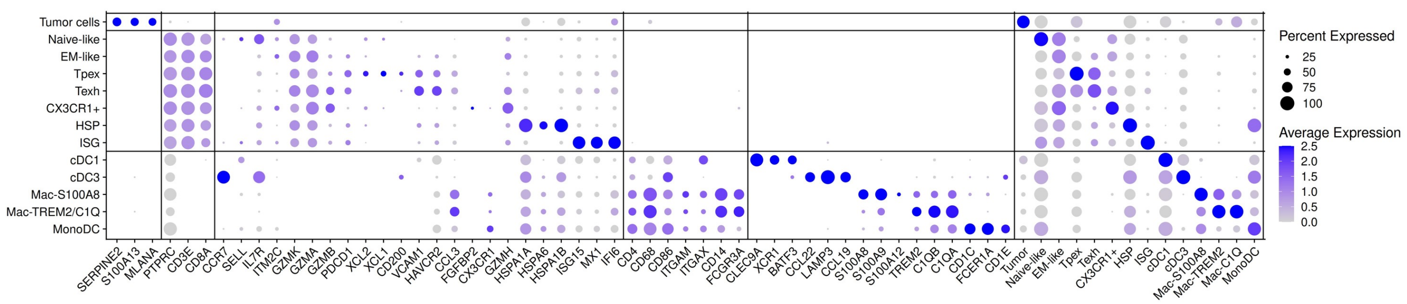

Supplementary Figure 13. Cell type annotation of tumor and immune clusters identified by scRNA-seq in TIL-treated melanoma patients. a Dot plot showing average gene expression (color scale) and percentage of cells expressing each gene (dot size) across the major cell clusters identified in the UMAP analysis (Fig. 7b). Marker genes were selected to annotate Tpex, Tex, cDC1, cDC2, monocyte-derived cells, and tumor cells based on established lineage and functional markers. This analysis was used to annotate clusters in Figure 6b and to define gene signatures for downstream functional and clinical correlation analyses.
