# Supplementary material for: Lymphodepleting preconditioning impairs host antitumor immunity induced by adoptive T cell therapy in mouse models
Source: Nat Commun. 2026 Mar 31;17:4337. doi: 10.1038/s41467-026-71082-y (PMC13172322; doi:10.1038/s41467-026-71082-y)
Supplement: Supplementary file 2 — Description of Additional Supplementary Files [file 41467_2026_71082_MOESM2_ESM.pdf]

Title: Supplementary Data 1

Description: Excel file containing the gene signatures of human immune cell subsets used for the analyses presented in the manuscript
