# Supplementary material for: Lymphodepleting preconditioning impairs host antitumor immunity induced by adoptive T cell therapy in mouse models
Source: Nat Commun. 2026 Mar 31;17:4337. doi: 10.1038/s41467-026-71082-y (PMC13172322; doi:10.1038/s41467-026-71082-y)
Supplement: Supplementary file 4 — Reporting Summary [file 41467_2026_71082_MOESM4_ESM.pdf]

nature portfolio

Corresponding author(s): Alvaro Lladser, Vincenzo Borgna

Last updated by author(s): Jun 27, 2025

Reporting Summary

Nature Portfolio wishes to improve the reproducibility of the work that we publish. This form provides structure for consistency and transparency in reporting. For further information on Nature Portfolio policies, see our Editorial Policies and the Editorial Policy Checklist.

Statistics

For all statistical analyses, confirm that the following items are present in the figure legend, table legend, main text, or Methods section.

n/a	Confirmed	
☐	☒	The exact sample size (*n*) for each experimental group/condition, given as a discrete number and unit of measurement
☐	☒	A statement on whether measurements were taken from distinct samples or whether the same sample was measured repeatedly
☐	☒	The statistical test(s) used AND whether they are one- or two-sided<br>*Only common tests should be described solely by name; describe more complex techniques in the Methods section.*
☒	☐	A description of all covariates tested
☒	☐	A description of any assumptions or corrections, such as tests of normality and adjustment for multiple comparisons
☐	☒	A full description of the statistical parameters including central tendency (e.g. means) or other basic estimates (e.g. regression coefficient) AND variation (e.g. standard deviation) or associated estimates of uncertainty (e.g. confidence intervals)
☐	☒	For null hypothesis testing, the test statistic (e.g. *F*, *t*, *r*) with confidence intervals, effect sizes, degrees of freedom and *P* value noted<br>*Give P values as exact values whenever suitable.*
☒	☐	For Bayesian analysis, information on the choice of priors and Markov chain Monte Carlo settings
☒	☐	For hierarchical and complex designs, identification of the appropriate level for tests and full reporting of outcomes
☒	☐	Estimates of effect sizes (e.g. Cohen's *d*, Pearson's *r*), indicating how they were calculated

Our web collection on statistics for biologists contains articles on many of the points above.

Software and code

Policy information about availability of computer code

Data collection	Publicly available datasets used in this study are cited with accession numbers in the manuscript.
Data analysis	Custom R scripts are publicly available in a GitHub repository and referenced in the Code Availability section.

For manuscripts utilizing custom algorithms or software that are central to the research but not yet described in published literature, software must be made available to editors and reviewers. We strongly encourage code deposition in a community repository (e.g. GitHub). See the Nature Portfolio guidelines for submitting code & software for further information.

Data

Policy information about availability of data

All manuscripts must include a data availability statement. This statement should provide the following information, where applicable:

- Accession codes, unique identifiers, or web links for publicly available datasets
- A description of any restrictions on data availability
- For clinical datasets or third party data, please ensure that the statement adheres to our policy

All raw data underlying the figures are provided in a single Excel file labelled "Source Data," containing one sheet per figure (including Supplementary Figures where applicable)

Research involving human participants, their data, or biological material

Policy information about studies with human participants or human data. See also policy information about sex, gender (identity/presentation), and sexual orientation and race, ethnicity and racism.

Reporting on sex and gender	NA
Reporting on race, ethnicity, or other socially relevant groupings	NA
Population characteristics	NA
Recruitment	NA
Ethics oversight	NA

Note that full information on the approval of the study protocol must also be provided in the manuscript.

Field-specific reporting

Please select the one below that is the best fit for your research. If you are not sure, read the appropriate sections before making your selection.

☒ Life sciences ☐ Behavioural & social sciences ☐ Ecological, evolutionary & environmental sciences

For a reference copy of the document with all sections, see nature.com/documents/nr-reporting-summary-flat.pdf

Life sciences study design

All studies must disclose on these points even when the disclosure is negative.

Sample size	Sample size was determined via a priori power analysis based on means and SDs estimated from pilot experiments and previous experience.
Data exclusions	Mice bearing tumors bigger than 200 mm3 were not considered for flow cytometry analyses. Mice that did not eliminate primary tumors were excluded from rechallenge experiments
Replication	Data presented are pooled from two or three independent experiments unless indicated in the figure legends
Randomization	Mice were allocated randomly in different experimental groups, but no specific randomization test was used
Blinding	Investigators were not blinded during allocation or analysis due to the objective nature of flow cytometry-based quantifications

Reporting for specific materials, systems and methods

We require information from authors about some types of materials, experimental systems and methods used in many studies. Here, indicate whether each material, system or method listed is relevant to your study. If you are not sure if a list item applies to your research, read the appropriate section before selecting a response.

Materials & experimental systems

n/a	Involved in the study	
☐	☒	Antibodies
☐	☒	Eukaryotic cell lines
☒	☐	Palaeontology and archaeology
☐	☒	Animals and other organisms
☒	☐	Clinical data
☒	☐	Dual use research of concern
☒	☐	Plants

Methods

n/a	Involved in the study	
☒	☐	ChIP-seq
☐	☒	Flow cytometry
☒	☐	MRI-based neuroimaging

Antibodies

Antibodies used	Antibodies for in vivo administration were obtained from Bio X Cell: αTNF-α (clone XT3.11, Ref BE0058) or αIFN-γ (clone XMG1.2, Ref BE0055). Monoclonal antibodies for flow cytometry specific for mouse molecules were purchased from Biolegend: CD45-FITC (clone 30-F11), TNF-α-FITC (MP6-XT22) CD279/PD1-PE (clone 29f.1a12), IFN-γ-PE (XMG1.2), CD64-PEDazzle (X54-5/7.1) CD8α -PerCP (clone 53-7.6), PerCP-CD3 (17A2), PerCP-B220 (RA3-6B2), CD45.1-PE/Cy7 (clone A20), CD366/TIM3-PE/Cy7 (clone RMT3-23), PDL1-PE/Cy7 (10F.9G2), CD45.2-APC/Cy7 (clone 104), MHCII-APC/Cy7 (m5/114.15.2), CD3-Brillant Violet 421 (clone 17A2), CD45.1-Brillant Violet 421 (clone A20), CD279/PD1-Brillant Violet 421 (clone 29f.1a12), CD24-Brillant Violet 421 (M1/69), CD366/TIM3-Brillant Violet 605

	(RMT3-23), Ly6C-Brillant Violet 605 (HK1.4), CD8-Brillant Violet 650 (53-6.7), XCR1-Brillant Violet 650 (ZET), CD11c-Brillant Violet 711 (N418), CD11b-Brillant Violet 785 (M1/70). BD Biosciences: CD86-PE (cloneGL1). Cell signaling technology: TCF1/TCF7-AF488 (clone C63D9). Miltenyi Biotec: TOX-APC (REA473) and Invitrogen: Granzyme B-APC (clone GB11), Granzyme B-PE-TexasRed (clone GB11). Viability dye was made with ZombieAqua (Biolegend ref 423101). Samples were acquired in a FACSymphony A1 cytometer, FACSAria III (BD Biosciences) or Aurora (Cytek Biosciences), and data were analyzed using FlowJo version 10.8.1 (Tree Star, Inc.).
Validation	All antibodies were previously used and validated by the commercial provider. Mouse Fc-block was used to prevent unspecific binding of antibodies. Stained samples were consistently compared with unstained controls and fluorescent-minus-one (FMOs) to ensure accuracy of the staining

Eukaryotic cell lines

Policy information about cell lines and Sex and Gender in Research

Cell line source(s)	Mouse melanoma cell line B16F10 (ATCC CLR-6475) was obtained from American Type Culture Collection. MC38 tumor cells were kindly provided by Dr. Sergio A. Quezada (University College London Cancer Institute, UK). B16F10-OTIx5-ZsGreen (B16F10-OTI) and MC38-OTI.hPMELx5-ZsGreen (MC38-PMEL) cells were generated by lentiviral transduction with the pLVX-OTIx5-ZsGreen vector encoding the OTI and PMEL epitopes minigene fused to ZsGreen
Authentication	Transgen expression of OTI or PMEL peptides in cell lines was evaluated by ZsGreen fusion protein expression by flow cytometry
Mycoplasma contamination	Cell lines were frequently tested for Mycoplasma contamination by PCR. Cell lines used were negative for Mycoplasma contamination.
Commonly misidentified lines (See ICLAC register)	NA

Animals and other research organisms

Policy information about studies involving animals; ARRIVE guidelines recommended for reporting animal research, and Sex and Gender in Research

Laboratory animals	C57BL/6/J wild-type (CD45.2), B6.129S7-Rag1tm1Mom/J (RAG1KO), C57BL/6-Tg(TcraTcrb)1100Mjb/J (OT-I), CBy.SJL(B6)-Ptprca/J (CD45.1), B6.129S2-Cd207tm3(DTR/GFP)Mal/J (Langerin-DTR), B6.Cg-Thy1a/Cy Tg(TcraTcrb)8Rest/J (PMEL-1), B6.129S-Tnftm1Gkl/J (TNFαKO) mice were purchased from Jackson Laboratories. Mice were kept at the animal facility of Fundación Ciencia & Vida
Wild animals	NA
Reporting on sex	Male and female mice were used indistinctly. Female mice were preferred for long-term survival analysis to limit complications of male territorial behavior and fighting during long-term cancer experiments
Field-collected samples	NA
Ethics oversight	Mice were maintained according to the “Guide to Care and Use of Experimental Animals, Canadian Council on Animal Care”. This study was carried out in accordance with the recommendations of the “Guidelines for the Welfare and use of Animals in cancer research, Committee of the National Cancer Research Institute”. All procedures complied with all relevant ethical regulations for animal research and were approved by the “Comité de Bioética y Bioseguridad” of Fundación Ciencia & Vida.

Note that full information on the approval of the study protocol must also be provided in the manuscript.

Plants

Seed stocks	*Report on the source of all seed stocks or other plant material used. If applicable, state the seed stock centre and catalogue number. If plant specimens were collected from the field, describe the collection location, date and sampling procedures.*
Novel plant genotypes	*Describe the methods by which all novel plant genotypes were produced. This includes those generated by transgenic approaches, gene editing, chemical/radiation-based mutagenesis and hybridization. For transgenic lines, describe the transformation method, the number of independent lines analyzed and the generation upon which experiments were performed. For gene-edited lines, describe the editor used, the endogenous sequence targeted for editing, the targeting guide RNA sequence (if applicable) and how the editor was applied.*
Authentication	*Describe any authentication procedures for each seed stock used or novel genotype generated. Describe any experiments used to assess the effect of a mutation and, where applicable, how potential secondary effects (e.g. second site T-DNA insertions, mosiacism, off-target gene editing) were examined.*

Flow Cytometry

Plots

Confirm that:

☐ The axis labels state the marker and fluorochrome used (e.g. CD4-FITC).

☒ The axis scales are clearly visible. Include numbers along axes only for bottom left plot of group (a 'group' is an analysis of identical markers).

☒ All plots are contour plots with outliers or pseudocolor plots.

☒ A numerical value for number of cells or percentage (with statistics) is provided.

Methodology

Sample preparation	Tumors, inguinal lymph nodes and skin samples were excised, cut in small fragments and mechanically disaggregated. Samples were resuspended in 1 mL RPMI 1640 medium (ThermoFisher Scientific, ref 61870-036) containing 5 mg/mL of collagenase type IV (Gibco, ref 17104019) and 5 µg/mL of DNAse I (AppliChem, ref A3778,0010) and incubated for 60 (tumors) or 30 (lymph nodes and skin) min at 37 °C with shaking. Samples were then resuspended in 1 mL of supplemented of RPMI 1640 medium (ThermoFisher Scientific, ref 61870-036) containing 5 µg/mL of DNAse I (AppliChem, ref A3778,0010) and incubated for 5 min at 4°C. Skin pieces were mechanically disaggregated using microscope slides with ground edges (Sail Brand, ref 7105). Single cell suspensions were obtained using a 70µm cell strainer (BD Falcon, ref 352350). For the analysis of tumor DCs, CD45 magnetic positive selection (MACS, Miltenyi ref 130-052-301) was used to enrich hematopoietic cells. Single cell suspensions were incubated for 10 min with the TruStain fcX (Biolegend, clone 93, ref 101320). For surface staining, cell suspensions were incubated with the antibodies for 20 minutes at 4ºC followed by two washes with PBS 1x. Cells were then fixed and permeabilized for intracellular and intranuclear staining using the eBioscience FOXP3/transcription factor staining kit (Invitrogen, ref 00-5523-00), followed by intranuclear staining.
Instrument	Samples were acquired in a FACSymphony A1 cytometer, FACSAria III (BD Biosciences) or Aurora (Cytek Biosciences)
Software	Data were analyzed using FlowJo version 10.8.1 (Tree Star, Inc.) GraphPad Prism (version 10.1.9).
Cell population abundance	The abundance of the populations within the tissue or gate of interest is indicated throughout the manuscript
Gating strategy	Gating strategies are indicated in Supplementary figures

☒ Tick this box to confirm that a figure exemplifying the gating strategy is provided in the Supplementary Information.
