# Supplementary material for: Lymphodepleting preconditioning impairs host antitumor immunity induced by adoptive T cell therapy in mouse models
Source: Nat Commun. 2026 Mar 31;17:4337. doi: 10.1038/s41467-026-71082-y (PMC13172322; doi:10.1038/s41467-026-71082-y)
Supplement: Supplementary file 5 — Transparent Peer Review file [file 41467_2026_71082_MOESM5_ESM.pdf]

nature portfolio

Peer Review File

Lymphodepleting preconditioning impairs host antitumor immunity induced by adoptive cell therapy in mouse models

Corresponding Author: Dr Alvaro Lladser

This file contains all reviewer reports in order by version, followed by all author rebuttals in order by version.

Version 1:

Reviewer comments:

Reviewer #1

(Remarks to the Author)
This manuscript addresses a significant topic concerning the interplay between the long-term effectiveness of adoptive cell therapy (ACT) and lymphodepleting preconditioning. The authors illustrate that ACT-induced host CD8+ T cell immunity offers protection against rechallenge with ACT-resistant melanoma cells that lack the targeted antigen, a protection which is compromised by lymphodepleting preconditioning using cyclophosphamide (CTX). Despite the observation of a TNF-α- and cDC1-dependent accumulation of intratumoral endogenous CD8+ T cells and subsequent tumor elimination following ACT, there are notable concerns regarding the overall novelty and impact of the manuscript.
Specific comments:
1. The manuscript primarily provides descriptive accounts without offering novel mechanistic insights into the significance of cDC1 recruitment, TNF-α signaling involvement, and ACT resistance in melanoma. Much of the experimental data appears predictable and aligns with existing studies in the field. A crucial aspect left unclear is the mechanism by which ACT facilitates cDC1 recruitment and activates TNF-α signaling to engage with endogenous CD8+ T cells. While the authors propose a role for TNF-α produced by transferred CD8+ T cells in stimulating cDC1 to enhance endogenous CD8+ T cell immunity, this assertion lacks substantiating evidence. Moreover, the manuscript fails to present data demonstrating whether deletion of cDC1 or blockade of TNF-α would impact host protection against rechallenge with ACT-resistant melanoma cells. As such, the results presented in the manuscript only marginally advance our understanding of cDC1-mediated endogenous CD8+ T cell immunity in the context of ACT.

2. While it's evident from Figure 6 that lymphodepleting preconditioning abolishes long-term host antitumor immunity, correlating with the depletion of endogenous CD8+ T cells, the study falls short in providing crucial evidence to underscore the essential role of endogenous CD8+ T cells and other immune populations in the context of ACT. It's acknowledged that lymphodepletion transiently reduces endogenous CD8+ T cells and alters other immune subsets. Growing evidence indicates dynamic changes in dendritic cells (DCs) and myeloid-derived suppressor cells (MDSCs) following lymphodepletion, leading to a biphasic effect on both endogenous and transferred CD8+ T cells, thereby influencing ACT efficacy and resistance. Furthermore, the validation of lymphodepleting preconditioning's effect could be strengthened by employing sublethal total body irradiation (TBI). Such an approach would provide additional insight into the mechanisms underlying the observed alterations in immune cell populations and their impact on ACT outcomes.
3. The animal studies predominantly centered on a single tumor model, B16-OVA, coupled with OT1 ACT. This narrow focus may introduce ambiguity in data interpretation and limit the generalizability of findings due to the artificial nature of the model antigen OVA. Incorporating additional tumor models that are more clinically relevant to ACT would enhance the robustness of the conclusions drawn from the study and bolster its therapeutic implications.
4. The Tdif subsets exhibit characteristics of an exhausted phenotype. It is unexpected to observe that the Tpex gene signature correlates with improved survival in a larger cohort of melanoma patients (SKCM) from The Cancer Genome Atlas (TCGA) database, as depicted in Fig. 7b. Numerous studies have consistently shown that the presence of a T cell exhaustion signature is associated with immunotherapy resistance and unfavorable prognosis across various cancer types, necessitating clarification. Moreover, it would be beneficial to specify the immune resistance signature in order to better understand its implications within this context.

Reviewer #2

(Remarks to the Author)
In this study, Figueroa et al. investigated the impact of Adoptive Cell Therapy (ACT) on endogenous CD8 T cell activation, finding that TNFα induction by ACT promoted CD8 accumulation in tumors and cross-presentation with DCs. The authors also reported that lymphodepletion, while promoting the ACT expansion, dampened the engagement of the host immune cells.
Most of the manuscript focuses on discussing the antitumor function of OT-I CD8 cells (in the context of ACT) against B16 melanoma tumors expressing OT-I and the role of endogenous immune cells. This is not a novel finding as there are already many reports from various groups highlighting the function of adoptively transferred cells (TCR or CAR) and their interplay with endogenous immune cells, which in mouse models has resulted in eradication of antigen negative clones.
Authors further reported the role of TNFα in the induction of endogenous immunity in the context of ACT treatment. Recent reports have highlighted the role of IL12 and IFNγ in the induction endogenous immunity post ACT. In addition, the role of Tpex population and its interaction with DC1 population has been extensively studied and reported in a recent paper in Cell. Have the authors evaluated the role of IL12, IFNγ or IL18 cytokines? And is the impact of TNFα more important than IFN pathway related cytokines?
CD4 population has been shown to play a major role in the context of ACT (TCR or CAR), especially as it interacts with myeloid population specifically DC through MHCII. Authors highlighted the role of endogenous CD8 T cells in eliminating the ACT-resistant tumors. Do the authors have any insights on the role of CD4 T cells in these studies?
The observation regarding lymphodepletion dampening the endogenous immune response is noteworthy as lymphodepletion is a regimen heavily used prior to ACT. This observation may have an important impact clinically. However, authors should consider modifying the timing of ACT relative to lymphodepletion treatment which could circumvent the issue. Meaning if the timing of ACT relative to lymphodepletion treatment is changed, will it allow for the reconstitution of endogenous T cells and potential induction of endogenous immunity while retaining the expansion and persistence of adoptively transferred cells. Perhaps, timepoint analyses to show when the endogenous immune cells (and what immune subsets) re-emerge post lymphodepletion is required to better understand the timing for the ACT. Also, would these findings be relevant in a second tumor model or is it unique to B16? Overall considering the clinical impact, these results warrant more comprehensive evaluation.

Reviewer #3

(Remarks to the Author)
Figueroa and colleagues report on the role of non-transferred CD8 T cells in adoptive T cell therapy. They use a mouse model of ACT with OVA-specific T cells (OT1). The authors support their findings in mice by analysis of previously published datasets from patients. Major issues should be addressed before publication of this manuscript.

1. The authors base all their conclusions on 1 mouse model that is not reflecting clinical treatment with TILs. It is therefore not correct to conclude that lymphodepleting chemotherapy has a negative effect on TIL treatment that has been established in multiple clinical trials. Additional mouse models are needed to support the claim by the authors.

2. The mouse model used by the authors uses a transgenic receptor and is not based on various T cell clones that are usually transferred in a TIL transfer. Additional mouse models are needed to support the claims of the authors.

3. The gating strategy for Tdif and Tpex should be revised as in the Gzm negative T cell population, a fraction expresses TCF1 corresponding to mixed T cell population. All subsequent figures are based on this gating strategy.

Version 2:

Reviewer comments:

Reviewer #1

(Remarks to the Author)
The MS has been improved by adding animal experiments. I have no further comments.

Reviewer #3

(Remarks to the Author)
Although the authors have responded to my questions the animal models are quite artificial and various lympho-depleting chemotherapies have been tested in ACTs in humans. The authors should note that their data is only validated in mouse models (also in the title) and this finding has to be analyzed in patients.

The authors also claim the cross-presentation is involved but only interference with TNFalpha pathway is experimentally shown. I believe that the authors should re-write their conclusions on this set of experiments.

Reviewer #4

(Remarks to the Author)
Editorial Note; this reviewer was recruited and is providing a mediation on behalf of the absent reviewer 2 this round.

After reviewing this, I think the author did address all the previous reviewer's questions.

This study provides compelling evidence that durable antitumor responses to adoptive $CD8^+$ T cell therapy depend on the host immune system and are mediated through a TNF α–driven cDC1–$CD8^+$ T cell axis. It also highlights the unintended consequence of lymphodepleting preconditioning on long term immunity. The work confirmed many previous studies reveal the impact of ACT on endogenous anti-tumor T cell responses and has potential implications for improving clinical ACT protocols.

The authors could broaden the translational impact by exploring following area.

• The phenotypic analysis focused primarily on T cells (transferred vs host $CD8^+$ T cells) and dendritic cells. While this panel is extensive for T-cell exhaustion states, the study gave less attention to other immune subsets. For example, the authors note that lymphodepletion caused a "profound reduction across all $CD45^+$ immune cell populations" in tumors, but the gating strategy for subsets like myeloid cells, NK cells, or B cells is not shown in detail. The characterization of other cells (e.g. MDSCs defined by CD11b+Gr1+, or tumor-associated macrophages) need improvement.

• The study's assessment of long-term immunity was mainly through a single tumor rechallenge after primary tumor clearance. Mice that eradicated B16-OVA tumors were rechallenged with wild-type B16 (lacking OVA) to test memory response to antigen-loss variants. While this demonstrated the protection, it's unclear the impact on memory CD8 T cell differentiation. Can transferred or endogenous CD8 T cells become tissue resident memory cells?

Version 3:

Reviewer comments:

Reviewer #3

(Remarks to the Author)
All my questions have been addressed in the revised manuscript.

Reviewer #4

(Remarks to the Author)
The authors have adequately addressed all of my concerns, and I have no further comments.

Open Access This Peer Review File is licensed under a Creative Commons Attribution 4.0 International License, which permits use, sharing, adaptation, distribution and reproduction in any medium or format, as long as you give appropriate

credit to the original author(s) and the source, provide a link to the Creative Commons license, and indicate if changes were made.
In cases where reviewers are anonymous, credit should be given to 'Anonymous Referee' and the source.
The images or other third party material in this Peer Review File are included in the article's Creative Commons license, unless indicated otherwise in a credit line to the material. If material is not included in the article's Creative Commons license and your intended use is not permitted by statutory regulation or exceeds the permitted use, you will need to obtain permission directly from the copyright holder.
To view a copy of this license, visit https://creativecommons.org/licenses/by/4.0/

REVIEWER COMMENTS

Reviewer #1 (Remarks to the Author):

This manuscript addresses a significant topic concerning the interplay between the long-term effectiveness of adoptive cell therapy (ACT) and lymphodepleting preconditioning. The authors illustrate that ACT-induced host CD8+ T cell immunity offers protection against rechallenge with ACT-resistant melanoma cells that lack the targeted antigen, a protection which is compromised by lymphodepleting preconditioning using cyclophosphamide (CTX). Despite the observation of a TNF-α- and cDC1-dependent accumulation of intratumoral endogenous CD8+ T cells and subsequent tumor elimination following ACT, there are notable concerns regarding the overall novelty and impact of the manuscript.

Specific comments:

1. The manuscript primarily provides descriptive accounts without offering novel mechanistic insights into the significance of cDC1 recruitment, TNF-α signaling involvement, and ACT resistance in melanoma. Much of the experimental data appears predictable and aligns with existing studies in the field. A crucial aspect left unclear is the mechanism by which ACT facilitates cDC1 recruitment and activates TNF-α signaling to engage with endogenous CD8+ T cells. While the authors propose a role for TNF-α produced by transferred CD8+ T cells in stimulating cDC1 to enhance endogenous CD8+ T cell immunity, this assertion lacks substantiating evidence.

Moreover, the manuscript fails to present data demonstrating whether deletion of cDC1 or blockade of TNF-α would impact host protection against rechallenge with ACT-resistant melanoma cells. As such, the results presented in the manuscript only marginally advance our understanding of cDC1-mediated endogenous CD8+ T cell immunity in the context of ACT.

As noted by the reviewer, our results align with emerging studies highlighting a key role of the host immune system in the context of ACT. We acknowledge the reviewer's comment regarding the need for more robust evidence to delineate the TNF-dependent mechanism and demonstrating whether deletion of cDC1 or blockade of TNF-α would impact host protection against rechallenge with ACT-resistant cells. To dissect the contribution of TNF-α produced by adoptively transferred cells, we generated TNF-KO OTI mice by crossing OTI mice with TNF-KO mice obtained from Jackson (www.jax.org). We observed that ACT with TNF-KO OTI $CD8^+$ T cells did not promote accumulation of host $CD8^+$ T cells (Figure 3b) or cDC1 maturation (Figure 3f-i), resulting in reduced primary tumor elimination (Figure 3c; Supplementary figure 4b; survival rate: 42%), as compared to mice treated with WT OTI $CD8^+$ T cells (Figure 3c; Supplementary figure 4b; survival rate: 100%). Similar results were obtained in ACT-treated mice that received the anti TNF-α blocking antibody. These results indicate that TNF-α derived from transferred cells induces host $CD8^+$ T cell expansion and cDC1 maturation, promoting the control of primary tumors.

Also, we evaluated whether $CD8^+$ T cell-derived TNF-α would impact host protection against rechallenge with ACT-resistant melanoma cells. To this end, mice that survived following ACT with TNF-KO OTI $CD8^+$ T cells were rechallenged with B16F10 cells (Figure 3d). As expected, these mice were not protected and all developed tumors (Figure 3e; Supplementary figure 4c), confirming a key role for TNF-α produced by transferred $CD8^+$ T cells in inducing host protection against ACT-resistant tumor cells. We were unable to do rechallenge experiments in the case of cDC1 deletion, since LangDTR mice (+DTx) treated with ACT did not eliminate primary tumors. Collectively, these results indicate that TNF-α produced by transferred $CD8^+$ T cells stimulate cDC1 and enhance endogenous $CD8^+$ T cell immunity to protect

against ACT-resistant tumor cells. These results provide novel mechanistic insights that highlight TNF-α as a key effector cytokine orchestrating the crosstalk between adoptively transferred and host $CD8^+$ T cells leading to the elimination of primary tumors and protection against ACT-resistant tumor cells.

2. While it's evident from Figure 6 that lymphodepleting preconditioning abolishes long-term host antitumor immunity, correlating with the depletion of endogenous CD8+ T cells, the study falls short in providing crucial evidence to underscore the essential role of endogenous CD8+ T cells and other immune populations in the context of ACT. It's acknowledged that lymphodepletion transiently reduces endogenous CD8+ T cells and alters other immune subsets. Growing evidence indicates dynamic changes in dendritic cells (DCs) and myeloid-derived suppressor cells (MDSCs) following lymphodepletion, leading to a biphasic effect on both endogenous and transferred CD8+ T cells, thereby influencing ACT efficacy and resistance. Furthermore, the validation of lymphodepleting preconditioning's effect could be strengthened by employing sublethal total body irradiation (TBI). Such an approach would provide additional insight into the mechanisms underlying the observed alterations in immune cell populations and their impact on ACT outcomes.

We agree with the reviewer on the importance of providing additional mechanistic insights on how lymphodepletion-induced alterations in immune cell populations may impact ACT outcomes. To this end, we analyzed the dynamic changes in different immune cells, including $CD8^+$ T cell and DC populations, in tumors and draining lymph nodes at two timepoints after cyclophosphamide treatment by flow cytometry (Figure 4a). As expected, we observed a marked reduction of all immune populations four days after cyclophosphamide treatment in both tumors (Figure 4b-e; Supplementary figure 8b) and lymph nodes (Figure 4f-i; Supplementary figure 8d). At 27 days post-cyclophosphamide treatment, tumors exhibited poor infiltration by $CD45^+$ cells and all immune cell subsets (Figure 4c; Supplementary figure 8b), reminiscing of a "cold" tumor. In contrast, the numbers of most immune cell populations in the lymph nodes were restored and comparable to baseline levels (Supplementary figure 8d). However, $CD8^+$ T cells and cDC1, although partially restored, remained reduced relative to untreated control (Figure 4f-i). Notably, we observed that the numbers and proportion of PD-1^+ $CD8^+$ T cells were drastically reduced in both tumors (Figure 4b, c) and draining lymph nodes (Figure 4f, g), which is indicative of impaired priming of $CD8^+$ T cells. These results indicate that cyclophosphamide can induce a long-lasting impairment of antitumor immunity. Unfortunately, we were unable to test sublethal total body irradiation as another lymphodepleting preconditioning regimen due to the lack of appropriate facility in our center. The only facility that we have had access to in the past, the Chilean Commission for Nuclear Energy (https://www.cchen.cl/), does not longer provide the service of irradiating experimentation animals. As an alternative to strengthen our conclusions, we tested lymphodepleting preconditioning using low dose of cyclophosphamide aiming to better preserve the immune system (1). We observed that low dose cyclophosphamide also abrogated long-term protection against ACT-resistant tumor cells (Supplementary figure 10 d, e), indicating that even mild lymphodepleting preconditioning impairs long-term antitumor immunity. Collectively, our findings reveal that lymphodepleting preconditioning impairs host antitumor immunity, undermining long-term ACT efficacy, which may ultimately favor resistance to ACT.

3. The animal studies predominantly centered on a single tumor model, B16-OVA, coupled with OT1 ACT. This narrow focus may introduce ambiguity in data interpretation and limit the generalizability of findings due to the artificial nature of the model antigen OVA. Incorporating additional tumor models that are more clinically relevant to ACT would enhance the robustness of the conclusions drawn from the study and bolster its therapeutic implications.

We also agreed with the reviewer on the need to incorporate additional tumor models that are more clinically relevant to ACT, to enhance the robustness of the conclusions and bolster therapeutic implications. To this end, we generated an MC38 cell line expressing the immunodominant epitope of the melanoma-associated antigen GP100 ($GP100_{25-33}$) also known as PMEL, which can be recognized by $CD8^+$ T cells from the TCR-transgenic mouse model PMEL-1(2). This cell line enabled us to simultaneously test a different and widely used tumor model and a clinically relevant antigen/TCR (3,4) within the same model. Similar to the B16F10-OTI model, subcutaneous MC38-PMEL tumors were effectively eliminated by the administration of *in vitro* activated PMEL-1 $CD8^+$ T cells alone or in combination with cyclophosphamide (Supplementary figure 11a-c). Then, mice that eliminated MC38-PMEL primary tumors were rechallenged with ACT-resistant wild-type MC38 tumor cells. Most mice that received ACT alone were able to reject MC38 rechallenge, whereas all those preconditioned with cyclophosphamide failed to sustain long-term protective immunity (Supplementary Fig. 11d, e). Consistently, $CD8^+$ T cell depletion prior to rechallenge also abrogated tumor rejection in ACT responders, confirming the essential role of host $CD8^+$ T cells in mediating long-term tumor control in this model (Supplementary Fig 11d, e). These results consolidate the role of host $CD8^+$ T cell immunity and the detrimental effects of lymphodepleting preconditioning in long-term protection against ACT-resistant tumor cells in a different tumor model, which represent key findings of our study.

4. The Tdif subsets exhibit characteristics of an exhausted phenotype. It is unexpected to observe that the Tpex gene signature correlates with improved survival in a larger cohort of melanoma patients (SKCM) from The Cancer Genome Atlas (TCGA) database, as depicted in Fig. 7b. Numerous studies have consistently shown that the presence of a T cell exhaustion signature is associated with immunotherapy resistance and unfavorable prognosis across various cancer types, necessitating clarification. Moreover, it would be beneficial to specify the immune resistance signature in order to better understand its implications within this context.

We agree with the reviewer that Tdif subset exhibits an exhausted phenotype, which is more evident in our updated Figure 1h. To avoid confusion and align with the nomenclature commonly used in the literature, we have changed the name from terminally differentiated (Tdif) to exhausted (Tex) cells. As stated by the reviewer, there are various studies associating T cell exhaustion with immunotherapy resistance and unfavorable prognosis across different cancers (5–9). This is consistent with the association of T cell exhaustion with poor survival in some cohorts of the TCGA database, for example, renal cell carcinoma, a type of cancer that responds to immunotherapy (Figure for reviewers 1a, see below). However, other cancer types, including melanoma and lung cancer, have shown a positive association between exhausted T cells and response to immunotherapy and favorable clinical outcomes (10–15; Figure for reviewers 1b, see below). This paradox can be explained because tumor-infiltrating $CD8^+$ T cells expressing PD-1, LAG3, CD39 and/or TIM3 are enriched in expanded clonotypes capable of recognizing autologous tumor cells (16–18). In addition, work across multiple models and human cancers has demonstrated that stem-like progenitor-exhausted $CD8^+$ T (Tpex) cells, expressing TCF-1 and PD-1, dictate positive response to immunotherapy and better survival. Importantly, Tpex cells retain polyfunctionality, long-term persistence and proliferative capacity, and upon antigen recognition

differentiate into Tex cells, which provide effector and cytotoxic activity (20–22). Therefore, the presence of a T cell exhaustion gene signature may reflect an active antitumor immune response commanded by Tpex cells. Indeed, we observed a strong correlation between Tpex- and Tex-associated gene signatures in tumors from the TCGA database and TIL-treated patients (Figure for Reviewers 2a, b, see below). This correlation was confirmed at single-cell resolution in a different cohort of TIL-treated patients with available single-cell RNA sequencing data (Figure for Reviewers 2c, see below). Interestingly, this correlation was stronger in patients who exhibited a favorable response to TIL-ACT. Across all analyses, poor survival was associated with the immune resistance signature, which is a transcriptional program expressed by malignant cells that is linked to T cell exclusion and immune evasion (23). We have now included the gene list for this and all signatures used in the manuscript in Supplementary table 1.

Reviewer #2 (Remarks to the Author):

In this study, Figueroa et al. investigated the impact of Adoptive Cell Therapy (ACT) on endogenous CD8 T cell activation, finding that TNFα induction by ACT promoted CD8 accumulation in tumors and cross-presentation with DCs. The authors also reported that lymphodepletion, while promoting the ACT expansion, dampened the engagement of the host immune cells. Most of the manuscript focuses on discussing the antitumor function of OT-I CD8 cells (in the context of ACT) against B16 melanoma tumors expressing OT-I and the role of endogenous immune cells. This is not a novel finding as there are already many reports from various groups highlighting the function of adoptively transferred cells (TCR or CAR) and their interplay with endogenous immune cells, which in mouse models has resulted in eradication of antigen negative clones.

As highlighted by the reviewer, our findings align with emerging studies demonstrating a key role of the host immune system in the success of ACT. These studies have shown that adoptively transferred T cells can stimulate host immune cells for effective tumor control (24–26). Our revised manuscript extends these findings and provides further mechanistic insights into how adoptively transferred T cells crosstalk with the host immune system and how lymphodepleting preconditioning impact the success of ACT. To our knowledge, our study is the first to demonstrate that adoptively transferred T cells induce, via TNF-α, a cDC1-dependent expansion of host $CD8^+$ T cells, which contribute to elimination of primary tumors and mediate long-term protection against ACT-resistant tumor cells. Notably, our work demonstrates that while lymphodepleting preconditioning promotes primary tumor elimination by expanding transferred cells, it also induces a long-lasting impairment of host antitumor immunity, abrogating protection against ACT-resistant tumor cells. These observations suggest that lymphodepleting preconditioning may facilitate the emergence of antigen-loss tumor cell variants and ultimately promote resistance to ACT, which has significant clinical implications and translational relevance.

Authors further reported the role of TNFα in the induction of endogenous immunity in the context of ACT treatment. Recent reports have highlighted the role of IL12 and IFNγ in the induction endogenous immunity post ACT. In addition, the role of Tpex population and its interaction with DC1 population has been extensively studied and reported in a recent paper in Cell. Have the authors evaluated the role of IL12, IFNγ or IL18 cytokines? And is the impact of TNFα more important than IFN pathway related cytokines?

We thank the reviewer for this insightful comment. We are aware of the important role of IFN-γ and related cytokines, such as IL-12 and IL-18, in the interplay between transferred cells and the host immune system. We have included a paragraph in the introduction highlighting the relevance of this axis. In addition, we evaluated the contribution of IFN-γ to the ability of ACT to expand host $CD8^+$ T cells. To this end, mice received anti IFN-γ blocking antibody starting one day before ACT and analyzed tumor infiltration by flow cytometry (Supplementary Figure 5a). We observed that in the presence of anti IFN-γ, ACT did not significantly affect the frequency of endogenous total or $PD\text{-}1^+$ $CD8^+$ T cell populations (Supplementary Figure 5b), indicating that IFN-γ does not have a predominant role at expanding host $CD8^+$ T cell responses, as compared to TNF-α. However, simultaneous blockade of TNFα and IFN-γ resulted in a synergistic reduction of host $PD1^+GzmB^+$ $CD8^+$ T cells infiltrating tumors (Supplementary Fig. 5b), suggesting that these cytokines likely engage complementary mechanisms that support the expansion and cytotoxic differentiation of $CD8^+$ T cells, respectively. To gain insight into the highly novel mechanism, we dissected the contribution of TNF-α produced by adoptively transferred cells. Our results indicate that TNF-α produced by transferred OTI $CD8^+$ T cells is critical for host $CD8^+$ T cell expansion (Figure 3b) and cDC1 maturation (Figure 3g), resulting in effective control of both primary tumors (Figure 3c) and ACT-resistant rechallenge (Figure 3e). Although our results show a predominant role for ACT-derived TNF-α in promoting host antitumor immunity, IFN-γ likely also contributes to tumor control, given its well-established role in promoting tumor cell killing through multiple mechanisms (27–29).

CD4 population has been shown to play a major role in the context of ACT (TCR or CAR), especially as it interacts with myeloid population specifically DC through MHCII. Authors highlighted the role of endogenous CD8 T cells in eliminating the ACT-resistant tumors. Do the authors have any insights on the role of CD4 T cells in these studies?

We fully agree with the reviewer on the crucial role of $CD4^+$ T cells in the context of ACT. Hence, we assessed the contribution of endogenous $CD4^+$ T cells to ACT-mediated primary tumor elimination and their ability to impact long-term rejection of ACT-resistant melanoma cells, administering an anti-CD4 depleting antibody prior to ACT (Supplementary Figure 3a). We observed that in the absence of $CD4^+$ T cells, primary tumor elimination was not affected (Supplementary Figure 3b, c), indicating that their effector activity is not required for tumor control in our model. Indeed, tumors appeared smaller in mice receiving anti-CD4 antibody compared to ACT alone (Supplementary Figure 3d). Interestingly, mice depleted of $CD4^+$ T cells prior to ACT were unable to reject a subsequent rechallenge with ACT-resistant B16F10 melanoma cells (Supplementary Figure 3e). These results suggest that $CD4^+$ T cells are crucial for establishing durable $CD8^+$ T cell-mediated immunity induced by ACT, consistent with their well-established helper function in supporting memory T cell formation (30–32).

The observation regarding lymphodepletion dampening the endogenous immune response is noteworthy as lymphodepletion is a regimen heavily used prior to ACT. This observation may have an important impact clinically. However, authors should consider modifying the timing of ACT relative to lymphodepletion treatment which could circumvent the issue. Meaning if the timing of ACT relative to lymphodepletion treatment is changed, will it allow for the reconstitution of endogenous T cells and potential induction of endogenous immunity while retaining the expansion and persistence of adoptively transferred cells. Perhaps, timepoint analyses to show when the endogenous immune cells (and what immune subsets) re-emerge post lymphodepletion is required to better understand the timing for the ACT.

As highlighted by the reviewer, lymphodepletion-induced impairment of host antitumor immunity may significantly impact clinical ACT outcomes. To gain insight into the effects of lymphodepletion, we performed additional experiments to analyze different immune cell populations, including $CD8^+$ T cells and cDC1, in tumors and lymph nodes at two different timepoints after cyclophosphamide treatment (Figure 4a). As expected, we observed a marked reduction of all immune cell populations four days after cyclophosphamide treatment (Figure 4b-i, Supplementary figure 8). At 27 days post-cyclophosphamide treatment, tumors exhibited poor infiltration across all immune cell subsets (Figure 4b-e, Supplementary figure 8a, b), resembling a "cold" tumor. By contrast, at this timepoint, the numbers of most immune cell populations were comparable to baseline levels in the lymph nodes (Supplementary figure 8c, d), except for $CD8^+$ T cells and cDC1, which, although partially restored, remained reduced relative to the untreated control (Figure 4f-i). Notably, we observed that the numbers and proportion of $PD\text{-}1^+$ $CD8^+$ T cells were drastically reduced in both tumors (Figure 4c) and draining lymph nodes (Figure 4g), which is indicative of impaired $CD8^+$ T cell priming. These results indicate that cyclophosphamide preconditioning can induce a long-lasting impairment of antitumor immunity, thereby favoring resistance to ACT by the emergence of antigen-loss variants. To emphasize the novelty of these findings we have adapted the title accordingly.

Also, would these findings be relevant in a second tumor model or is it unique to B16? Overall considering the clinical impact, these results warrant more comprehensive evaluation.

We agreed with the reviewer on the importance of incorporating a second tumor model to strengthen the robustness of our conclusions and therapeutic implications. To this end, we generated an MC38 cell line expressing the immunodominant epitope of the melanoma-associated antigen GP100 ($GP100_{25\text{-}33}$) also known as PMEL, which can be recognized by $CD8^+$ T cells from the TCR-transgenic mouse model PMEL-1 (2). This cell line enabled us to simultaneously test a different and widely used tumor model and a clinically relevant antigen/TCR (3,4) within the same model. Similar to the B16F10-OTI model, subcutaneous MC38-PMEL tumors were effectively eliminated by the administration of *in vitro* activated PMEL-1 $CD8^+$ T cells alone or in combination with cyclophosphamide (Supplementary figure 11a-c). Then, mice that eliminated MC38-PMEL primary tumors were rechallenged with ACT-resistant wild-type MC38 tumor cells. Most mice that received ACT alone were able to reject tumor rechallenge, whereas all those preconditioned with cyclophosphamide failed to sustain long-term protective immunity (Supplementary Fig. 11d, e). Consistently, $CD8^+$ T cell depletion prior to rechallenge also abrogated tumor rejection in ACT responders, confirming the essential role of host $CD8^+$ T cells in mediating long-term tumor control in this model (Supplementary Fig 11d, e). These results consolidate the role of host $CD8^+$ T cell immunity and the detrimental effects of lymphodepleting preconditioning in long-term protection against ACT-resistant tumor cells in a different tumor model, which represent key findings of our study.

Reviewer #3 (Remarks to the Author):

Figueroa and colleagues report on the role of non-transferred CD8 T cells in adoptive T cell therapy. They use a mouse model of ACT with OVA-specific T cells (OT1). The authors support their findings in mice by analysis of previously published datasets from patients. Major issues should be addressed before publication of this manuscript.

1. The authors base all their conclusions on 1 mouse model that is not reflecting clinical treatment with TILs. It is therefore not correct to conclude that lymphodepleting chemotherapy has a negative effect on TIL treatment that has been established in multiple clinical trials. Additional mouse models are needed to support the claim by the authors.

We thank the reviewer for the valuable comments. We totally agree that our models resemble a TCR-T rather than a TIL type of ACT. Therefore, we have been more careful in our statements and made clear of the inherent differences and limitations. This discrepancy arises because we corroborated the results obtained in mouse models in human datasets from TIL-treated patients. Even if would have been ideal to analyze datasets from patients treated with TCR-based ACT, we only found robust publicly available datasets coming from TIL-treated patients. In addition, we fully acknowledge that lymphodepleting chemotherapy has a positive effect on ACT by expanding transferred cells and enhancing antitumor effects. Indeed, this is illustrated in Figure 5, where we show that cyclophosphamide expands transferred OTI cells (Figure 5b, c) and improves antitumor efficacy of a suboptimal ACT model (Figure 5d, e). These data align with and support the use of lymphodepleting preconditioning in clinical practice to achieve acute clinical responses. However, we provide evidence that cyclophosphamide also induces a long-lasting impairment in host antitumor immunity (Figure 4), resulting in the inability to control the rechallenge with ACT-resistant tumor cells (Figure 5g,h). This previously underappreciated observation may have significant clinical implications, as lymphodepletion is a regimen widely used in ACT strategies (including TCR-T, CAR-T and TIL) and could therefore could facilitate the emergence of antigen-loss variants, a major mechanism driving ACT resistance in patients (33,34).

2. The mouse model used by the authors uses a transgenic receptor and is not based on various T cell clones that are usually transferred in a TIL transfer. Additional mouse models are needed to support the claims of the authors.

We thank the reviewer for this important comment. We fully agree that ACT protocols based on transgenic TCRs, such as OTI, represent a simplified system without clonal diversity when compared to polyclonal TIL products used in the clinic. To address this limitation and increase the translational robustness of our findings, we implemented an independent tumor model that incorporates a different TCR specificity. Specifically, we generated an MC38 cell line expressing the immunodominant epitope of the melanoma-associated antigen GP100 ($GP100_{25-33}$) also known as PMEL, which is recognized by $CD8^+$ T cells from the TCR-transgenic mouse model PMEL-1 (2). This cell line enabled us to simultaneously test a different and widely used tumor model and a clinically relevant antigen/TCR (3,4) within the same model. Similar to the B16F10-OTI model, subcutaneous MC38-PMEL tumors were effectively eliminated by the administration of *in vitro* activated PMEL-1 $CD8^+$ T cells alone or in combination with cyclophosphamide (Supplementary figure 11a-c). Then, mice that eliminated MC38-PMEL primary tumors were rechallenged with ACT-resistant wild-type MC38 tumor cells. Most mice that received ACT alone were able to reject MC38 rechallenge, whereas all those preconditioned with cyclophosphamide failed to sustain long-term protective immunity (Supplementary Fig. 11d, e). Consistently, $CD8^+$ T cell depletion prior to rechallenge also abrogated tumor rejection in ACT responders, confirming the essential role of host $CD8^+$ T cells in mediating long-term tumor control in this model (Supplementary Fig 11d, e). These results consolidate the role of host $CD8^+$ T cell immunity and the detrimental effects of lymphodepleting preconditioning in long-term protection against ACT-resistant tumor cells in a different tumor model, which represent key findings of our study. While we acknowledge that fully polyclonal TIL models may offer further insights, the use of two antigenically distinct models capturing key aspects of ACT provides strong conceptual support for our conclusions.

3. The gating strategy for Tdif and Tpex should be revised as in the Gzm negative T cell population, a fraction expresses TCF1 corresponding to mixed T cell population. All subsequent figures are based on this gating strategy.

We thank the reviewer for this valuable observation. We agree that the PD-1^{+}GzmB^{-} population there is a fraction of cells that does not express TCF-1 and that further discrimination is required to confidently define Tpex cells in accordance with published criteria (20,21,35–40). First, to align with the nomenclature commonly used in the literature, we changed the name of terminally differentiated (Tdif) to exhausted (Tex) cells. To better visualize the issue raised by the reviewer, we included a new plot showing PD-1 vs TCF-1 and labeled the different quadrant with different colors: PD-1^{-}TCF-1^{-} cells in red; PD-1^{+}TCF-1^{-} or Tex cells in green; PD-1^{+}TCF-1^{+} or Tpex cells in orange; PD-1^{-}TCF-1^{+} cells in light green (Figure 1h, left dot plot). As pointed out by the reviewer, when we look at the PD-1 vs GzmB plot, the PD-1^{+}GzmB^{-} fraction contains Tpex (orange) cells with a few Tex (green) cells (Figure 1h, right dot plot). Reciprocally, the PD-1^{+}GzmB^{+} quadrant mainly contains Tex (green) cells with a few Tpex (orange) cells. In addition, the Tpex subset expresses low levels of GzmB and TIM-3, and intermediate TOX, consistent with a progenitor exhausted phenotype. Conversely, Tex cells exhibit high GzmB, TOX and TIM-3 levels, consistent with terminal exhaustion. Given that we used PD-1 and GzmB as primary gating markers and did not include TCF-1 staining in all our previous experiments, we have relabeled all relevant figures indicating PD-1^{+}GzmB^{-} and PD-1^{+}GzmB^{+} subsets, and clearly stating that they correspond primarily, but not exclusively, to Tpex and Tex cells, respectively. We hope that these modifications address the reviewer's concern and strengthen the rigor of our phenotypic analyses.

a

b

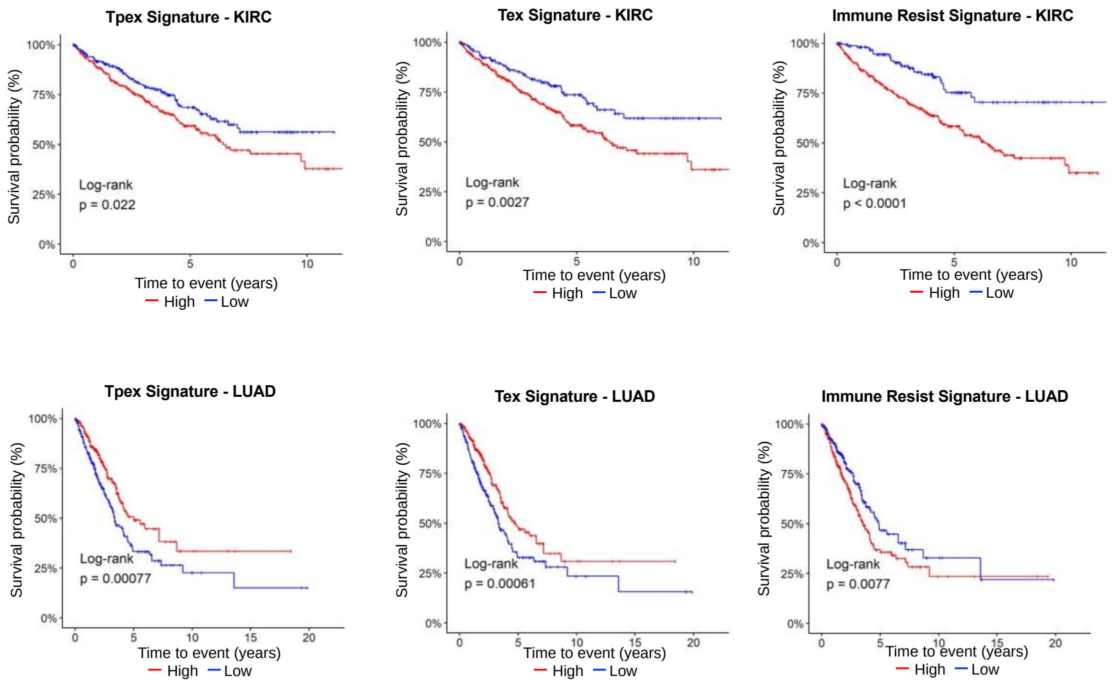

Figure for Reviewers 1. Tpex and Tex signatures correlates with poor survival in renal cell carcinoma and good survival in lung adenocarcinoma. a and **b** Kaplan–Meier overall survival curves for (a) The Cancer Genome Atlas – Kidney Renal Clear Cell Carcinoma (TCGA-KIRC) cohort, and (b) The Cancer Genome Atlas – Lung Adenocarcinoma (TCGA-LUAD) cohort, stratified by high (red) or low (blue) expression levels of indicated gene signatures. Statistical comparisons were performed using the log-rank test, with significance set at $p < 0.05$.

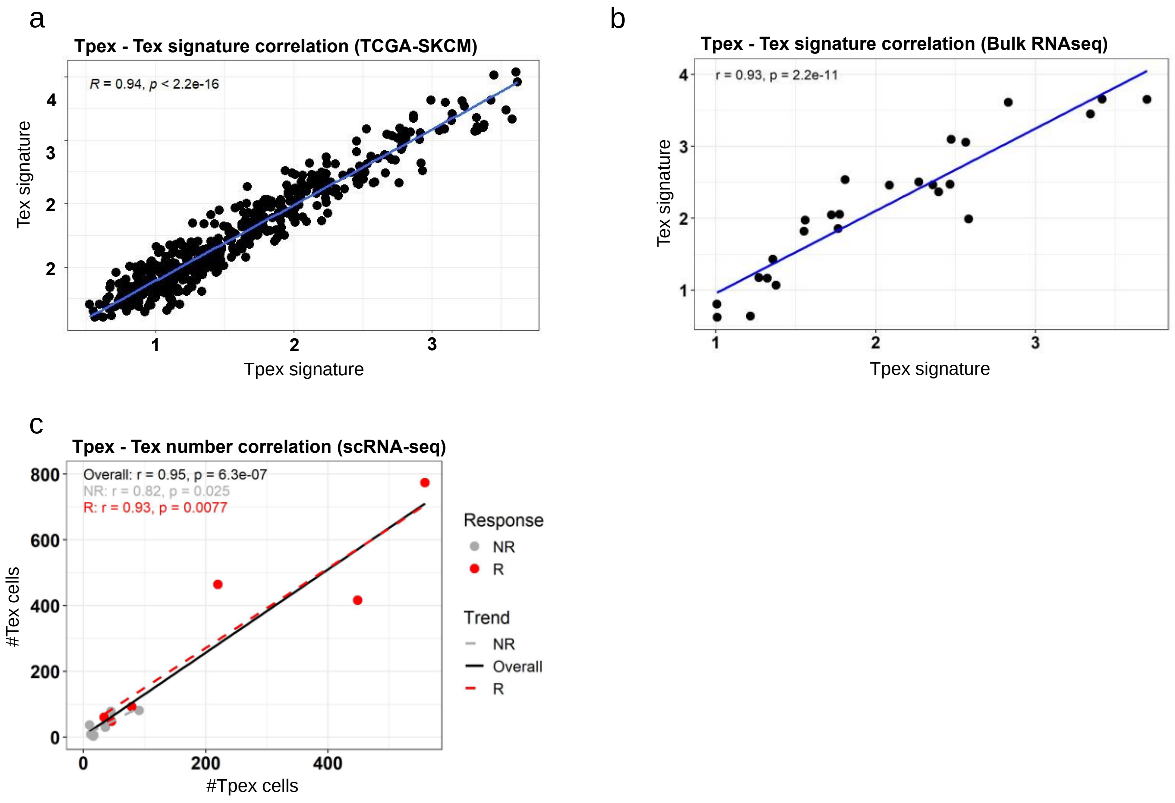

Figure for Reviewers 2. Tpex positively correlates with Tex in melanoma patients. a and **b**. correlation analysis between Tpex and Tex signatures in (a) The Cancer Genome Atlas – Skin Coutaneous Melanoma (TCGA-SKCM) and (b) TIL-treated melanoma patients. Each dot represents unique patients, and blue line indicates the trend. **c** correlation analysis between the numbers of Tpex and Tex in single-cell data from TIL-treated melanoma patients. Black line indicates the trend in all patients, grey line in No Responders (NR) and red line in Responders (R). In all panels a Pearson correlation was performed and a $p < 0.05$ was considered significant.

References

1. Nissani A, Lev-Ari S, Meirson T, Jacoby E, Asher N, Ben-Betzalel G, et al. Comparison of non-myeloablative lymphodepleting preconditioning regimens in patients undergoing adoptive T cell therapy. J Immunother Cancer. 2021 May 14;9(5).
2. Overwijk WW, Tsung A, Irvine KR, Parkhurst MR, Goletz TJ, Tsung K, et al. gp100/pmel 17 is a murine tumor rejection antigen: Induction of 'self'- reactive, tumoricidal T cells using high-affinity, altered peptide ligand. Journal of Experimental Medicine. 1998 Jul 20;188(2):277–86.
3. Yee C, Thompson JA, Byrd D, Riddell SR, Roche P, Celis E, et al. Adoptive T cell therapy using antigen-specific CD8+ T cell clones for the treatment of patients with metastatic melanoma: In vivo persistence, migration, and antitumor effect of transferred T cells. Proc Natl Acad Sci U S A. 2002 Dec 10;99(25):16168–73.
4. Johnson LA, Morgan RA, Dudley ME, Cassard L, Yang JC, Hughes MS, et al. Gene therapy with human and mouse T-cell receptors mediates cancer regression and targets normal tissues expressing cognate antigen. Blood. 2009;114(3):535–46.
5. Kansy BA, Concha-Benavente F, Srivastava RM, Jie HB, Shayan G, Lei Y, et al. PD-1 status in CD8+ T cells associates with survival and anti-PD-1 therapeutic outcomes in head and neck cancer. Cancer Res. 2017 Nov 15;77(22):6353–64.
6. Thompson RH, Dong H, Lohse CM, Leibovich BC, Blute ML, Cheville JC, et al. PD-1 is expressed by tumor-infiltrating immune cells and is associated with poor outcome for patients with renal cell carcinoma. Clinical Cancer Research. 2007 Mar 15;13(6):1757–61.
7. Sun S, Fei X, Mao Y, Wang X, Garfield DH, Huang O, et al. PD-1+ immune cell infiltration inversely correlates with survival of operable breast cancer patients. Cancer Immunology, Immunotherapy. 2014;63(4):395–406.
8. Kang MJ, Kim KM, Bae JS, Park HS, Lee H, Chung MJ, et al. Tumor-infiltrating PD1-positive lymphocytes and FoxP3-positive regulatory T cells predict distant metastatic relapse and survival of clear cell renal cell carcinoma. Transl Oncol. 2013;6(3):282–9.
9. Muenst S, Soysal SD, Gao F, Obermann EC, Oertli D, Gillanders WE. The presence of programmed death 1 (PD-1)-positive tumor-infiltrating lymphocytes is associated with poor prognosis in human breast cancer. Breast Cancer Res Treat. 2013 Jun;139(3):667–76.
10. Chow A, Perica K, Klebanoff CA, Wolchok JD. Clinical implications of T cell exhaustion for cancer immunotherapy. Vol. 19, Nature Reviews Clinical Oncology. Springer Nature; 2022. p. 775–90.
11. Terranova-Barberio M, Pawlowska N, Dhawan M, Moasser M, Chien AJ, Melisko ME, et al. Exhausted T cell signature predicts immunotherapy response in ER-positive breast cancer. Nat Commun. 2020 Dec 1;11(1).
12. Chalabi M, Fanchi LF, Dijkstra KK, Van den Berg JG, Aalbers AG, Sikorska K, et al. Neoadjuvant immunotherapy leads to pathological responses in MMR-proficient and MMR-deficient early-stage colon cancers. Nat Med. 2020 Apr 1;26(4):566–76.
13. Chow A, Uddin FZ, Liu M, Dobrin A, Nabet BY, Mangarin L, et al. The ectonucleotidase CD39 identifies tumor-reactive CD8+ T cells predictive of immune checkpoint blockade efficacy in human lung cancer. Immunity. 2023 Jan 10;56(1):93-106.e6.
14. Daud AI, Loo K, Pauli ML, Sanchez-Rodriguez R, Sandoval PM, Taravati K, et al. Tumor immune profiling predicts response to anti-PD-1 therapy in human melanoma. Journal of Clinical Investigation. 2016 Sep 1;126(9):3447–52.
15. Attrill GH, Owen CN, Ahmed T, Vergara IA, Colebatch AJ, Conway JW, et al. Higher proportions of CD39+ tumor-resident cytotoxic T cells predict recurrence-free survival in patients with stage III melanoma treated with adjuvant immunotherapy. J Immunother Cancer. 2022 Jun 10;10(6).
16. Gros A, Robbins PF, Yao X, Li YF, Turcotte S, Tran E, et al. PD-1 identifies the patient-specific CD8+ tumor-reactive repertoire infiltrating human tumors. Journal of Clinical Investigation. 2014;124(5):2246–59.
17. Li H, van der Leun AM, Yofe I, Lubling Y, Gelbard-Solodkin D, van Akkooi ACJ, et al. Dysfunctional CD8 T Cells Form a Proliferative, Dynamically Regulated Compartment within Human Melanoma. Cell. 2019 Feb 7;176(4):775-789.e18.
18. Oliveira G, Stromhaug K, Klaeger S, Kula T, Frederick DT, Le PM, et al. Phenotype, specificity and avidity of antitumour CD8+ T cells in melanoma. Nature. 2021 Aug 5;596(7870):119–25.

19. Sade-Feldman M, Yizhak K, Bjorgaard SL, Ray JP, de Boer CG, Jenkins RW, et al. Defining T Cell States Associated with Response to Checkpoint Immunotherapy in Melanoma. Cell. 2018 Nov 1;175(4):998-1013.e20.
20. Miller BC, Sen DR, Al Abosy R, Bi K, Virkud Y V., LaFleur MW, et al. Subsets of exhausted CD8+ T cells differentially mediate tumor control and respond to checkpoint blockade. Nat Immunol. 2019 Mar 1;20(3):326–36.
21. Jansen CS, Prokhnevska N, Master VA, Sanda MG, Carlisle JW, Bilen MA, et al. An intra-tumoral niche maintains and differentiates stem-like CD8 T cells. Nature. 2019 Dec 19;576(7787):465–70.
22. Gebhardt T, Park SL, Parish IA. Stem-like exhausted and memory CD8+ T cells in cancer. Vol. 23, Nature Reviews Cancer. Nature Research; 2023. p. 780–98.
23. Jerby-Arnon L, Shah P, Cuoco MS, Rodman C, Su MJ, Melms JC, et al. A Cancer Cell Program Promotes T Cell Exclusion and Resistance to Checkpoint Blockade. Cell. 2018 Nov 1;175(4):984-997.e24.
24. Boulch M, Cazaux M, Cuffel A, Guerin M V., Garcia Z, Alonso R, et al. Tumor-intrinsic sensitivity to the pro-apoptotic effects of IFN-γ is a major determinant of CD4+ CAR T-cell antitumor activity. Nat Cancer. 2023 Jul 1;4(7):968–83.
25. Spear P, Barber A, Rynda-Apple A, Sentman CL. Chimeric Antigen Receptor T Cells Shape Myeloid Cell Function within the Tumor Microenvironment through IFN-γ and GM-CSF. The Journal of Immunology. 2012 Jun 15;188(12):6389–98.
26. Boulch M, Cazaux M, Loe-Mie Y, Thibaut R, Corre B, Lemaître F, et al. A cross-talk between CAR T cell subsets and the tumor microenvironment is essential for sustained cytotoxic activity [Internet]. Vol. 6, Sci. Immunol. 2021. Available from: https://www.science.org
27. Finkelstein S, Klebanoff C, Antony P, Gattinoni L, Heimann D, Hinrichs C, et al. IFN-gamma production by adoptively transferred CD8+ T lymphocytes and host cells contributes to successful tumor immunotherapy. J Am Coll Surg. 2004 Sep 1;199(3):90–1.
28. Larson RC, Kann MC, Bailey SR, Haradhvala NJ, Llopis PM, Bouffard AA, et al. CAR T cell killing requires the IFNγR pathway in solid but not liquid tumours. Nature. 2022 Apr 21;604(7906):563–70.
29. Alizadeh D, Wong RA, Gholamin S, Maker M, Aftabizadeh M, Yang X, et al. IFNγ is critical for CAR T cell–mediated myeloid activation and induction of endogenous immunity. Cancer Discov. 2021 Sep 1;11(9):2248–65.
30. Laidlaw BJ, Craft JE, Kaech SM. The multifaceted role of CD4+ T cells in CD8+ T cell memory. Vol. 16, Nature Reviews Immunology. Nature Publishing Group; 2016. p. 102–11.
31. Laidlaw BJ, Zhang N, Marshall HD, Staron MM, Guan T, Hu Y, et al. CD4+ T Cell Help Guides Formation of CD103+ Lung-Resident Memory CD8+ T Cells during Influenza Viral Infection. Immunity. 2014 Oct 16;41(4):633–45.
32. Kalams SA, Walker BD. The critical need for CD4 help in maintaining effective cytotoxic T lymphocyte responses. Vol. 188, Journal of Experimental Medicine. The Rockefeller University Press; 1998. p. 2199–204.
33. Duell J, Leipold A, Eisele F, Afrin Mildred Scheel N, Grundheber Mildred Scheel Early L, Weinhold N, et al. Sequential Antigen-loss and Branching Evolution in Lymphoma after CD19-and CD20-Targeted T-cell Redirecting Therapy. Available from: https://doi.org/10.21203/rs.3.rs-2762036/v1
34. Majzner RG, Mackall CL. Tumor antigen escape from car t-cell therapy. Vol. 8, Cancer Discovery. American Association for Cancer Research Inc.; 2018. p. 1219–26.
35. Khan O, Giles JR, McDonald S, Manne S, Ngiow SF, Patel KP, et al. TOX transcriptionally and epigenetically programs CD8+ T cell exhaustion. Nature [Internet]. 2019 Jul 11 [cited 2021 Apr 14];571(7764):211–8. Available from: https://www.nature.com/articles/s41586-019-1325-x
36. Sekine T, Perez-Potti A, Nguyen S, Gorin JB, Wu VH, Gostick E, et al. TOX is expressed by exhausted and polyfunctional human effector memory CD8+ T cells. Sci Immunol [Internet]. 2020 Jul 1 [cited 2021 Apr 14];5(49). Available from: https://immunology.sciencemag.org/content/5/49/eaba7918
37. Utzschneider DT, Charmoy M, Chennupati V, Pousse L, Ferreira DP, Calderon-Copete S, et al. T Cell Factor 1-Expressing Memory-like CD8+ T Cells Sustain the Immune Response to Chronic Viral Infections. Immunity. 2016;45(2):415–27.
38. Beltra JC, Manne S, Abdel-Hakeem MS, Kurachi M, Giles JR, Chen Z, et al. Developmental Relationships of Four Exhausted CD8+ T Cell Subsets Reveals Underlying Transcriptional and Epigenetic Landscape Control Mechanisms. Immunity. 2020 May 19;52(5):825-841.e8.
39. Kallies A, Zehn D, Utzschneider DT. Precursor exhausted T cells: key to successful immunotherapy? Vol. 20, Nature Reviews Immunology. Nature Research; 2020. p. 128–36.

40. Siddiqui I, Schaeuble K, Chennupati V, Fuertes Marraco SA, Calderon-Copete S, Pais Ferreira D, et al. Intratumoral Tcf1 + PD-1 + CD8 + T Cells with Stem-like Properties Promote Tumor Control in Response to Vaccination and Checkpoint Blockade Immunotherapy. Immunity. 2019 Jan 15;50(1):195-211.e10.

We sincerely thank the editor and reviewers for their positive evaluation of our revised manuscript (NCOMMS-23-31003B). We found the additional comments from Reviewers 3 and 4 to be thoughtful and constructive, and we have carefully addressed them in this new revision. We have now clearly limited the scope of our study to animal models and emphasized the need for confirmation in human settings. We have restricted our conclusions regarding cross-presentation to the elimination of primary tumors and have clearly distinguished these findings from the rechallenge experiments. In addition, we have incorporated new data analyzing tissue-resident markers in host $CD8^+$ T cells and have improved gating strategies. We hope that the revised manuscript is now suitable for publication in *Nature Communications*, and we thank you once again for the opportunity to further refine our work.

A detailed, point-by-point response to all reviewer comments is provided below. To facilitate review, we have highlighted the added/changed text in yellow and reorganized paragraphs in green in the revised manuscript.

REVIEWER COMMENTS

Reviewer #1 (Remarks to the Author):

The MS has been improved by adding animal experiments. I have no further comments.

We are very glad to have addressed all the reviewer comments.

Reviewer #3 (Remarks to the Author):

Although the authors have responded to my questions the animal models are quite artificial and various lympho-depleting chemotherapies have been tested in ACTs in humans. The authors should note that their data is only validated in mouse models (also in the title) and this finding has to be analyzed in patients.

We are glad to have addressed all reviewers' questions and agree on the importance of limiting the scope of our study to mouse models and the need to confirm these findings in patients. We have modified the manuscript accordingly across the title, abstract, results and conclusions.

The authors also claim the cross-presentation is involved but only interference with TNFalpha pathway is experimentally shown. I believe that the authors should re-write their conclusions on this set of experiments.

We thank the reviewer for highlighting this important point. We limited the conclusions of cross-presentation exclusively to the elimination of primary tumors. To clearly differentiate these results from rechallenge experiments, we have separated all rechallenge experiments from previous Figures 2 and 3 in new Figure 4. We have also re-written the conclusion accordingly. These changes have significantly improved the quality of our manuscript.

Reviewer #4 (Remarks to the Author):

After reviewing this, I think the author did address all the previous reviewer's questions. This study provides compelling evidence that durable antitumor responses to adoptive $CD8^+$ T cell therapy depend on the host immune system and are mediated through a TNF α–driven cDC1–$CD8^+$ T cell axis. It also highlights the unintended consequence of lymphodepleting preconditioning on long term immunity. The work confirmed many previous studies reveal the impact of ACT on endogenous anti-tumor T cell responses and has potential implications for improving clinical ACT protocols.

We are glad that the reviewer considers that we have addressed all the previous reviewer's questions and thank the reviewer for highlighting key findings and potential implications of our study. Also, we thank the reviewer for the additional comments, which are addressed below.

The authors could broaden the translational impact by exploring following area.

• The phenotypic analysis focused primarily on T cells (transferred vs host $CD8^+$ T cells) and dendritic cells. While this panel is extensive for T-cell exhaustion states, the study gave less attention to other immune subsets. For example, the authors note that lymphodepletion caused a "profound reduction across all $CD45^+$ immune cell populations" in tumors, but the gating strategy for subsets like myeloid cells, NK cells, or B cells is not shown in detail. The characterization of other cells (e.g. MDSCs defined by CD11b+Gr1+, or tumor-associated macrophages) need improvement.

We acknowledge that our analyses were primarily focused on the DC and $CD8^+$ T cell compartments and agree that additional markers can improve the characterization of other immune cells. To this end, we performed a new set of lymphodepletion experiments using a more extensive flow cytometry panel to expand our phenotypic analysis. In the new Supplementary Figure 9, our analysis now includes the following additional populations: NK cells, B cells, monocytes, neutrophils, and tumor-associated macrophages. We believe that these additions have substantially improved the characterization of the population changes induced by lymphodepletion.

• The study's assessment of long-term immunity was mainly through a single tumor rechallenge after primary tumor clearance. Mice that eradicated B16-OVA tumors were rechallenged with wild-type B16 (lacking OVA) to test memory response to antigen-loss variants. While this demonstrated the protection, it's unclear the impact on memory CD8 T cell differentiation. Can transferred or endogenous CD8 T cells become tissue resident memory cells?

We thank the reviewer for this insightful comment and agree that characterizing memory differentiation of $CD8^+$ T cells could broaden the translational impact of our study. To this end, we performed a new set of experiments analyzing the expression of memory markers in host $CD8^+$ T cells, including CD44, CD62L, CD39, CD69, and CD103. We observed that PD-1^+ populations expressed higher levels of CD44, CD69, and CD39 than the PD-1^- population, with the PD-1^+ $GzmB^+$ population expressing the highest levels, consistent with a tissue-resident memory (Trm) phenotype, as described in mice (doi:10.4049/jimmunol.2400151). These results indicate that $CD8^+$ T cells expanded by adoptive T-cell therapy acquire a Trm phenotype, which has been largely associated with enhanced antitumor function.
